# Expected and Unexpected “Guests” at the Active Site of Human Orotidine 5′-Monophosphate Decarboxylase

**DOI:** 10.1021/acs.biochem.5c00459

**Published:** 2025-10-08

**Authors:** Laura Liliana Kirck, Elisa Santagostino, Laurin Brandhoff, Nadja A. Simeth, Kai Tittmann

**Affiliations:** † Department of Molecular Enzymology, Göttingen Center of Molecular Biosciences and Albrecht-von-Haller Institute, 9375 Georg-August University Göttingen, Julia-Lermontowa-Weg 3, D-37077 Göttingen, Germany; ‡ Max-Planck-Institute for Multidisciplinary Sciences, Am Fassberg 11, D-37077 Göttingen, Germany; § Institute for Organic and Biomolecular Chemistry, Georg-August University Göttingen, Tammannstr. 2, D-37077 Göttingen, Germany; ∥ Cluster of Excellence “Multiscale Bioimaging: from Molecular Machines to Networks of Excitable Cells” (MBExC), University of Göttingen, D-37075 Göttingen, Germany

## Abstract

With an extraordinary rate enhancement of 10^17^ compared to the uncatalyzed reaction and no need for a cofactor, orotidine 5′-monophosphate decarboxylase (OMPDC) is considered one of the most efficient enzymes. Its mechanism has fascinated researchers for over 50 years. In this study, we used high-resolution X-ray crystallography to examine the molecular interactions between the active site of human OMPDC and various natural and synthetic ligands, including transition-state and product analogues, at the atomic level. Additionally, we evaluated their binding affinities with isothermal titration calorimetry (ITC). During protein expression and subsequent structure analysis, we identified nucleotides xanthosine-5′-monophosphate (XMP) and thymidine-5′-monophosphate (dTMP) bound to the active sites of OMPDC and its Thr321Asn variant, respectively, and confirmed their high binding affinities through ITC. Chemically, we investigated the role of the ribose 2′–OH group using 2′-deoxy OMP and 2′-SH UMP, focusing on validating key binding interactions within the nucleoside moiety. To further explore these interactions, we modified the heterocycles (e.g., GMP and CMP) and synthesized a new transition-state analogue, cyanuryl-5′-monophosphate (YMP). YMP exhibited strong affinity for OMPDC and formed an additional hydrogen bond with a nearby water molecule. However, this enthalpically favorable interaction resulted in an entropic penalty compared to the best-known OMPDC inhibitor, BMP, leading to similar affinities. To address this, we synthesized 5-methyl OMP to further improve ligand-enzyme interactions. This modification enhanced stabilization within the hydrophobic pocket through van der Waals forces, paving the way for designing more effective OMPDC inhibitors with specific substitutions aimed at optimizing binding affinity and enzyme inhibition.

## Graphical abstract

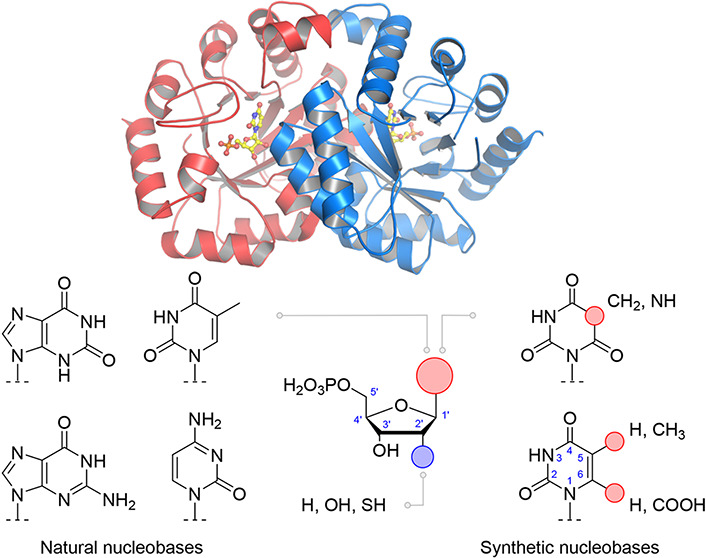

## Introduction

Orotidine 5′-monophosphate decarboxylase (OMPDC) converts orotidine 5′-monophosphate (OMP) into uridine 5′-monophosphate (UMP) and CO_2_ during the final step of de novo pyrimidine synthesis, which is essential for producing DNA and RNA nucleotides.^1^ Recognized as one of the most efficient enzymes, OMPDC accelerates the decarboxylation rate by a factor of 10^17^,^2^ and exhibits a net transition-state stabilization (Δ*G* ^#^) of 31 kcal/mol.^3^ Its unique ability lies in achieving this substantial enhancement without the aid of bioorganic cofactors or metal ions, relying solely on interactions between the substrate OMP and residues at the active site.^4^ OMPDC is present across all domains of life with minor variations in its primary structure. However, the key catalytic amino acids at the active site are highly conserved, including two lysines (K281, K314) and two aspartic acids (D312, D317), which form the so-called catalytic tetrad in the sequence Asp317-Lys314-Asp312-Lys281 (numbering from the human enzyme).^5^

OMPDC is considered a potential target for anticancer drugs,^6^ West Nile virus,^7^ and malaria.^8^ Despite numerous studies and crystal structures leading to various mechanistic proposals, an agreement on the exact mechanism has not yet been reached.^9–14^ The binding contributions of the ribosyl ring (Δ*G* ^⧧^: 10 kcal/mol) and the phosphodianion (Δ*G* ^⧧^: 12 kcal/mol) to catalytic activation have been emphasized, leading to the proposal that catalysis involves a conformational transition of the enzyme from an open to a closed complex.^12,15–17^ The estimated 12 kcal/mol phospodianion contribution to transition-state stabilization, further highlights its central role in OMPDC proficiency.^17^ However, this induced-fit model has been challenged by computational studies, which suggest that interactions with the phosphate group primarily reduce the overall reorganization energy of the reaction. Accordingly, the mechanism is attributed to preorganization and transition-state stabilization,^11,18^ rather than to an induced-fit effect.^14^ Lately, high-resolution X-ray crystallography and QM/MM studies have suggested a more general view of the OMPDC mechanism. Accordingly, the substrate carboxylate leaving group is not electrostatically stressed at the active site as previously thought. Instead, it is stabilized through a low-barrier hydrogen bond with Asp312, which initiates a cascade of proton transfers. The residues surrounding the substrate stabilize the transition state primarily through electrostatic and dipole interactions, while also promoting the formation of a pronounced out-of-plane distortion of the scissile substrate C–C bond in terms of reactant-state destabilization.^13^

To gain a deeper understanding of the structural basis of substrate recognition and binding, we studied various known and novel modified UMP and OMP derivatives in greater detail using X-ray crystallography and isothermal titration calorimetry (ITC). Two of the most well-known ligands are barbituric acid 5′-monophosphate (BMP) and 6*-*aza-UMP due to their resemblance to the presumed carbanionic transition state.^19–22,13^

As shown in Figure 1, BMP binds to the active site of the human enzyme in the *syn*-conformation, while aza-UMP adopts the *anti*-conformation. Therefore, we opted to synthesize and test an analog with cyanuric acid as a symmetrical base, serving as a novel transition-state analog that offers H-bond interactions to both structural faces. In this paper, we present the structural and thermodynamic characterization of the binding of the inhibitor 1-(β-d-ribofuranosyl) cyanuric acid-5′-monophosphate (YMP) to human OMPDC head-to-head with BMP (Figure 1).

**Figure 1:**
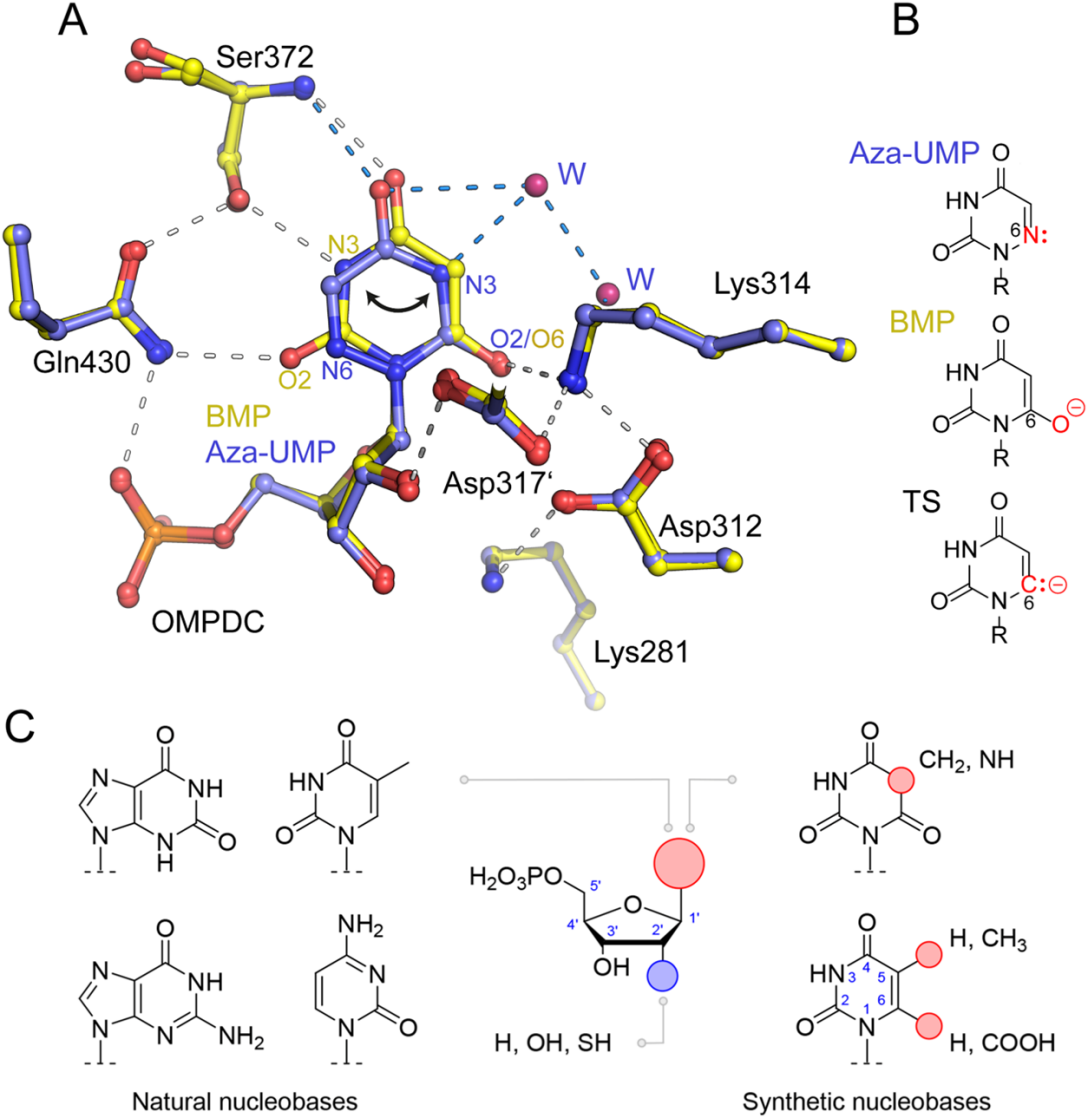
Substrate and transition-state analogs of OMPDC. (A) Active site structure of human OMPDC in complex with BMP (PDB: 7OTU, in yellow) and with aza-UMP (PDB: 6zx1, in blue). Hydrogen-bond interactions are indicated with dashes. Note that BMP adopts the *syn*-conformation, while aza-UMP binds in the *anti*-conformation. Note further the presence of two water (W) molecules in the structure with aza-UMP, which are part of a network that includes the substrate (atom N3) and Lys314. (B) Chemical structures of aza-UMP, BMP, and the presumed carbanionic transition state (TS) of OMP. (C) Chemical structures of human OMPDC ligands analyzed in this work.

Furthermore, we discovered that specific nucleotides copurified with OMPDC during the protein expression and purification process. Unexpectedly, high-resolution crystal structures showed xanthosine-5′-monophosphate (XMP) in the human OMPDC wild-type and thymidine-5′-monophosphate (dTMP) at the active site of the variant OMPDC Thr321Asn. We analyzed the binding of these ligands and chemically related analogs. We examined the interactions of different nucleobases, including guanosine-5′-monophosphate (GMP) as a purine base ligand. Additionally, we studied cytidine-5′-monophosphate (CMP), a pyrimidine derivative, to gain a deeper understanding of the role of the carbonyl at C4 by altering the hydrogen bond acceptor to a donor (amino group). For XMP, TMP, and CMP, we were able to report the dissociation constants for the human OMPDC system for the first time. We also successfully visualized all three nucleotides bound in the active site of human OMPDC through X-ray crystallography. Furthermore, the unexpected observation of dTMP opened up the possibility of systematically exploring different binding motifs. First, we considered adding a nonpolar methyl group at C5 (as in the thymine base), which led to the synthesis of 5-methyl OMP (5-Me OMP), a “hybrid” of TMP and OMP. In addition, we examined the role of 2′–OH, given that dTMP is missing this group, and the ribosyl hydroxy interacts with the amino acids Thr321′ and Asp317′ of the neighboring subunit.^23^ Thus, we synthesized 2′-deoxy OMP and 2′-deoxy UMP, and further replaced the 2′–OH group with a larger and more polarizable group (2′-SH UMP) (Figure 1).

## Materials and Methods

### OMPDC Expression and Purification

Expression and purification of OMPDC wild-type were performed according to the published protocol by Rindfleisch et al.^13^ An additional purification step was implemented before size exclusion chromatography (S75) by incubating the protein with 0.25 U/mg of alkaline phosphatase and 1x AP buffer. The final OMPDC concentration was measured spectrophotometrically using the molar extinction coefficient of ε_280_ = 15,649 M^–1^ cm^–1^ at 280 nm. The protein was directly used for structural and kinetic studies. OMPDC Thr321Asn was generated via site-directed mutagenesis using the following primers:

T321N_Fwd: GCA GAT ATA GGA AAC AAC GTG AAA AAG CAG

T321N_Rev: CTG CTT TTT CAC GTT GTT TCC TAT ATC TGC

Purification of the variant was performed as stated above.

### Isothermal Titration Calorimetry

Steady-state kinetics of OMPDC conversion and thermodynamics of ligand binding were conducted using a PEAK-ITC instrument (Malvern Panalytical) in 20 mM HEPES/NaOH, pH 7.4, at 25 °C. The substrate OMP trisodium salt and the ligands UMP, GMP, XMP, and TMP were purchased from Sigma-Aldrich (purity >99%). BMP, YMP, 2′-deoxy OMP and 2′-deoxy UMP, 5-methyl OMP, 2′-SH UMP, and CMP were synthesized by us (for detailed synthetic procedures and compound characterization, see Supporting Information).

For steady-state kinetic analysis, 20 μM OMPDC and 1 mM OMP were used at a cell temperature of 25 °C. The reference power was set to 8 μcal/s, the stirring power to 750 rpm, and two injections of 10 μL each were performed. For competitive inhibition studies, the substrate concentration remained the same while the ligand concentration was varied. The experiments are summarized below:

OMPDC with OMP and BMP: 18 μM OMPDC, 1 mM OMP, BMP (50, 100, 200 and 300 nM), buffer (HEPES 0.02 M, pH 7.4), reference power ITC 8 μcal/s, stirring speed 750 rpm, cell temperature 25 °C, 2 injections of 10 μL each.

OMPDC with OMP and YMP: 21 μM OMPDC, 1 mM OMP, YMP (200, 300, 400 and 500 nM), buffer (HEPES 0.02 M, pH 7.4), reference power ITC 8 μcal/s, stirring speed 750 rpm, cell temperature 25 °C, 2 injections of 10 μL each.

OMPDC with OMP and XMP: 20 μM OMPDC, 1 mM OMP, XMP (30, 50, 100 and 120 μM), buffer (HEPES 0.02 M, pH 7.4), reference power ITC 8 μcal/s, stirring speed 750 rpm, cell temperature 25 °C, 2 injections of 10 μL each.

OMPDC with OMP and 5-methyl OMP: 70/100 μM OMPDC, 1 mM OMP, 5-methyl OMP (1 mM), buffer (HEPES 0.02 M, pH 7.4), reference power ITC 8 μcal/s, stirring speed 750 rpm, cell temperature 25 °C, 2 injections of 10 μL each.

OMPDC with OMP: 20 μM OMPDC, 1 mM OMP, buffer (HEPES 0.02 M, pH 7.4), reference power ITC 8 μcal/s, stirring speed 750 rpm, cell temperature 25 °C, 2 injections of 10 μL each.

The data sets were evaluated via Microsoft Excel, and the decarboxylation rates were fitted in OriginPro 2020 according to the Hill equation. The resulting thermograms of BMP and YMP are shown in Figure S10.

Isothermal titration calorimetry (ITC) is used to determine the dissociation constant (*K*_D_) of a protein–ligand interaction by measuring the heat released or absorbed during binding. In practice, the ligand is sequentially titrated into a solution containing the protein in the reaction cell. As the binding sites become saturated over time, no additional heat changes are detected. The resulting thermogram is then converted into a binding isotherm, which can be used to calculate all relevant thermodynamic parameters. Thereby, the (*K*_D_) follows the general reaction, where the enzyme (E), by addition of a ligand (L), forms an enzyme-ligand complex (EL)

$$E+L\overset{K_{a}}{↔}\mathrm{EL}$$

The association constants can then be used to determine the dissociation constant^24,25^

1 $$K_{a}=1/K_{D}$$

Here, we measured the tested ligands in triplicate. The following parameters for the binding experiments were used:

OMPDC with UMP: 195 μM OMPDC, 2.08 mM UMP, buffer (HEPES 0.02 M, pH 7.4), reference power ITC 8 μcal/s, stirring speed 750 rpm, cell temperature 25 °C, one injection of 0.4 μL and 18 injections of 2 μL UMP, 120 s spacing.*

OMPDC with BMP: 192 μM OMPDC, 2.28 mM BMP, buffer (HEPES 0.02 M, pH 7.4), reference power ITC 11 μcal/s, stirring speed 750 rpm, cell temperature 25 °C, one injection of 0.4 μL and 18 injections of 2 μL BMP, 120 s spacing.

OMPDC with 5-methyl OMP: 195 μM OMPDC, 4.61 mM 5-methyl OMP, buffer (HEPES 0.02 M, pH 7.4), reference power ITC 10 μcal/s, stirring speed 750 rpm, cell temperature 25 °C, 1 injection of 0.4 μL and 18 injections of 2 μL 5-methyl OMP, 120 s spacing.*

OMPDC with dTMP: 186 μM OMPDC, 5 mM dTMP, buffer (HEPES 0.02 M, pH 7.4), reference power ITC 8 μcal/s, stirring speed 750 rpm, cell temperature 25 °C, 1 injection of 0.4 μL and 18 injections of 2 μL dTMP, 120 s spacing.

OMPDC T321N′ with dTMP: 211 μM OMPDC T321N′, 2 mM dTMP, buffer (HEPES 0.02 M, pH 7.4), reference power ITC 10 μcal/s, stirring speed 750 rpm, cell temperature 25 °C, 1 injection of 0.4 μL and 18 injections of 2 μL dTMP, 120 s spacing.

OMPDC with XMP: 194 μM OMPDC, 1.5 mM XMP, buffer (HEPES 0.02 M, pH 7.4), reference power ITC 8 μcal/s, stirring speed 750 rpm, cell temperature 25 °C, 1 injection of 0.4 μL and 18 injections of 2 μL XMP 120 s spacing.*

OMPDC with YMP: 200 μM OMPDC, 2.03 mM YMP, buffer (HEPES 0.02 M, pH 7.4), reference power ITC 8 μcal/s, stirring speed 750 rpm, cell temperature 25 °C, 1 injection of 0.4 μL and 18 injections of 2 μL YMP, 120 s spacing.

OMPDC with CMP: 200 μM OMPDC, 1.80 mM CMP, buffer (HEPES 0.02 M, pH 7.4), reference power ITC 10 μcal/s, stirring speed 750 rpm, cell temperature 25 °C, 1 injection of 0.4 μL and 18 injections of 2 μL CMP, 120 s spacing.*

OMPDC with 2′-deoxy UMP: 211 μM OMPDC, 9.04 mM 2′-deoxy UMP, buffer (HEPES 0.02 M, pH 7.4), reference power ITC 10 μcal/s, stirring speed 750 rpm, cell temperature 25 °C, 1 injection of 0.4 μL and 18 injections of 2 μL 2′-deoxy UMP, 120 s spacing.

OMPDC with GMP: 184 μM OMPDC, 10 mM GMP, buffer (HEPES 0.02 M, pH 7.4), reference power ITC 8 μcal/s, stirring speed 750 rpm, cell temperature 25 °C, 1 injection of 0.4 μL and 18 injections of 2 μL GMP, 120 s spacing.

The raw files were analyzed using the software provided by the instrument manufacturer. Marked data sets (*) were integrated using NitPic version 1.2.7 and fitted with Sedphat version 14.0.^26^ Final graphs were created with OriginPro 2020. Isotherms are shown in Figure S11.

### OMPDC Crystallization

OMPDC crystals were grown for at least 3 days at 20 °C using the hanging drop diffusion method with Crystalgen SuperClear Plates (Jena Bioscience) as previously described.^13,27,28^ The crystals were gradually transferred to increasing concentrations of cryo-solution (100 mM Tris/HCl pH 8.0, 2 M (NH_4_)_2_SO_4_, 10 mM glutathione pH 8.0, 0.25–1 M l-proline). After the crystals were adjusted to 1 M l-proline cryo-solution, various ligands were used for soaking experiments. BMP, YMP, 5-methyl OMP, 2′-SH UMP, and CMP were dissolved at a concentration of 25 mM in cryo-solution, and soaking times varied from 30 s to 6 min. The crystals were flash-cooled in liquid nitrogen and stored at these temperatures until the diffraction experiments were conducted.

OMPDC was also cocrystallized with XMP, UMP, and GMP. For this, a final protein concentration of 4.5 mg/mL and 25 mM ligand were used in 20 mM HEPES/NaOH, pH 7.4. The solution was mixed in a 1 + 1 ratio with the reservoir solution. Plates were stored at 20 °C, and crystals grew overnight. After 1 week, crystals were transferred to cryo-solution supplemented with 25 mM XMP/UMP/GMP. The cocrystallized crystals were flash-cooled in liquid nitrogen and stored until use.

OMPDC Thr321Asn crystals were grown as described for the wild-type protein. Crystals were used after at least 3 days of growth and then gradually transferred to the cryo-solution. Once 1 M l-proline was reached, the crystals were soaked with 25 mM 2′-deoxy OMP (between 30 s and 1 h). For flash-cooling, liquid nitrogen was used.

### Data Collection and Structure Determination

All crystals were measured at the Deutsches Elektronen-Synchrotron (DESY) in Hamburg under cryogenic conditions (100 K). The beamline P14 operated by the European Molecular Biology Laboratory (EMBL) at the PETRA III storage ring was used.

The structures of OMPDC with the different ligands were determined by processing the diffraction data with the XDS program suite.^29^ Rigid-body refinement was performed via phenix.refine using the previously published OMPDC structures.^13,30^ The models were built and corrected via Coot with several refinement cycles through *phenix.refine*.^30,31^ The CIF files of the ligands were generated with Jligand and structures were corroborated with MolProbity and wwwPDB Validation Service.^32–35^ Finally, the program PyMOL was used to display the protein structures.^36^ A summary of the data collection and refinement statistics is presented in Table S2.

### Synthesis of Analogues

#### Synthesis of BMP and YMP

BMP and YMP were synthesized using the Silyl-Hilbert-Johnson reaction starting from commercially available protected sugar acetate and the accessible nucleobase.^13,37,38^ For BMP, barbituric acid was silylated beforehand. The resulting nucleosides were then deprotected with 1 M NaOH in methanol and directly treated with POCl_3_ in trimethyl phosphate to produce the corresponding phosphate esters. These esters were hydrolyzed and purified by normal-phase flash chromatography to obtain the target compounds. Both BMP and YMP were produced in 9% yield over two reaction steps.

#### Synthesis of 5-methyl OMP and 2′-Deoxy OMP

5-methyl OMP and 2′-deoxy OMP were synthesized using an *Umpolung* reaction to introduce a carboxylic group at position C6.^13^ Starting from commercially available 5-methyl uridine and 2′-deoxy uridine, the hydroxy groups were protected with isopropylidene and silyl protecting groups. The fully protected nucleosides underwent lithiation at C6 with LDA at −78 °C, followed by the addition of dry ice as an electrophile. The resulting carboxylated derivatives were then deprotected and directly phosphorylated to produce the target molecules. After normal-phase flash chromatography, 5-Me OMP was obtained in a 6% yield (over two steps). 2′-deoxy OMP was purified using anion exchange HPLC with a yield of 2%.

#### Synthesis of 2′-SH UMP

2′-SH UMP was synthesized by adapting reported procedures starting from commercially available arabinouridine.^39,40^ The 3′- and 5′–OH groups of the starting material were protected simultaneously with a tetraisopropyl disiloxane protecting group, followed by triflation of the 2′-hydroxy group. Subsequently, the triflate displacement resulted in the inversion of configuration with 4-methoxy-α-toluenethiol. The silyl-protecting group was removed using a 1 M TBAF solution in THF, followed by phosphorylation of the free 5′–OH. The final step involved deprotection of the methoxybenzyl group under acidic conditions to yield 2′-SH UMP in 48% overall yield. After normal-phase flash chromatography, the target molecule did not achieve sufficient purity for ITC experiments; therefore, only the crystal structures were analyzed to characterize its binding to the target enzyme.

#### Synthesis of 2′-Deoxy UMP and CMP

Commercially available 2′-deoxyuridine and cytidine were treated with POCl_3_ in trimethyl phosphate to give the phosphate esters, followed by hydrolysis to afford the corresponding phosphate derivatives.^41^ The latter were purified by normal-phase flash chromatography to give the target molecules. CMP and 2′-deoxy UMP were obtained in 72% and 45% yield, respectively.

Detailed experimental procedures for the synthesis of all analogs are provided in the Supporting Information.

## Results and Discussion

### Chemically Synthesized OMPDC Ligands

One of the most potent known OMPDC ligands is BMP. This inhibitor exhibits high inhibitory potency (*K*_i_ = 8.8·10^–12^ M) toward yeast OMPDC by resembling the carbanionic transition state.^21,22^ As the binding motif and affinity of ligands can vary for different OMPDC species, we assessed the potency of BMP toward human OMPDC.^42–44^ Due to the marked positive cooperativity of human OMPDC observed for OMP turnover with and without inhibitors (Figure S10), the inhibitory constant *K*_i_ could not be quantitatively determined as the graphs in the Dixon plot clearly deviate from linearity (data not shown).^13,45^ However, the thermodynamic constants of binding, such as molar enthalpy (Δ*H*), dissociation constant (*K*_D_), Gibbs energy (Δ*G*), and entropy (−*T*Δ*S*) could, in this case, be determined by isothermal titration calorimetry (ITC) measurements (Table 1). Using this approach, we estimated a *K*_D_ value of 34 ± 2 nM for the binding of BMP to human OMPDC.^20–22^ Of note, the affinity of BMP for the human enzyme is lower than that reported for yeast OMPDC, suggesting an alternative binding conformation.^21^

**Table 1:** Thermodynamic Constants of Human OMPDCase Ligands as Measured by ITC at 25 °C^a^

ligand	*K*_D_	Δ*H* (kcal/mol)	Δ*G* (kcal/mol)	*n* (stoichiometry)	–*T*Δ*S* (kcal/mol)
5-MeOMP	0.40 ± 0.22 μM	–1.20 ± 0.03	–8.78 ± 0.19	1.54 ± 0.01	–7.58 ± 0.26
XMP*	0.21 ± 0.03 μM	–1.86 ± 0.10	^b^	^b^	^b^
	0.18 ± 0.02 μM	–5.57 ± 0.07			
BMP	33.6 ± 2.2 nM	–8.74 ± 0.04	–10.2 ± 0.06	1.33 ± 0.001	–1.46 ± 0.06
YMP	57.2 ± 10.2 nM	–10.17 ± 0.06	–9.89 ± 0.02	1.08 ± 1.77 × 10^–3^	0.26 ± 0.02
dTMP	118.6 ± 8.8 μM	–4.74 ± 0.29	–5.37 ± 0.09	0.64 ± 0.03	–0.63 ± 0.88
dTMP(Thr321Asn)	0.43 ± 0.04 μM	–14.3 ± 0.1	–8.69 ± 0.08	0.64 ± 0.02	5.60 ± 0.06
CMP*	4.73 ± 1.09 μM	–2.30 ± 0.08	^b^	^b^	^b^
	8.33 ± 1.06 μM	–7.08 ± 0.56			
UMP*	2.6 ± 1.2 μM	–1.03 ± 0.06	^b^	0.98 ± 0.01	^b^
	6.7 ± 0.8 μM	–2.1 ± 0.1			

a Ligands marked with an asterisk (*) were analyzed using Sedphat version 14.0.^26^ The *S*_0.5_ value of OMPDC for substrate OMP is 30 μM (SI Figure 9).

b A determination was not possible due to a complex model of ligand binding.

Similar to BMP, aza-UMP mimics the transition state but has a nitrogen atom at the C6 position.^19,46^ Recent X-ray crystal structures showed that aza-UMP adopts an *anti*-conformation in human OMPDC, where N6 points in the opposite direction from the catalytic tetrad and the C2 oxo and N3–H groups directly interacting with it.^13^ We aimed to take advantage of this key feature by replacing C5 of BMP with a nitrogen atom. As a result, the new ligand 1-(β-d-ribofuranosyl) cyanuric acid-5′-monophosphate (YMP), was successfully synthesized. Due to the pronounced positive cooperativity and therewith complex kinetic model, the *K*_i_ could not be reliably estimated. However, we estimated a *K*_D_ of 57 ± 11 nM by thermodynamic ITC experiments, which is similar to the one obtained for BMP (Table 1).

As observed in the crystal structure (Figure 3), YMP binds to the active site in a manner similar to BMP (compare Figure 2). The phosphate gripper and pyrimidine umbrella are fully resolved, indicating a closed conformation of the enzyme. The phosphate group of YMP is stabilized by side chains Gln430, Tyr432, Gly450, and Arg451, with interatomic distances of <3 Å (Figure 3). The 2′–OH ribosyl group forms hydrogen bonds with His283, Asp317′, and Thr321′, while the 3′–OH group contacts Asp259, Lys281, and His283. Finally, the base is hydrogen-bonding to residues Lys314, Ser372, and Gln430. Additionally, and uniquely observed for YMP, N5 forms a hydrogen bond with a nearby water molecule (2.8 Å) that interacts with a second water molecule, and, through this interaction, with O6 and Lys314.

**Figure 2:**
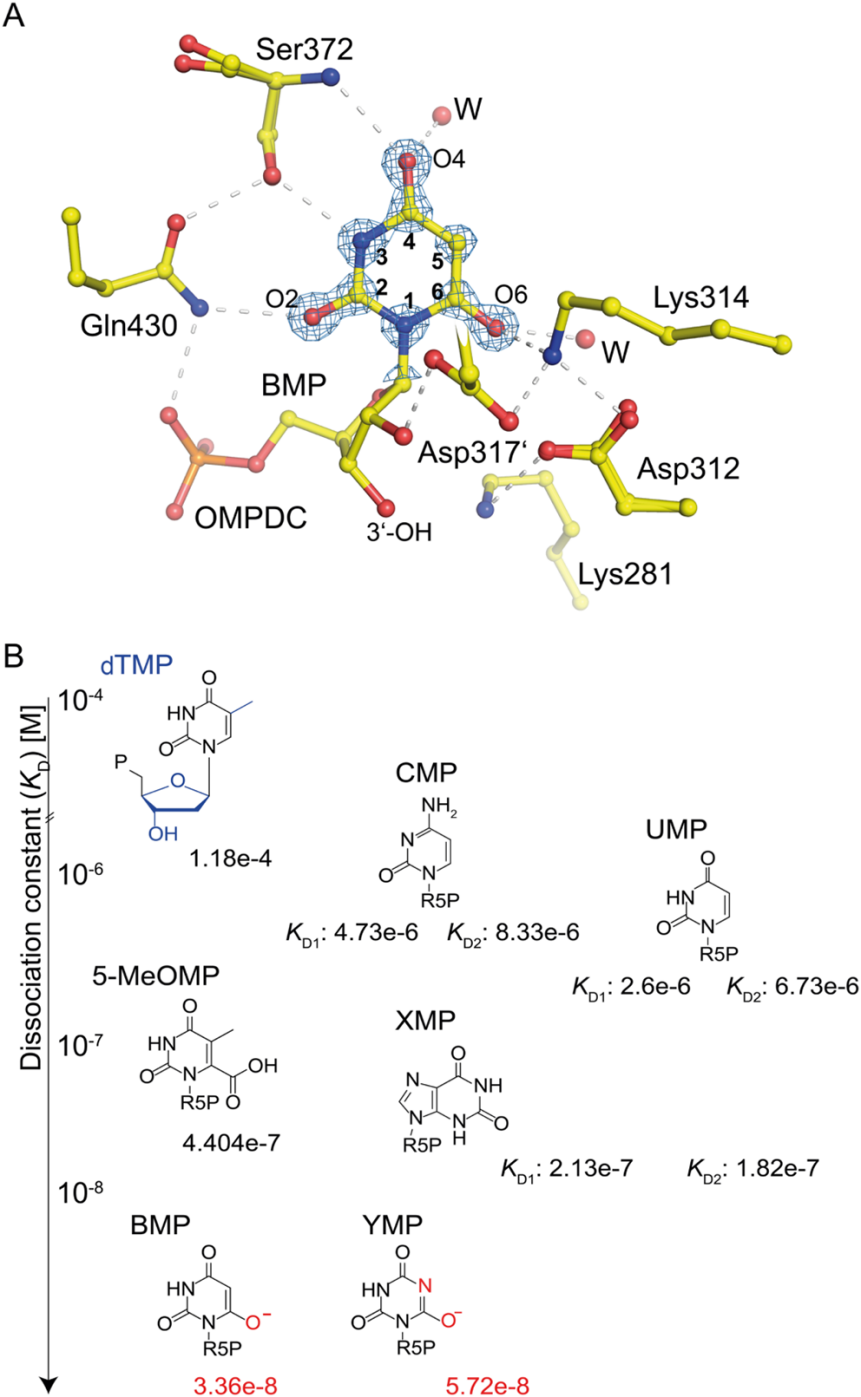
Binding constants of substrate and transition-state analogs for human OMPDC. (A) Crystal structure of human OMPDC with bound transition-state analog BMP (PDB: 7OTU). The base moiety forms hydrogen bonds with Asp312 and Lys314 from the catalytic tetrad, as well as with Ser372 and Gln430. Lys281 and Asp317′ interact with the ribosyl 2′–OH and 3′–OH groups of BMP. The pyrimidine ring is superposed with the corresponding 2mFo-DFc electron density map at a contour level of 4σ. (B) Chemical structures of characterized OMPDC ligands, sorted by their corresponding dissociation constants (in M). Note that BMP and YMP show the highest binding affinity toward human OMPDC.

**Figure 3:**
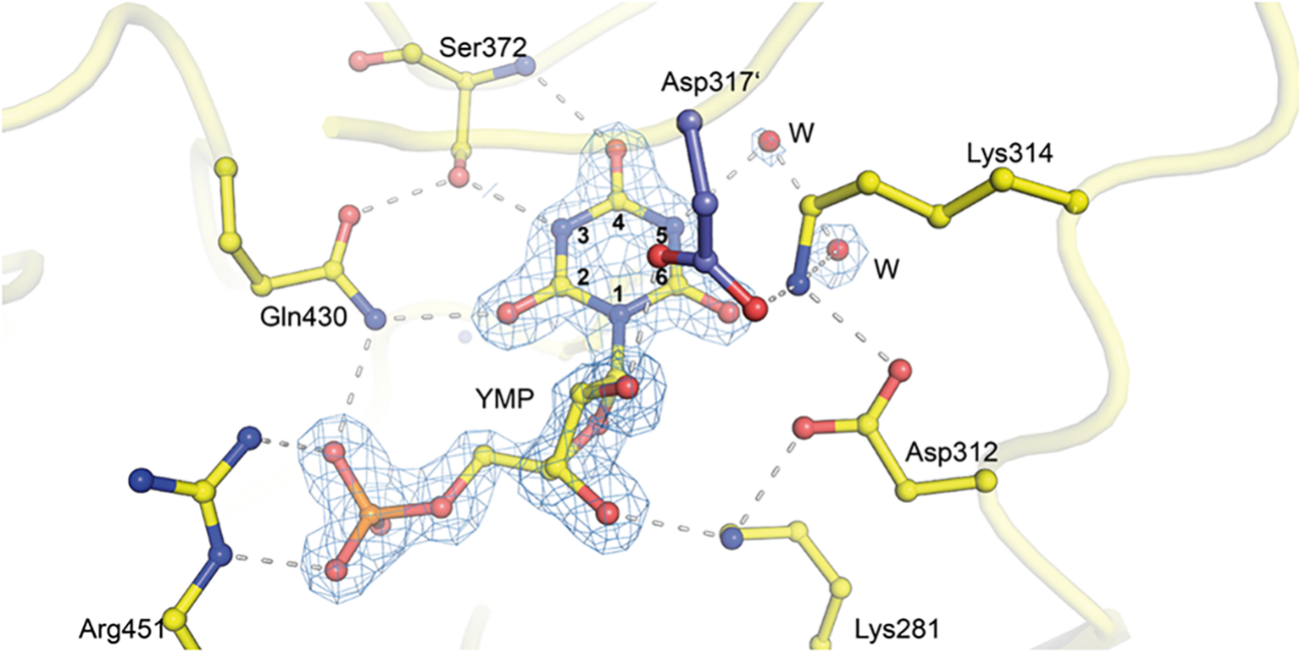
Crystal structure of transition-state analog YMP bound to human OMPDC. The crystal structure (1.5 Å resolution) shows YMP bound to the active site of human OMPDC (PDB: 9HDV). The phosphate gripper (Arg451, Gln430) and pyrimidine (Ser372) loops stabilize the enzyme’s closed conformation. The catalytic tetrad forms hydrogen bonds with O6 of the base and the 3′–OH of the ribosyl group. Note the presence of two water (W) molecules, which form hydrogen bonds with N5, O6, and Lys314. YMP and water molecules are superimposed on the corresponding 2mFo-DFc electron density map with a contour level of 2σ.

When overlaying the crystal structures of human OMPDC with BMP and YMP (Figure 4A), the only noticeable difference is two additional water molecules bridging N5 and O6, which are not seen in structures with BMP.^13^ All protein residues and the two ligands bind in exactly the same location and with the same conformation. The thermodynamic data for both ligands show stoichiometry values that differ from the expected *n* = 1 (meaning there is one binding site for OMPDC), which could be due to minor contamination. Even if we consider an error margin of ±5–10%, the overall trend in the thermodynamic data remains consistent (Table S1). Notably, the difference caused by the extra hydrogen bonds in the YMP structure is evident in the thermodynamic data. While the Gibbs free energies (Δ*G*) are similar, there is a 1.43 kcal/mol difference in the binding enthalpies (BMP Δ*H* = −8.74 ± 0.04 kcal/mol, YMP Δ*H* = −10.17 ± 0.06 kcal/mol) (Figure 4B). An additional difference appears in the entropy (−*T*Δ*S*), which is 1.72 kcal/mol higher for YMP (−*T*Δ*S*= 0.26 kcal/mol) compared to BMP (−*T*Δ*S*= −1.46 kcal/mol). The increased entropy aligns with the expected value of 1.6 kcal/mol for a typical hydrogen bond toward a water molecule.^47–49^ As a result, the bond between the water molecule(s) and YMP causes slight differences in both ΔH and −*T*Δ*S*, leading to what is known as enthalpy-entropic compensation.^49–51^ Although YMP’s enthalpic gain of binding is higher, the entropic penalty cancels this out, resulting in similar binding affinities for YMP and BMP.

**Figure 4:**
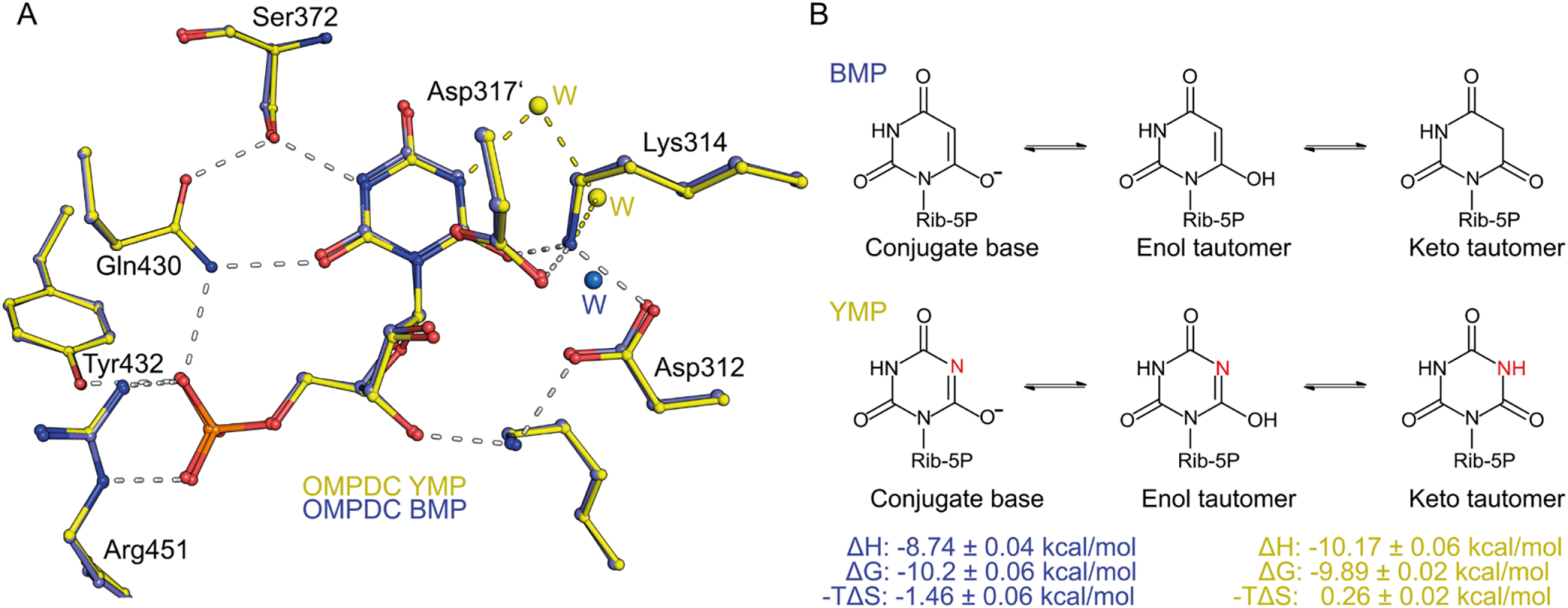
Structures of human OMPDC with transition-state analogs BMP and YMP. (A) Superposition of human OMPDC active site with bound BMP (in violet) and YMP (in yellow), showing the interacting protein residues and hydrogen bonds. Note the exclusive presence of two water (W) molecules in the YMP structure that interact with the base and Lys314. (B) Chemical structures of BMP and YMP, including protonic and tautomeric states, along with measured thermodynamic constants obtained through isothermal titration calorimetry. Due to the similarity to the presumed carbanionic transition state, the conjugate bases of YMP and BMP with negative charges at O6 are likely to be stabilized by the enzyme.

### Binding of Endogenous Purine and Pyrimidine Nucleotides

Overexpression of human OMPDC in *Escherichia coli* consistently resulted in the binding of endogenous nucleotides to the active site. This binding was so strong that the ligand remained bound during the entire purification and ensuing crystallization process.

In the crystal structure of OMPDC wild-type (Figure S1), the electron density map suggested the presence of a purine, either XMP or GMP, due to the presence of a functional group bound to C2 (Figure 5A). By measuring the binding affinities of both purines toward OMPDC, we could clearly exclude GMP, as no measurable binding heat was detected by ITC (Figure S11). On the other hand, XMP has been reported to inhibit OMPDC from different organisms.^42,44,46,52^ A structure of human OMPDC in complex with XMP at a resolution of 1.80 Å has been deposited in the PDB (PDB ID 3BVJ) but was not discussed in a publication. Notwithstanding this, we crystallized human OMPDC in complex with XMP, and solved the structure at a resolution of 1.0 Å. The electron density maps were well-defined for ligand XMP and the surrounding protein residues, allowing for a reliable model building (Figure 5). In contrast to MtOMPDC, the pyrimidine and phosphate gripper loops are fully resolved, indicating a closed enzyme conformation.^42,44^ Overall, the binding pose is similar to that of product UMP (Figure 5C). The binding affinity of XMP, as measured by ITC, revealed mild cooperativity in the dimer, as two dissociation constants were obtained with values of *K*_D1_ = 218 ± 32 nM and *K*_D2_ = 182 ± 18 nM (Table 1). Notably, the average concentration of XMP amounts to 3.5 μM in vivo, suggesting a putative regulation of OMPDC in a “crosstalk” between pyrimidine and purine biosynthesis.^53^ Despite our failure to detect the binding of GMP to human OMPDC using ITC, we successfully crystallized the enzyme in the presence of GMP and solved its structure. However, structure analysis of several single crystals clearly revealed that no ligand was bound to the active site (data not shown). Thus, human OMPDC does not bind GMP with high affinity in solution or *in crystallo*. From a chemical perspective, the differential binding of XMP and GMP is remarkable, as the only difference between the two nucleotides is the substituent at C2 of the purine ring (oxo group in case of XMP; amino group in GMP).^34,49,50^ The two substituents are thus either hydrogen-bond acceptors (XMP) or hydrogen-bond donors (GMP). It seems likely that the high preference of human OMPDC for XMP is encoded in the H-bond interaction of the 2-oxo group with residues Lys314 (H-bond donor) and, via a water, Asn341 (Figure 5D).

**Figure 5:**
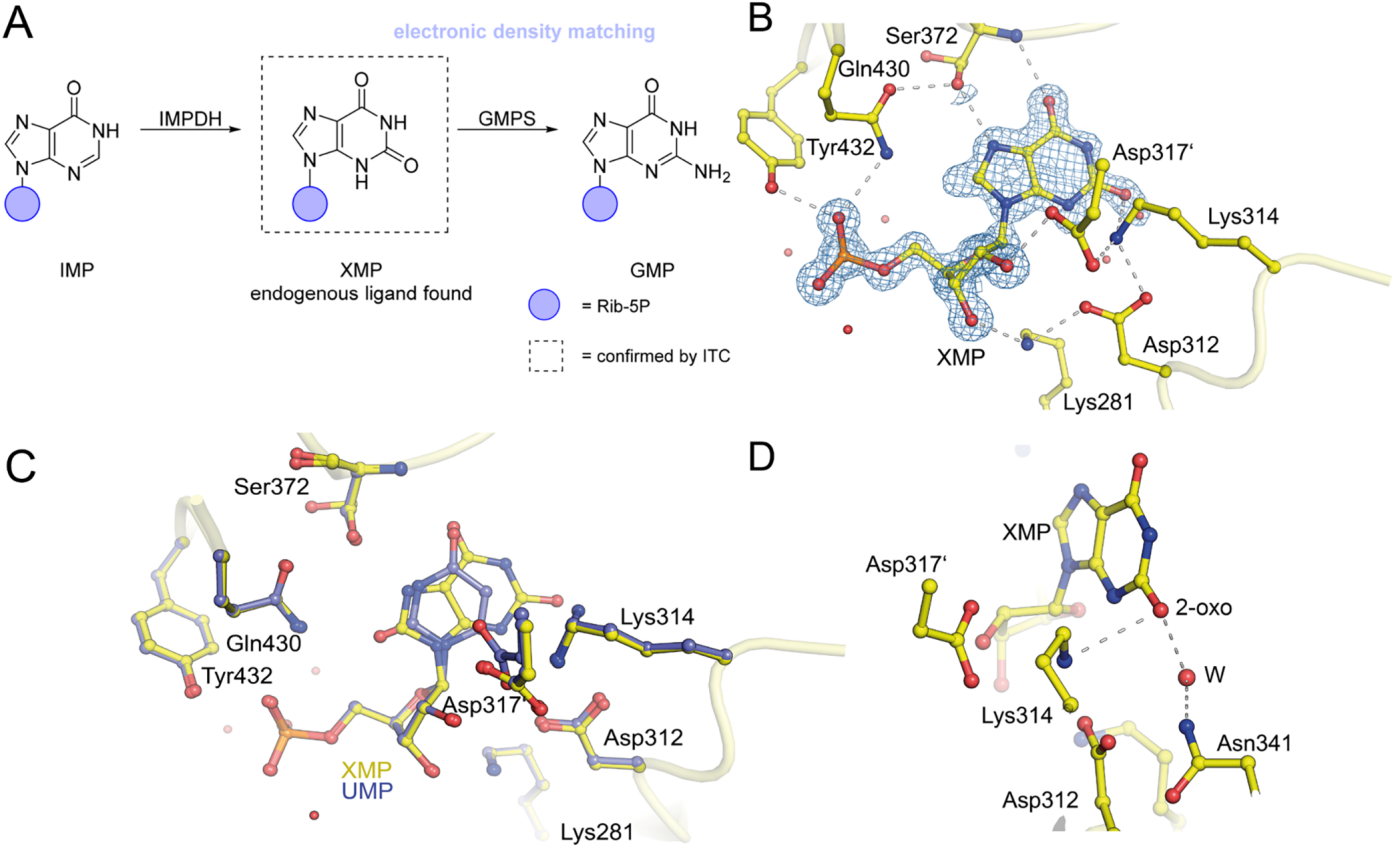
Structure of human OMPDC in complex with XMP. (A) De novo biosynthesis of purines XMP and GMP by IMP dehydrogenase (IMPDH) and GMP synthetase (GMPS). The endogenous ligand XMP bound to human OMPDC was identified through ITC and X-ray crystallography. (B) The crystal structure of XMP bound at the active site of human OMPDC shows a closed, well-ordered enzyme structure at a resolution of 1 Å. XMP is superimposed with the corresponding 2mFo-DFc electron density map, with a contour level of 2σ (PDB: 9HDU). (C) For comparison, the UMP (blue, PDB: 7ASQ) crystal structure was superimposed onto the XMP structure (yellow). The overall binding motif remains similar, with no significant structural changes except for a minor conformational shift of Asp317′. (D) Close-up of the binding motif around the 2-oxo group of XMP with hydrogen bond interactions indicated.

We identified a second endogenous ligand for the OMPDC Thr321Asn variant, which is dTMP (Figure S2). Notably, dTMP lacks a 2′–OH group, unlike the substrate OMP and the product UMP, as it is a deoxy nucleotide. Compared to the wild-type enzyme, ligand binding in the variant displaces the catalytic tetrad from its usual conformation. Using ITC, we estimated the dissociation constant (*K*_D_) for dTMP binding to Thr321Asn as 431 ± 44 nM. Interestingly, the wild-type enzyme can also bind TMP, but with much lower affinity (*K*_D_ = 119 ± 9 μM), which is 16 times weaker than its affinity for UMP.^13^ We then determined the X-ray crystal structure of wild-type OMPDC in complex with dTMP at a resolution of 1.05 Å, as shown in Figure 6. The phosphate gripper and pyrimidine loop surround dTMP, forming the same hydrogen bonds observed with the product UMP. The main differences are observed in the catalytic tetrad, which adopts multiple conformations, indicating high flexibility that may explain the lower binding affinity and possibly results from the absence of the 2′–OH group. Therefore, dTMP generally is not a suitable inhibitor for OMPDC, although the 5-methyl group is well stabilized by van der Waals interactions, as it points directly into the hydrophobic pocket formed by residues Met371, Ile368, and Ile401 (Figure 6B).

**Figure 6:**
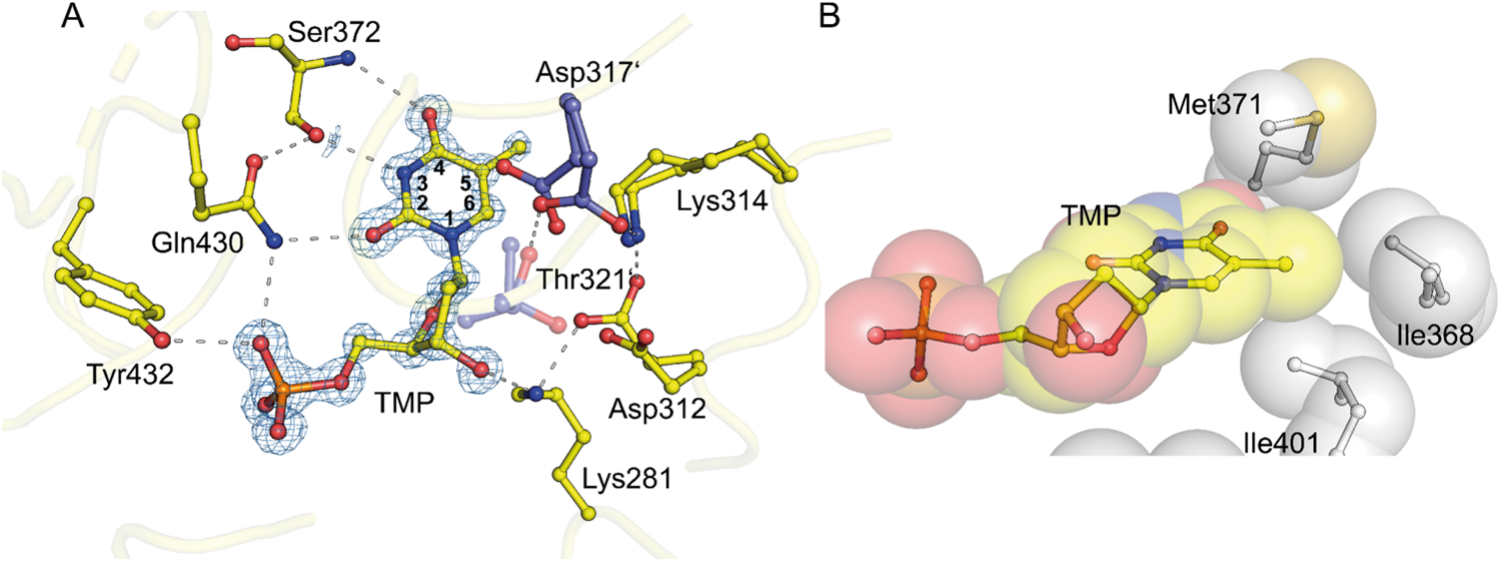
Structure of human OMPDC with dTMP. (A) Active site of human OMPDC with bound dTMP at a resolution of 1.05 Å (PDB: 9HDX). Ligand binding stabilizes the enzyme’s closed conformation, while two major alternate conformations of the catalytic tetrad are visible, highlighting high flexibility. Ligand dTMP is overlaid with the corresponding 2mFo-DFc electron density map at a contour level of 2σ. (B) The 5-methyl group of dTMP is located in a hydrophobic pocket of OMPDC.

To further explore the structural basis of nucleotide binding to OMPDC, we synthesized CMP from cytidine as the starting material. This allowed us to examine how the C4 substituent influences binding, as CMP and UMP are identical except for the functional group at C4 (an oxo group in UMP and an amino group in CMP). Using ITC, the wild-type OMPDC exhibits two phases of CMP binding with apparent dissociation constants of *K*_D1_ = 4.73 ± 1.09 μM and *K*_D2_ = 8.33 ± 1.06 μM (Table 1). These values are pretty similar to those estimated for UMP binding;^13^ however, the crystal structure analysis of human OMPDC in complex with CMP revealed a completely different binding mode (Figure 7A). The nucleotide binds in an unusual *anti*-conformation to the active site, with only partially resolved pyrimidine and phosphate gripper loops, indicating high flexibility. The cytosine base binds to a new pocket, where Gln430 of the phosphate gripper loop is typically located in closed OMPDC structures. Residue Gln430 has been displaced from this site and cannot be traced in the electron density maps. Compared to UMP and similar to the reported structure of MtOMPDC with CMP, the number of hydrogen bonds is significantly reduced (Figure 7B).^42,44^ Despite this, the affinity of human OMPDC for CMP is high, probably reflecting an entropically favorable binding. In conclusion, the C4 oxo group is essential for proper binding of native and artificial nucleotides due to its ability to act as a hydrogen bond acceptor in the interaction with Ser372 of the pyrimidine umbrella.

**Figure 7:**
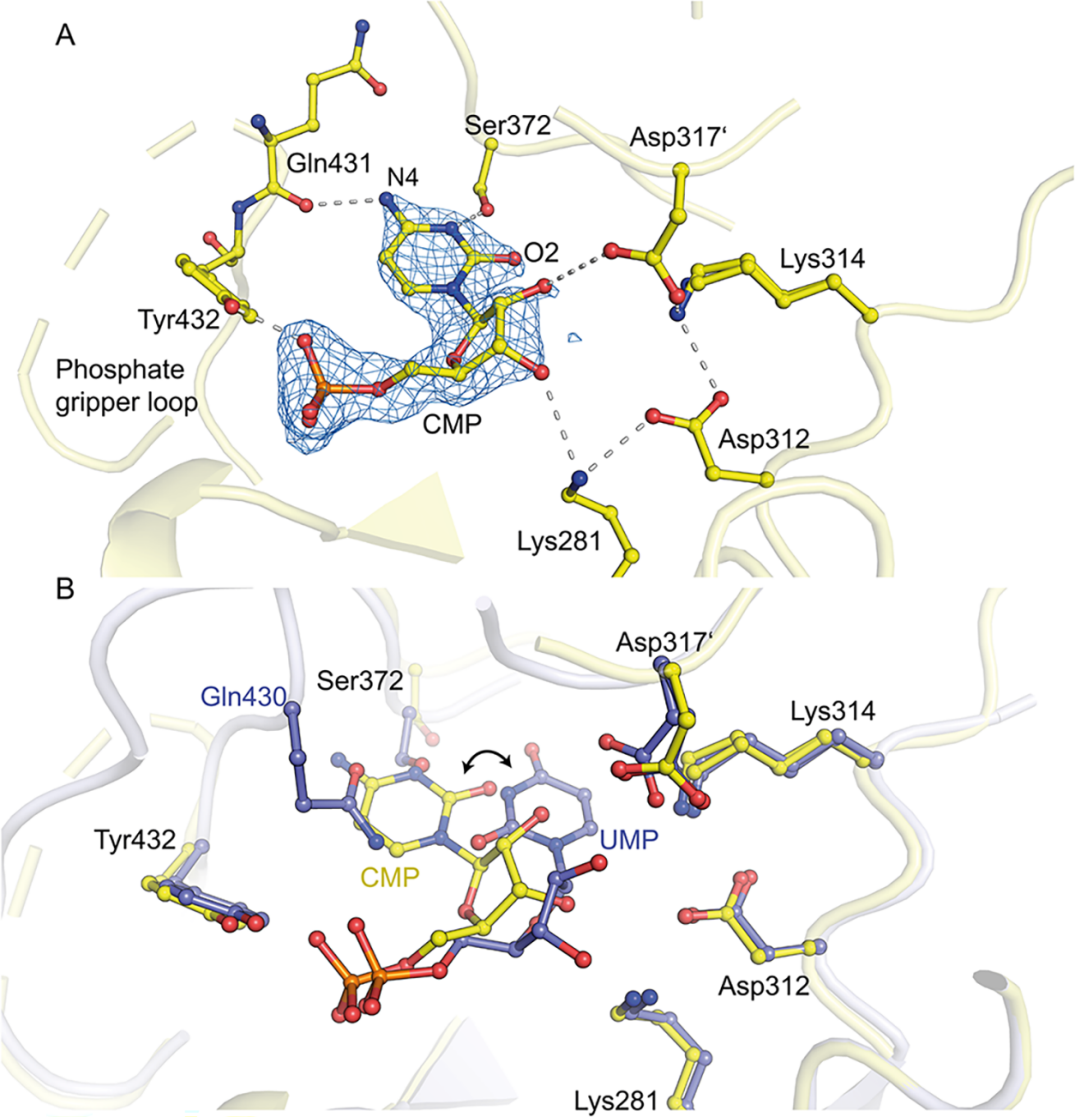
Structure of human OMPDC with CMP. (A) The nucleotide CMP bound to the active site of OMPDC (PDB: 9HIL), showing interacting protein residues and hydrogen bonds. The enzyme conformation is not fully closed, as indicated by the only partially resolved pyrimidine and phosphate gripper loops. CMP is superposed with the corresponding 2mFo-DFc electron density map at a contour level of 1.5σ. (B) For comparison, the structure with bound UMP (in blue) is superimposed on the CMP structure (in yellow). Interestingly, despite the different binding poses of the base moieties, the configuration of the catalytic tetrad remains intact.

### Methyl Group Binding Motif

Given the observed binding of dTMP to human OMPDC, we synthesized 5-methyl OMP to test whether a nucleotide with a hydrophobic group at C5 exhibits improved affinity. Using the standard ITC-based activity assay, we were unable to detect enzymatic turnover of this substrate. This is not surprising as the +I effect of the methyl group further raises the energy of the transition state. We could, however, assess the binding of 5-methyl OMP by thermodynamic ITC analysis and estimated a binding affinity of *K*_D_ = 404 ± 220 nM (Table 1). We wish to note that the estimated *K*_D_ in this case is only a semiquantitative indicator, as subsequent crystal structure analysis revealed that decarboxylation and formation of 5-methyl UMP had occurred in the crystal (Figure 8A). Consequently, the measured heat signal could represent both binding and decarboxylation. In the crystal structure, 5-methyl UMP exhibits a similar binding pose as observed for product UMP (Figure 8B). The most significant difference is observed for Lys314, which is slightly displaced due to the presence of the additional methyl group. However, the configuration of the other catalytically relevant residues Lys281, Asp312, and Asp317′ remains unchanged. Intriguingly, the 5-methyl group binds in a hydrophobic pocket and is sandwiched by hydrophobic residues including Ile368, Ile401, Pro417, and Met371, as observed for dTMP (see above and Figure 6B). Stabilization through additional van der Waals forces has been reported to be effective for other inhibitors.^54,55^ In our case, adding a nonpolar group to YMP or BMP at the C5 position could provide further stabilization and enhanced affinity.

**Figure 8:**
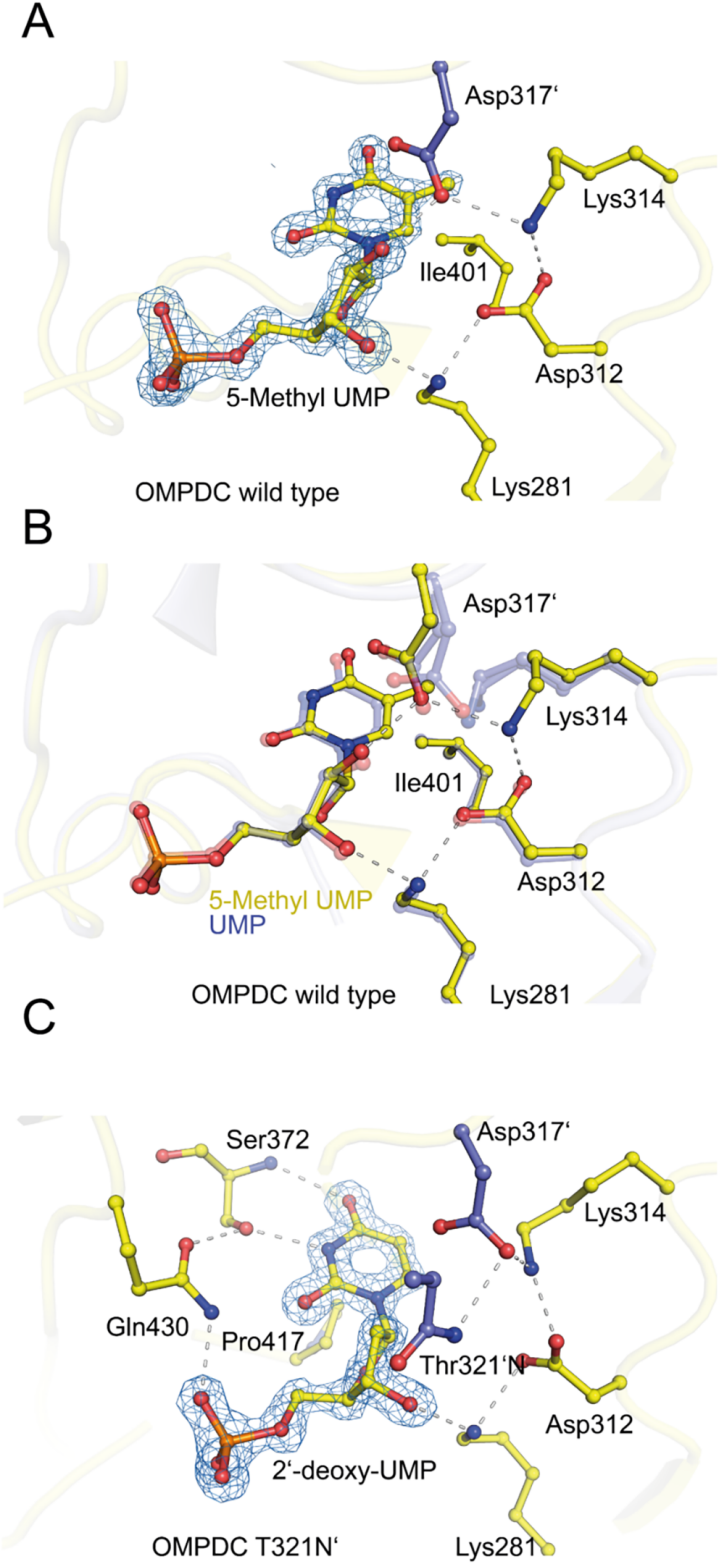
Structure of human OMPDC with 5-methyl UMP and 2′-deoxy UMP. (A) Soaking of 5-methyl OMP into crystals of human OMPDC resulted in the formation of the decarboxylation product 5-methyl UMP (PDB: 9HDZ). The structure of the active site-bound 5-methyl UMP is depicted, highlighting interacting protein residues and hydrogen bond interactions. The corresponding 2mFo-DFc electron density map is shown at a contour level of 3σ. (B) Superposition of active site structure of human OMPDC in complex with UMP structure (in blue) and with 5-methyl UMP (in yellow). (C) Upon soaking of 2′-deoxy OMP into crystals of OMPDC variant Thr321Asn, decarboxylation resulted in the formation of 2-deoxy UMP (resolution at 1.3 Å; PDB: 9HDT). The structure of the active site-bound 2′-deoxy UMP is depicted, highlighting interacting protein residues and hydrogen bond interactions. The corresponding 2mFo-DFc electron density map is shown at a contour level of 2.5σ. The introduced residue Asn321 is part of an extended H-bond network with residues of the catalytic tetrad.

### 2′–OH Binding Motifs

Based on the observation that dTMP binds to human OMPDC, we synthesized 2′-deoxy OMP to obtain more insights into the importance of the ribosyl 2′-hydroxy group for the mechanism of OMPDC. This substrate has been reported to bind effectively to yeast OMPDC.^46,56^ However, in the case of human OMPDC, no binding or turnover was detected for 2′-deoxy OMP and 2′-deoxy UMP, neither in solution nor *in crystallo*. Even when using long soaking times of up to 1 h (tested times 1 min–1 h), neither the substrate nor the decarboxylated product could be traced in the crystal structure. Additionally, thermodynamic ITC experiments revealed no binding of OMPDC wild-type toward 2′-deoxy UMP (Figure S11). Thus, for human OMPDC, the 2′–OH group seems to be essential for substrate binding and catalysis. Interestingly, human OMPDC can bind dTMP as discussed above. This suggests that the methyl group at C5 may compensate for the loss of binding energy provided by the 2′–OH group.

These observations notwithstanding, we were able to detect binding and turnover of 2′-deoxy OMP in the variant Thr321Asn, which exhibits high affinity for dTMP (vide supra). The enzymatic activity with this substrate was too low to be reliably measured by kinetic ITC. However, soaking of 2′-deoxy OMP into Thr321Asn crystals allowed us to determine the structure of human OMPDC in complex with the decarboxylated product that is 2′-deoxy UMP (Figure 8C). The active site exhibits a distinct structure compared to the canonical one, characterized by marked displacements of the catalytic tetrad, yet retains a fully ordered phosphate gripper and pyrimidine umbrella loop regions. Consequently, the introduced asparagine in the variant alters the substrate binding motif, enabling the 2′-deoxy substrate to bind more effectively. This demonstrates the importance of Thr321 for recognition and binding of the substrate 2′–OH group in human OMPDC.

As the 2′–OH group of the substrate is essential for binding, we sought to explore this interaction further. Specifically, the 2′–OH group interacts with residues Thr321 and the catalytically relevant Asp317, both of which are contributed by the second monomer. Thus, we explored these interactions by substituting the 2′–OH group with a larger thiol group that is less electronegative but more nucleophilic. As we were unable to purify this analog to homogeneity, we were unfortunately unable to test binding and enzymatic turnover. However, we were able to solve the crystal structure of human OMPDC in complex with it. The ligand 2′-SH UMP binds in the active site in a defined manner, and its 2′-thiol group is well resolved due to the strong electron density (Figure 9A). Compared to the structure of OMPDC with UMP, the thiol group displaces residue Asp317′ by ∼2 Å, while the remaining residues of the catalytic tetrad retain their positions. Thus, the interaction of the 2′-SH group with Asp317′ is lost, and a new hydrogen bond between Lys314 and 2′-SH is formed with an interatomic distance of ∼3.2 Å. Akin to UMP’s 2′–OH group, the 2′-SH still interacts with Thr321′ from the second monomer. Based on the crystal structure, we speculate that, although sulfur is a better nucleophile, its size prevents proper binding because it displaces Asp317′ from the active site. This finding further supports our previous observations^13^ that substrate binding in OMPDC involves not only direct interactions between the ligand and protein residues in the active site but also an extended hydrogen bond network between the two monomers, which is sensitive even to minor changes. Additionally, residue Asp317′ has been proposed to be critically involved in a communication pathway connecting both active sites of the two monomers. As discussed above for dTMP and 2′-deoxy OMP, having one fewer interaction, especially from the 2′–OH group to Asp317′ of the second monomer, could lead to lower affinity or prevent binding and catalysis entirely.

**Figure 9:**
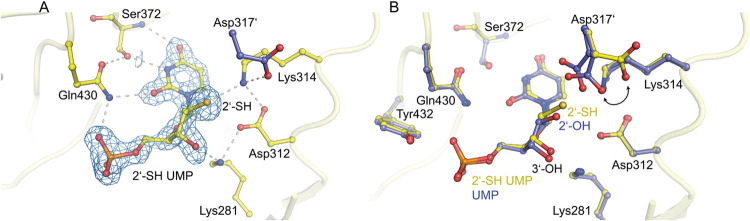
Structure of human OMPDC with 2′-SH UMP. (A) Crystal structure of OMPDC with 2′-SH UMP bound to the active site (PDB: 9HDY). The structure of the active site-bound analog is shown, highlighting interacting protein residues and hydrogen bond interactions. The ligand structure is superimposed with the corresponding 2mFo-DFc electron density map at a contour level of 1.5σ. (B) Superimposition of the active site structures of human OMPDC with UMP (in blue) and 2′-SH UMP (in yellow). Note that the thiol group of 2′-SH UMP displaces Asp317′ from the active site, leading to an altered configuration of the catalytic tetrad.

## Conclusion

In this study, we have reported various high-resolution X-ray crystal structures and binding affinities of human OMPDC for transition-state and substrate/product analogs featuring different purine and pyrimidine bases, as well as 2′-ribose-modified analogs. Analysis of the newly afforded transition-state analog YMP head-to-head with BMP revealed similar Gibbs energies of binding for both ligands. YMP exhibits a higher enthalpy of binding compared to BMP; however, a 1.72 kcal/mol difference was observed in the entropic values corresponding to the additional hydrogen bonds between a water molecule and YMP, as seen in the crystal structure. This enthalpy–entropy compensation prevented an increase in affinity for YMP relative to BMP. The addition of a nonpolar group at C5 could, in principle, prevent a H-bond formation with a water molecule, and thus provide further stabilization. This assumption was corroborated by analysis of the analog 5-Me OMP, which binds to OMPDC with nanomolar affinity, whereas the natural substrate exhibits an *S*_0.5_ of 30 μM. Additionally, no water molecules were observed near the hydrophobic pocket surrounding C5. A substitution with fluorine at C5 (F-UMP) further confirms the displacement of water molecules from the hydrophobic pocket. However, the electronegative atom might be too small for the pocket, which could weaken the overall binding.^28^

From the unexpected binding of the endogenous ligands XMP and dTMP, new interactions within the ligand-enzyme complex were examined, including the oxo groups, the −CH_3_ moiety at C5, and the ribosyl 2′–OH group. XMP is a strong binder of human OMPDC, unlike the related GMP, which did not bind at all. As XMP is the penultimate intermediate in the de novo biosynthesis of purines, this might indicate a regulatory crosstalk between the pyrimidine and purine biosynthetic pathways. For CMP, binding was observed; however, in the crystal structure, the nucleotide was seen in the *anti*-conformation with high flexibility and incomplete loop regions. Therefore, for both purine and pyrimidine nucleobases, the oxo groups were identified as essential anchors for enabling proper binding and maintaining a defined conformation.

Furthermore, we explored the 2′–OH binding motif to residues Thr321′ and Asp317′ using the 2′-deoxy analog of OMP and 2′-SH UMP. Crystal structure analysis revealed that 2′-deoxy OMP does not bind to human OMPDC wild-type. Independent ITC experiments supported this. Nonetheless, we obtained a structure of the OMPDC variant Thr321Asn in complex with 2′-deoxy UMP after soaking with the substrate 2′-deoxy OMP. The endogenous ligand dTMP binds with high, nanomolar affinity to the Thr321Asn variant but also with 10∧3 lower affinity to the wild-type enzyme. This supports our hypothesis that the additional stabilization comes from the methyl group at C5, as previously stated for 5-Me OMP. Structure analysis of human OMPDC with 2′-SH UMP revealed that a larger atom can fit into the active site, but it requires Asp317 to be displaced. Therefore, the interaction between the substrate 2′–OH group and the enzyme appears to be essential for proper substrate binding in human OMPDC.

Overall, the binding site of human OMPDC is flexible enough to accommodate various ligands (Figure 10). This opens the possibility for a new generation of OMPDC inhibitors, starting from the YMP or BMP scaffold, with modifications only at the nucleobase. Based on our studies, we suggest that these next-generation inhibitors should contain functional groups at C5 that enable interactions with the hydrophobic pocket in the active site, as shown in Figure 10. This could be, for instance, methyl or ethyl groups, similar to the characterized analogs 5-Me OMP and dTMP (*vide supra*), as reported for other drug candidates.^57^ For further exploration, the hydrogen atoms in these groups could be replaced with larger atoms, such as halogens (e.g., CCl_3_) or deuterated methyl groups.^28,58^ We believe that these modifications could lead to the discovery of potent OMPDC inhibitors, which could be used to selectively target cancer, West Nile virus, malaria, and Alzheimer’s disease, showing improved affinities and opening new possibilities for chemical probes and drug development.

**Figure 10:**
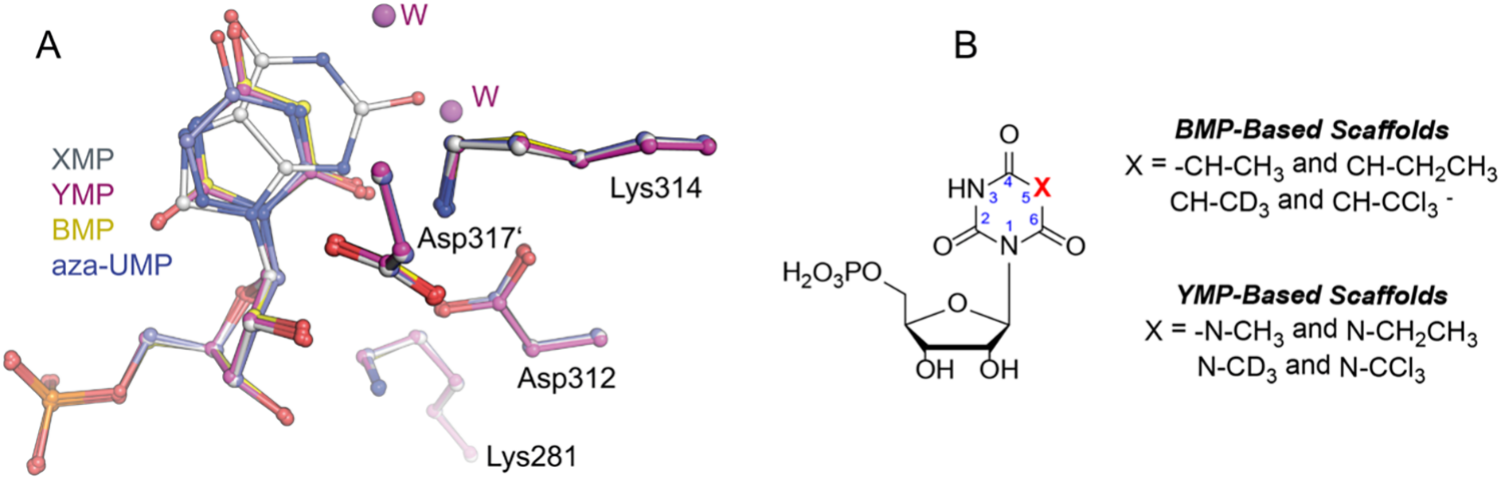
Binding poses of ligands in human OMPDC. (A) Superimposed active site structures of human OMPDC bound to high-affinity ligands XMP (gray), YMP (pink), BMP (yellow), and aza-UMP (blue). (B) Chemical structures of new potential inhibitors based on the BMP or YMP scaffolds.

Finally, our results are consistent with the notion that many different conformations, in addition to the one observed for the Michaelis complex, are possible for OMPDC, and that this might expand the scope for tight-binding inhibition as reported by others.^59^

## Supplementary Material



## Notes

Additional experimental details, including materials, methods, NMR spectra for all compounds, and a Table detailing the crystallographic statistics (PDF)

## Abbreviations

OMPD: orotidine 5′-monophosphate decarboxylase
OMP: orotidine 5′-monophosphate
UMP: uridine 5′-monophosphate
BMP: 6-hydroxyuridine 5′-phosphate
aza-UMP: 6-aza-uridine 5′-monophosphate
CMP: cytidine 5′-monophosphate
YMP: 1-(β-d-ribofuranosyl) cyanuric acid 5′-monophosphate
DeoxyUMP: 2′-deoxy uridine 5′-monophosphate
GMP: guanosine 5′-monophosphate
2′-SH UMP: 2′-thio uridine 5′-monophosphate
dTMP: (2-deoxy)­thymidine 5′-monophosphate
XMP: xanthosine 5′-monophosphate
