# Supplementary material for: Expected and Unexpected “Guests” at the Active Site of Human Orotidine 5′-Monophosphate Decarboxylase
Source: Biochemistry. 2025 Oct 8;64(22):4542–54. doi: 10.1021/acs.biochem.5c00459 (PMC12631982; doi:10.1021/acs.biochem.5c00459)
Supplement: Supplementary file 1 [file bi5c00459_si_001.pdf]

Supporting Information

Expected and unexpected "guests" at the active site of human orotidine 5'-monophosphate decarboxylase

Laura Liliana Kirck [1,2 #], Elisa Santagostino [3,4 #], Laurin Brandhoff [3,4], Nadja A. Simeth [3,4 *], and Kai Tittmann [1,2 *]

[1] Department of Molecular Enzymology, Göttingen Center of Molecular Biosciences and Albrecht-von-Haller Institute, Georg-August University Göttingen, Julia-Lermontowa-Weg 3, D-37077 Göttingen, Germany

[2] Max-Planck-Institute for Multidisciplinary Sciences, Am Fassberg 11, D-37077 Göttingen, Germany

[3] Institute for Organic and Biomolecular Chemistry, Georg-August University Göttingen, Tammannstr. 2, D-37077 Göttingen, Germany

[4] Cluster of Excellence "Multiscale Bioimaging: from Molecular Machines to Networks of Excitable Cells" (MBExC), University of Göttingen, D-37075 Göttingen, Germany

[#] These authors contributed equally.

* To whom correspondence shall be addressed: nadja.simeth@uni-goettingen.de or ktittma@gwdg.de

Table of Contents

1. Supplementary Figures S3
2. Materials & Methods S4
3. Chemical synthesis of analogues S6
a. Transition-state analogues BMP and YMP S6
3.1.1. Synthesis of 1-(β-D-ribofuranosyl)-barbituric acid-5'-monophosphate (BMP) S6
3.1.2. Synthesis of 1-(β-D-ribofuranosyl)cyanuric acid-5'-monophosphate (YMP) S8
b. Synthesis of Cytidine-5’-monophosphate (CMP) (adapted from [4]) S9
c. Synthesis of 5-Methylorotidine-5’-monophosphate (5-Methyl OMP) (adapted from [1]) S10
d. Synthesis of 2’-Deoxy-oritidine-5’-monophosphate (Deoxy OMP) (adapted from [1]) S12
e. Deoxyuridine-5’-monophosphate (Deoxy UMP) (adapted from [4]) S13
f. Synthesis of 2’-SH UMP S14
4. Isothermal Calorimetry S17
5. NMR Spectra S20
6. Table Crystallographic statistics S48
7. References S50

1. Supplementary Figures

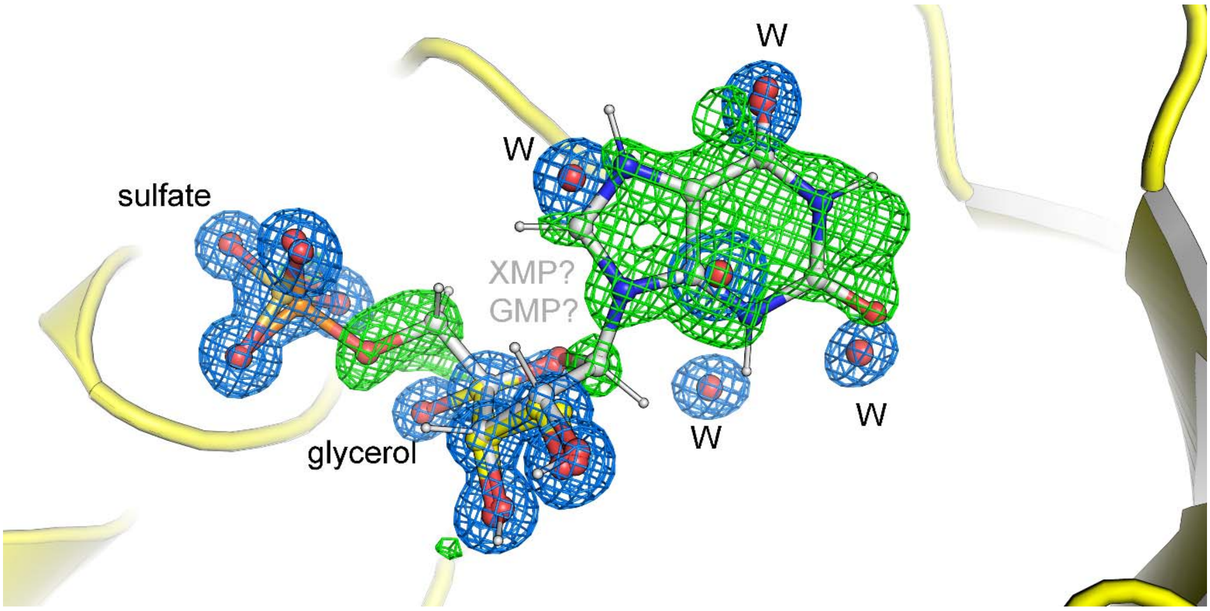

Figure S1. Indications for an endogenous ligand bound to the active site of human OMPDC. Crystal structure of human OMPDC in the as-isolated apo form (PDB ID 7OV0).[1] The bound ligands, sulfate, glycerol, and solvent molecules (W) are shown with the corresponding 2mFo-DFc electron density map contoured at 4σ (in blue). Note the presence of weak, positive difference electron density in the mFo-DFc map (contour level 3σ, in green) that indicates the presence of a purine nucleotide at low occupancy. The shape of the density suggests that XMP (model shown in grey) or GMP is a potential binder (see main text).

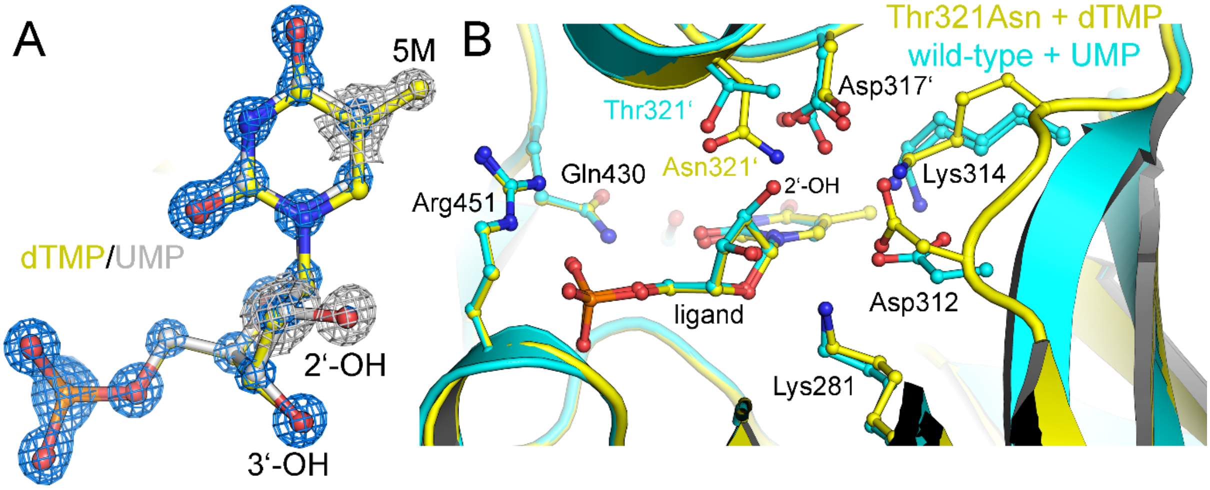

Figure S2. Indications for an endogenous ligand bound to the active site of human OMPDC variant Thr321Asn. The 2mFo-DFc electron density map, shown at contour levels of 2σ (in grey) and 4σ (in blue), suggests the co-presence of a ligand with a 5-methyl group (dTMP) and a second ligand with a 2'-OH group but without a 5-methyl group (UMP). The observed electron density map is not compatible with the exclusive presence of either UMP, dUMP or dTMP. Competitive refinements suggest the co-presence of UMP and dTMP with ~50% occupancy each.

2. Materials & Methods

General Information about Reagents and Solvents

All reagents, including anhydrous solvents, were purchased from commercial suppliers (ABCR, Acros Organics, Alfa Aesar, Fisher Scientific, Merck, Roth, Sigma Aldrich, VWR, and TCI), and used as received. Trimethyl phosphate was dried over 3 Å molecular sieves under an argon atmosphere, and $POCl_3$ was freshly distilled for the synthesis of **BMP**. Ultra-pure water was obtained by purifying demineralized water using an Arium mini water purification system from Sartorius.

Moisture-sensitive reactions

All air or moisture-sensitive reactions were performed using the standard Schlenk technique under an argon atmosphere.

Freeze-Drying

Nucleotide solutions were frozen in liquid nitrogen, and the water was sublimated under reduced pressure (<1 mbar) using an Alpha-2-4-LD plus freeze-dryer from Martin Christ Gefriertrocknungsanlagen GmbH.

Chromatography

Analytical thin-layer chromatography (TLC) was performed on silica gel 60 F254 plates pre-coated with fluorescent indicator from Merck. The spots were visualized by UV light (254 nm). For the purification of crude products, column chromatography was performed using Merck Geduran silica gel 60 (0.040–0.063 mm particle size; 230–450 mesh) either on a Biotage Isolera One or by manual flash column chromatography with solvents of technical grade. The samples were loaded on the column as concentrated solutions or as a silica pad. The technique, conditions, eluent mixtures, and gradients are specified for each synthetic procedure in the experimental section.

High-Performance Liquid Chromatography (HPLC)

HPLC purification was performed using devices from JASCO Deutschland GmbH. Preparative purification was performed using two devices: one equipped with PU-4086 pumps, a UV-4075 detector, and a CO-4060 column oven, the second one with two pumps PU-2020Plus, a 3-line degasser DG2080-53 and a diode array detector MD-2010Plus. An anion exchange column (Spherisorb SAX OBD 80A 5) was used for the purification with a flow rate of 10 mL/min. Substances were eluted with a linear gradient using ultra-pure water (A) and 0.5 M triethylammonium acetate (TEAA) buffer at pH = 7 (B) as eluents with a gradient of 1 to 99% solvent B in 15 min. Nucleotides were detected by UV light (265 nm). The samples were prepared in ultra-pure water and then filtered through a Chromafil RC 45/15 MS filter from Macherey-Nagel.

Nuclear Magnetic Resonance (NMR)

^{1}H NMR spectra were recorded on Avance III HD (300, 400, or 500 MHz) devices from Bruker at 25°C. ^{13}C NMR spectra were recorded on the same devices at the corresponding frequencies of 75, 101, and 126 MHz. ^{31}P NMR were recorded at the frequencies of 121 and 162 MHz on 300 MHz and 400 MHz Avance III HD devices, respectively, at 25°C. ^{19}F NMR spectra were recorded at the frequency of 377 MHz on a 400 MHz Avance III HD device. The deuterated solvents used and measurement frequencies are specified for each compound individually in the experimental section. The solvent signal was used as an internal reference: $CDCl_3$ (7.26 ppm), DMSO (2.50 ppm), and D_2O (4.79 ppm) for ^{1}H NMR spectra; and $CDCl_3$ (77.2 ppm) and DMSO (39.5 ppm) for ^{13}C NMR spectra. Chemical shifts δ are shown in ppm and coupling constants *J* in Hz. For the characterisation, the following abbreviations were used: s = singlet, sbr = broad singlet, d = doublet, dd = doublet of doublets, t = triplet, dt = doublet

of triplets, m = multiplet. The evaluation of the NMR spectra was done with MestReNova 14.2.0-26256 from Mestrelab Research S.L.

Mass Spectrometry (MS)

Electrospray ionization (ESI) and high-resolution ESI (HR-ESI) spectra were recorded using a microTOF-Q II or a maXis ESI-QTOF-MS instrument from Bruker. The values are given as m/z relation.

Infrared spectroscopy (IR)

Infrared spectroscopy spectra were measured on an Alpha-P ATR of Bruker. Solid and oil samples were placed as pure substances. The range of 4000 cm^{-1} – 400 cm^{-1} was recorded. The spectra were evaluated with Bruker's program Opus 6.5.

Melting Point (m.p.)

The melting points were measured with a Stuart Melting Point Apparatus SMP3 from Barloworld Scientific and an MP-200 Series StuartTM Digital Melting Point Apparatus from Cole-Parmer®.

3. Chemical synthesis of analogues

a. Transition-state analogues BMP and YMP

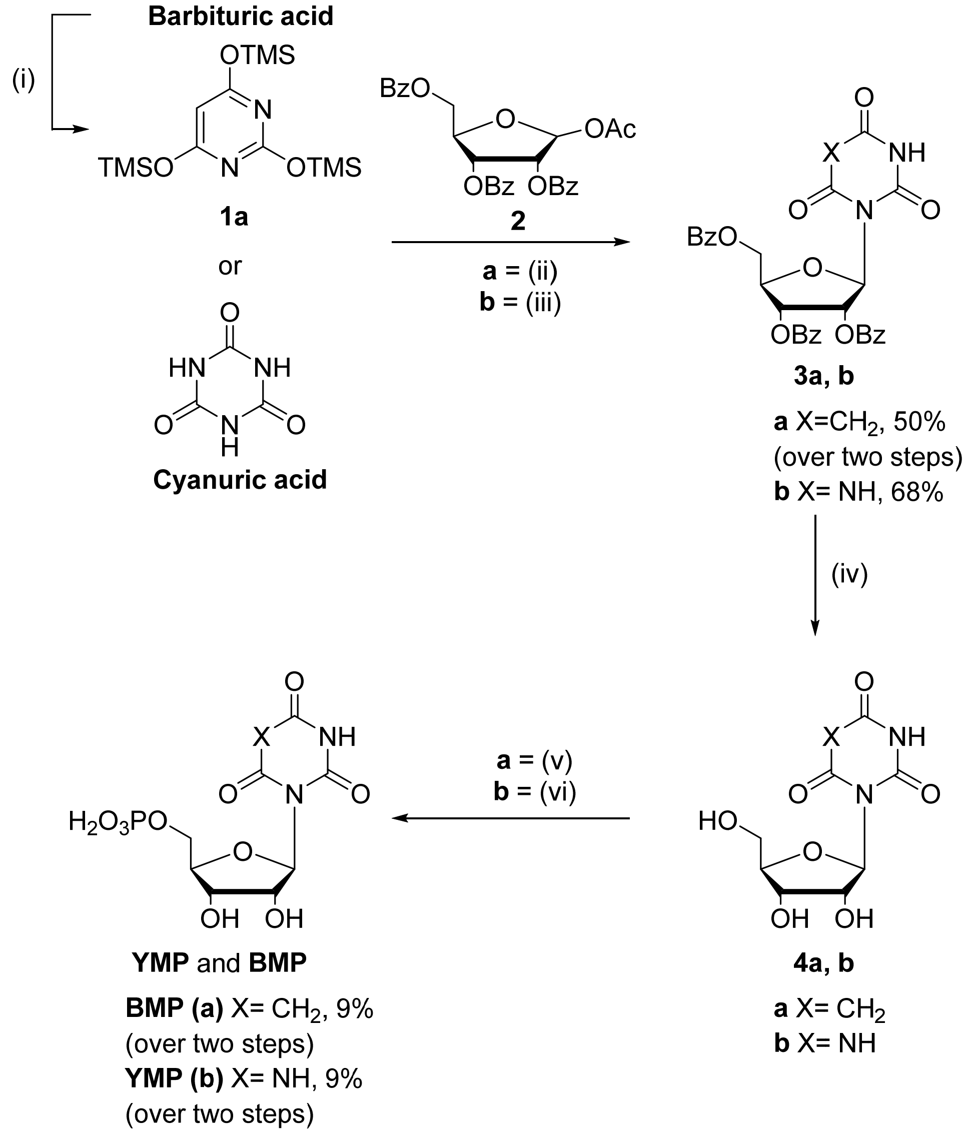

Figure S3. Synthesis route towards transition state analogues BMP and YMP. Reagents and conditions: (i) TMSCl, HMDS, 16 h, reflux; (ii) $SnCl_4$, DCE, 6 h, rt, 50%; (iii) TMSCl, HMDS, $SnCl_4$, MeCN, 1 h, rt, 68%; (iv) 1 M NaOH in MeOH, 1 h, rt; (v) $POCl_3$, $(PO(OCH_3)_3$, 6 h, 0°C, 9%; (vi) $POCl_3$, $(PO(OCH_3)_3$, H_2O, 16 h, 0°C, 9%.

3.1.1. Synthesis of 1-(β-D-ribofuranosyl)-barbituric acid-5'-monophosphate (BMP)

2,4,6-Tris(trimethylsilyloxy)pyrimidine (1a) [1]

Barbituric acid (1.28 g, 10 mmol, 1 eq.) was dissolved in HMDS (30 mL). TMSCl (1 mL) was added, and the reaction mixture was heated to reflux for 6 h. The resulting solution was cooled to room temperature, and the solvent was removed in *vacuo*. The product was obtained as a light-yellow solid (mixture weight: 3.46 g) in a mixture with barbituric acid (1:1.5 product:barbituric acid). It was used in the next step without further purification. **^{1}H NMR** (300 MHz, DMSO) δ 5.28 (s, 1H), 0.03 (dd, J = 14.1, 0.8 Hz, 27H). Barbituric acid signals at 11.07 and 3.48 ppm.

2',3',5'-Tri-*O*-benzoyl-6-hydroxyuridine (3a) (adapted from [1])

To a solution of 2,4,6-tris(trimethylsilyloxy)pyrimidine (1.13 g, 2.15 mmol of 2,4,6-tris(trimethylsilyloxy)pyrimidine, 1.0 eq., **1a**) in 1,2-dichloroethane (15 mL), $SnCl_4$ (337 µL, 0.77 g, 3.72 mmol, 1.25 eq.) was added under argon atmosphere. Then, a solution of 1-*O*-acetyl-2,3,5-tri-*O*-benzoyl-β-D-ribofuranose (1.50 g, 2.97 mmol, 1.38 eq., **2**) in 1,2-dichloroethane (8 mL) was added slowly. The resulting reaction mixture was stirred for 6 h at room temperature and poured into a sat. aq. $NaHCO_3$ solution (50 mL). The suspension was filtered through a pad of silica and the layers were separated. The organic layer was dried over $MgSO_4$, and the solvent was removed under reduced pressure. The crude product was purified by column chromatography (DCM/MeOH, 9:1). The product was obtained as a light-yellow oil (620 mg, 1.08 mmol, 50%). **R_f**= 0.53 (DCM/MeOH, 9:1). **^{1}H NMR** (400 MHz, DMSO) δ 7.98 (dt, J = 8.4, 1.4 Hz, 2H), 7.95 – 7.90 (m, 2H), 7.81 – 7.74 (m, 2H), 7.69 – 7.53 (m, 3H), 7.46 (td, J = 7.6, 5.1 Hz, 4H), 7.33 (t, J = 7.8 Hz, 2H), 6.48 (s, 1H), 6.15 – 6.05 (m, 2H), 4.62 (ddd, J = 12.7, 8.9, 3.4 Hz, 2H), 4.55 – 4.45 (m, 1H). **^{13}C NMR** (126 MHz, DMSO) δ 165.5, 164.8, 164.6, 151.5, 133.9, 133.7, 133.7, 133.4, 129.4, 129.3, 129.2, 128.8, 128.7, 128.7, 128.6, 128.6, 84.9, 77.6, 74.2, 70.5, 63.7. **HRMS** (ESI): m/z calculated for $C_{30}H_{24}N_2O_{10}$ $[M-H]^-$: 571.1358, found: 571.1363; m/z calculated for $C_{30}H_{24}N_2O_{10}$ $[M+Na]^+$: 595.1323, found: 595.1318; m/z calculated for $C_{30}H_{24}N_2O_{10}$ $[M+2Na]^+$: 617.1143, found: 617.1147. CH_2 protons of the barbituric acid in position 5 as well as the carbon signal were not observed in the NMR analysis. HR-MS data along with the crystal structure of the final compound **BMP** confirmed the synthesis of 2',3',5'-tri-*O*-benzoyl-6-hydroxyuridine.

6-Hydroxyuridine (4a)

2',3',5'-Tri-*O*-benzoyl-6-hydroxyuridine (609 mg, 1.06 mmol, 1.0 eq., **3a**) was dissolved in a solution of 1 M NaOH in MeOH (4.26 mL, 4.26 mmol, 4 eq.), and the resulting mixture was stirred at room temperature for 2 h. The reaction was quenched by 10% HCl (1.40 mL) till pH 3 was obtained. Then, the solution was diluted with water and chloroform (10 mL each), and the layers were separated. The aqueous phase was freeze-dried to afford a white solid (407 mg). The product was used in the next step without further purification. **^{1}H NMR** (400 MHz, D_2O) δ 6.28 (d, J = 3.9 Hz, 1H), 4.84 (dd, J = 6.3, 4.0 Hz, 1H), 4.47 (t, J = 6.6 Hz, 1H), 4.06 (td, J = 6.3, 2.7 Hz, 1H), 3.96 (dd, J = 12.4, 2.9 Hz, 1H), 3.82 (dd, J = 12.4, 5.9 Hz, 1H). **HRMS** (ESI): m/z calculated for $C_9H_{12}N_2O_7$ $[M-H]^-$ 259.0572, found: 259.0571; m/z calculated for $C_9H_{12}N_2O_7$ $[M+Na]^+$ 283.0537, found: 283.0537. CH_2 protons of the barbituric acid in position 5 as well as the carbon signal were not observed in the NMR analysis. HR-MS data along with the crystal structure of the final compound **BMP** confirmed the synthesis of 6-hydroxyuridine.

1-(β-D-Ribofuranosyl)barbituric acid-5'-monophosphate (**BMP**) (adapted from [1])

In a flame-dried flask, 6-hydroxyuridine (407 mg, 1.56 mmol, 1.0 eq., 4a) was dissolved in dry trimethyl phosphate (5.53 mL) under an argon atmosphere, and the solution was cooled to 0°C. Freshly distilled $POCl_3$ (292 µL, 3.13 mmol, 2.0 eq.) was added dropwise, and the resulting solution was stirred at 0°C for 6 h. The reaction mixture was poured into ice-cold water (50 mL) and left to stir for a further 30 min. The solution was neutralised with sat. $NaHCO_3$ aq. (13 mL) and the solvent was removed under reduced pressure. The crude product was purified by automated flash chromatography (20% to 40% 2-propanol in NH_4OH-water (3:2),). The desired product was obtained as diammonium salt (34.9 mg, 93.3 µmol, 9% over two steps). **^{1}H NMR** (400 MHz, D_2O) δ 6.17 (d, J = 3.1 Hz, 1H), 4.71 (dd, J = 6.4, 3.1 Hz, 1H), 4.42 (dd, J = 7.8, 6.4 Hz, 1H), 4.08 (ddd, J = 11.0, 5.6, 3.2 Hz, 1H), 4.00 (ddd, J = 7.7, 6.5, 3.1 Hz, 1H), 3.92 (dt, J = 11.0, 6.2 Hz, 1H). [3.52 (d, 0.39 H, $(CH_3O)_3PO$)), 1.99 (s, 1H, impurity)]. **^{13}C NMR** (101 MHz, D_2O) δ 166.7, 153.0, 87.4, 81.3 (d, $J_{C,P}$ = 8.3 Hz), 71.6, 69.5, 64.5(d, $J_{C,P}$ = 4.7 Hz). **^{31}P NMR** (162 MHz, D_2O) δ 1.96. **HRMS** (ESI): m/z calculated for $C_9H_{13}N_2O_{10}P$ $[M-H]^-$ 339.0235, found: 339.0230; m/z calculated for $C_9H_{13}N_2O_{10}P$ $[M+H]^+$ 341.0381, found: 341.0372. CH_2 protons of

the barbituric acid in position 5, as well as the carbon signal, were not observed in the NMR analysis. HR-MS data, along with the crystal structure, confirmed the synthesis of **BMP**.

3.1.2. Synthesis of 1-(β-D-ribofuranosyl)-cyanuric acid-5'-monophosphate (YMP)

β-D-Ribofuranose-1-cyanuryl-2,3,5-tribenzoate (3b) [2]

Cyanuric acid (2.30 g, 18 mmol, 3 eq.) and 1-*O*-acetyl-2,3,5-tri-*O*-benzoyl-β-D-ribofuranose (3.00 g, 6 mmol, 1 eq., **2**) were dissolved in MeCN (50 mL) under an argon atmosphere. Hexamethyldisilane (HMDS) (3.76 mL, 18 mmol, 3 eq.), trimethylsilyl (TMSCl) (0.80 mL, 6 mmol, 1 eq.), and $SnCl_4$ (1.20 mL, 10 mmol, 2 eq.) were added. The mixture was stirred at room temperature for 1 h. MeOH was added, and the solution was centrifuged. The supernatant was separated, and the volatiles of the supernatant were removed under reduced pressure. The crude product was purified by column chromatography (toluene/EtOAc, 4:1). The final product was obtained as a colorless solid (2.33 g, 4.07 mmol, 68%). $\mathbf{R_f}$= 0.24 (toluene/EtOAc, 4:1). **^{1}H NMR** (400 MHz, DMSO) δ 11.77 (s, 2H), 8.00 (m, 2H), 7.96 (m, 2H),7.81 – 7.73 (m, 2H), 7.74 – 7.54 (m, 3H), 7.53 – 7.42 (m, 4H), 7.39 – 7.30 (m, 2H), 6.38 (d, J = 2.0 Hz, 1H), 6.13 – 6.03 (m, 2H), 4.71 – 4.58 (m, 2H), 4.51 (dd, J = 11.9, 5.2 Hz, 1H). **^{13}C NMR** (101 MHz, DMSO-*d6*) δ 165.5, 164.7, 164.5, 149.1, 148.3, 133.9, 133.7, 133.5, 129.4, 129.3, 129.2, 129.2, 128.8, 128.7, 128.7, 128.5, 128.5, 85.6, 77.8, 73.7, 69.9, 63.3. **HRMS** (ESI): m/z calculated for $C_{29}H_{23}N_3O_{10}$ $[M+H]^+$: 574.1456, found: 574.1454; m/z calculated for $C_{29}H_{23}N_3O_{10}$ $[M+Na]^+$: 596.1276, found: 596.1275; m/z calculated for $C_{29}H_{23}N_3O_{10}$ $[M+NH_4]^+$: 591.1722, found: 591.1721.

β-Cyanuryl-Ribose (4b) [2]

β-D-ribofuranose 1-cyanuryl 2,3,5-tribenzoate (0.50 g, 0.87 mmol, 1 eq., **3b**) was dissolved in a solution of 1 M NaOH in MeOH (3.49 mL, 4 eq.), and the reaction mixture was stirred at room temperature for 1 h. The reaction was acidified with 10% HCl (1.2 mL) to a pH of 3. The solution was diluted with water and chloroform (5 mL each), and the layers were separated. The aqueous phase was freeze-dried to afford a colourless solid (0.33 g). The product was used in the next step without further purification. **^{1}H NMR** (300 MHz, D_2O) δ 6.09 (d, *J* = 3.4 Hz, 1H), 4.73 (dd, *J* = 6.2, 3.4 Hz, 1H), 4.40 (t, *J* = 6.6 Hz, 1H), 3.98 (td, *J* = 6.6, 3.0 Hz, 1H), 3.89 (dd, *J* = 12.3, 3.0 Hz, 1H), 3.74 (dd, *J* = 12.4, 6.2 Hz, 1H). **HRMS** (ESI): m/z calculated for $C_8H_{11}N_3O_7$ $[M-H]^-$: 260.0524, found: 260.0526; m/z calculated for $C_8H_{11}N_3O_7$ $[M+Na]^+$: 284.0489, found: 284.0493.

1-(β-D-Ribofuranosyl)cyanuric acid-5'-monophosphate (YMP) (adapted from [3])

To a solution of $POCl_3$ (73 µL, 0.77 mmol, 2 eq.) in trimethyl phosphate (1 mL) cooled to 0°C, β-cyanuryl-ribose (0.10 g, 0.40 mmol, 1 eq., **4b**) and water (2 µL, 0.13 mmol, 0.3 eq.) were added. The mixture was kept at 0°C and stirred for 16 h. The solvent was removed under reduced pressure and the crude product was purified by automated flash chromatography (2% to 40% 2-propanol in NH_4OH-water (3:2)). The desired product was obtained as a diammonium salt (8.7 mg, 23.1 µmol, 9% over two steps, based on the 0.33 g obtained in the previous step). **^{1}H NMR** (300 MHz, D_2O) δ 6.08 (d, *J* = 3.2 Hz, 1H), 4.78 – 4.69 (m, 1H), 4.44 (t, *J* = 6.6 Hz, 1H), 4.14 – 4.02 (m, 2H), 3.96 (dt, *J* = 11.2, 5.7 Hz, 1H). [2.00 (d, 1H, impurity), 1.92 (d, 1H, impurity)]. **^{13}C NMR** (75 MHz, D_2O) δ 150.6, 88.3, 82.0 (d, $J_{C,P}$ = 8.3 Hz), 71.5, 69.4, 64.5(d, $J_{C,P}$ = 5.2 Hz). **^{31}P NMR** (121 MHz, D_2O) δ 1.35. **HRMS** (ESI): m/z calculated for $C_8H_{12}N_3O_{10}P$ $[M-H]^-$: 340.0173, found: 340.0188.

b. Synthesis of Cytidine-5'-monophosphate (CMP) (adapted from [4])

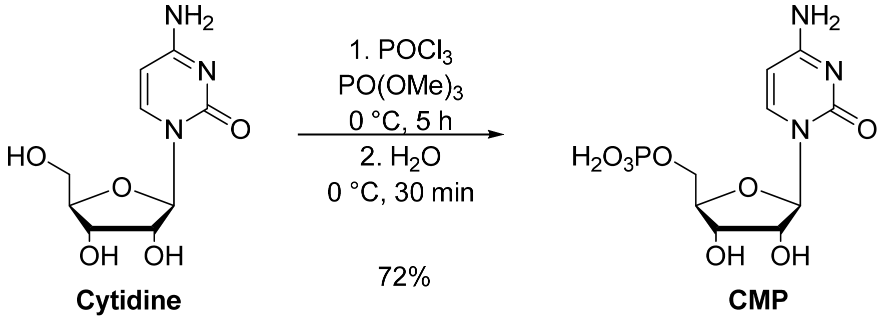

Figure S4. Synthesis of CMP.

Cytidine (1.0 g, 4.11 mmol, 1.0 eq.) was dissolved in $PO(OMe)_3$ (12 mL) under an argon atmosphere and cooled to 0 °C. Then, $POCl_3$ (0.77 mL, 8.22 mmol, 2.0 eq.) was added dropwise. The resulting solution was stirred for 5.5 h at 0 °C. Then, the mixture was poured into ice water (150 mL) and stirred for an additional 30 minutes. Aq. $NaHCO_3$ (25 mL) was added till a neutral pH was obtained. The solvent was removed under reduced pressure. The crude product was purified by automated flash chromatography (10% to 40% 2-propanol in NH_4OH-water (3:2)). **CMP** was obtained as a diammonium salt (1.06 g, 2.97 mmol, 72%). **^{1}H NMR** (400 MHz, D_2O) δ 8.00 (d, *J* = 7.6 Hz, 1H), 6.10 (d, *J* = 7.5 Hz, 1H), 6.01 – 5.94 (m, 1H), 4.34 – 4.29 (m, 2H), 4.24 (hept, *J* = 2.7 Hz, 1H), 4.13 (ddd, *J* = 11.8, 4.2, 2.5 Hz, 1H), 4.07 – 4.01 (m, 1H). **^{13}C NMR** (101 MHz, D_2O) δ 165.8, 157.3, 141.6, 96.4, 89.2, 83.0, 82.9, 74.2, 69.4, 63.5, 63.4. **^{31}P NMR** (162 MHz, D_2O) δ 1.42. **HRMS** (ESI): m/z calculated for $C_9H_{14}N_3O_8P_1$ $[M-H]^-$: 322.0446, found: 322.0449.

c. Synthesis of 5-methylorotidine-5'-monophosphate (5-Methyl OMP) (adapted from [1])

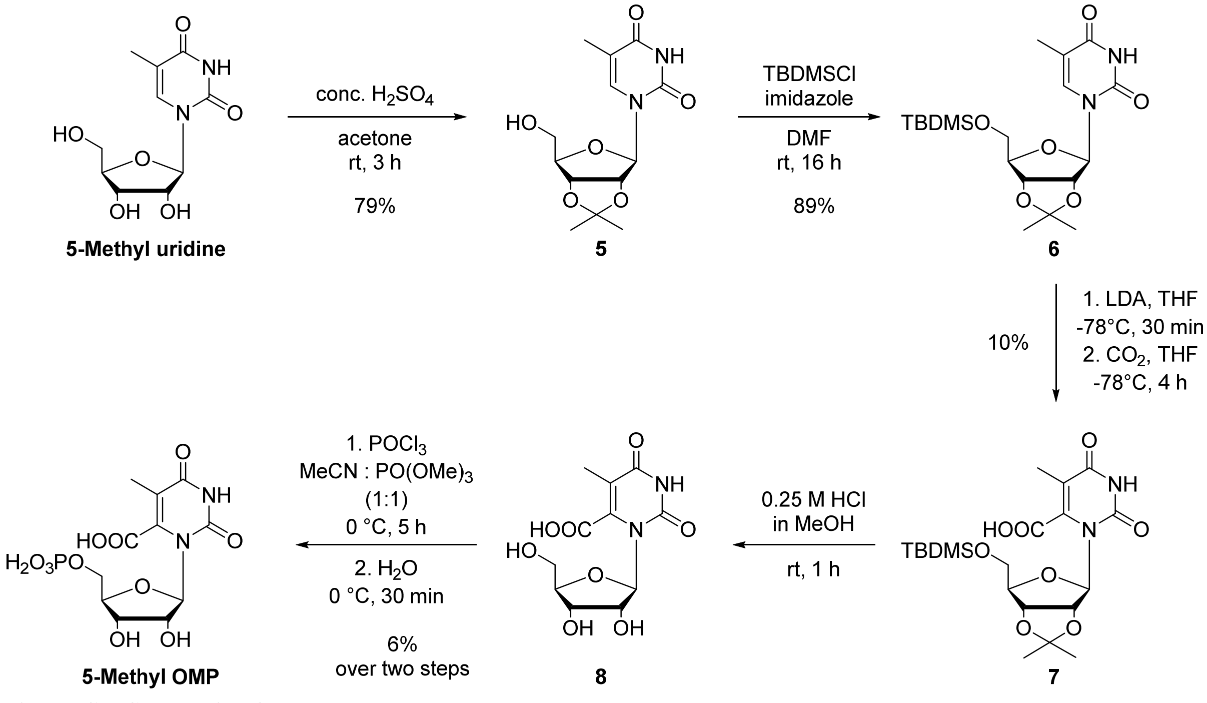

Figure S5. Synthesis of 5-Methyl OMP.

2',3'-*O*-Isopropylidene-5-methyluridine (5)

Concentrated H_2SO_4 (0.80 mL) was added to a solution of 5-methyluridine (3.00 g, 11.7 mmol, 1.0 eq.) in acetone (75 mL). The resulting solution was stirred for 4 h at room temperature. The reaction was quenched by using NEt_3 (3 mL), and the solvent was removed under reduced pressure. The crude product was purified by column chromatography (EtOAc/MeOH, 9:1). The title compound was obtained as a white solid (2.74 g, 9.20 mmol, 79%). $\mathbf{R_f}$ = 0.78 (EtOAc/MeOH, 9:1). **^{1}H NMR** (300 MHz, DMSO) δ 11.36 (s, 1H), 7.64 (q, J = 1.3 Hz, 1H), 5.83 (d, J = 2.8 Hz, 1H), 5.09 (t, J = 5.2 Hz, 1H), 4.88 (dd, J = 6.6, 2.8 Hz, 1H), 4.75 (dd, J = 6.5, 3.6 Hz, 1H), 4.06 – 3.98 (m, 1H), 3.58 (m, 2H), 1.76 (d, J = 1.2 Hz, 3H), 1.48 (s, 3H), 1.28 (s, 3H). **^{13}C NMR** (75 MHz, DMSO) δ 163.8, 150.4, 137.5, 113.1, 109.5, 90.4, 86.1, 83.4, 80.4, 61.2, 27.0, 25.2, 12.1. **IR** (ATR, cm^{-1}) ṽ 1682, 1475, 1455, 1374, 1266, 1213, 1157, 1068, 850, 781. **HRMS** (ESI): m/z calculated for $C_{13}H_{18}N_2O_6$ $[M+H]^+$: 299.1238, found: 299.1226; m/z calculated for $C_{13}H_{18}N_2O_6$ $[M+Na]^+$: 321.1057, found: 321.1049. **m.p.** = 120 – 122 °C

5'-*O*-*tert*-Butyldimethylsilyl-2',3'-*O*-isopropylidene-5-methyluridine (6)

2',3'-*O*-isopropylidene-5-methyluridine (2.20 g, 7.40 mmol, 1.0 eq., **5**), *tert*-butyldimethylsilyl chloride (TBDMSCl) (1.67 g, 11.1 mmol, 1.5 eq.), and imidazole (1.26 g, 18.5 mmol, 2.5 eq.) were dissolved in DMF (30 mL) under an argon atmosphere. The resulting solution was stirred at room temperature overnight. The solvent was removed *in vacuo*, and the crude product was purified by column chromatography (pentane/EtOAc, 1:1). The title product was obtained as a colourless oil (2.71 g, 6.58 mmol, 89%). $\mathbf{R_f}$ = 0.43 (pentane/EtOAc, 1:1). **^{1}H NMR** (300 MHz, DMSO) δ 11.39 (s, 1H), 7.48 (t, J = 1.4 Hz, 1H), 5.78 (d, J = 2.4 Hz, 1H), 4.91 (dd, J = 6.6, 2.5 Hz, 1H), 4.71 (dd, J = 6.5, 3.7 Hz, 1H), 4.10 – 4.03 (m, 1H), 3.77 (qd, J = 11.3, 4.7 Hz, 2H), 1.76 (d, J = 1.3 Hz, 3H), 1.48 (s, 3H), 1.29 (s, 3H), 0.86 (d, J = 1.1 Hz, 9H), 0.04 (s, 6H). **^{13}C NMR** (75 MHz, DMSO) δ 163.8, 150.3, 137.4, 113.1, 109.3, 91.2, 86.1, 83.6, 80.31, 63.1, 27.0, 25.8, 25.2, 18.0, 12.1, -5.4, -5.4. **IR** (ATR, cm^{-1}) ṽ 2927, 2854, 1686,

1460, 1372, 1253, 1211, 1076, 829, 774. **HRMS** (ESI): m/z calculated $C_{19}H_{32}N_2O_6Si$ $[M+H]^+$: 413.2102, found: 413.2089; m/z calculated for $C_{19}H_{32}N_2O_6Si$ $[M+Na]^+$: 435.1922, found: 435.1910.

5'-*O*-*tert*-butyldimethylsilyl-2',3'-*O*-isopropylidene-5-methylorotidine (7)

5'-*O*-*tert*-Butyldimethylsilyl-2',3'-*O*-isopropylidene-5-methyluridine (2.06 g, 4.99 mmol, 1.0 eq., **6**) was dissolved in THF (25 mL) under an argon atmosphere, and the solution was cooled to 78 °C. Then, lithium diisopropylamide (LDA) (2 mmol, 5.0 mL, 9.98 mmol, 2.0 equiv. was added slowly. The resulting solution was stirred for 30 min. Then, dry ice, as source of CO_2, was added every 10 min for 4 h at −78 °C. The reaction was allowed to warm up to room temperature and quenched by the addition of acetic acid (1 mL). The solvent was removed under vacuum. The crude product was purified by column chromatography (EtOAc/MeOH, 4:1), and the title compound was obtained as a yellowish solid (195 mg, 0.43 mmol, 10%). $\mathbf{R_f}$ = 0.27 (EtOAc/MeOH, 4:1). **^{1}H NMR** (300 MHz, DMSO) δ 11.08 (s, 1H), 5.64 (s, 1H), 5.07 (d, *J* = 6.3 Hz, 1H), 4.70 (dd, *J* = 6.3, 4.3 Hz, 1H), 3.89 (q, *J* = 6.2 Hz, 1H), 3.82 – 3.63 (m, 2H), 1.66 (s, 3H), 1.43 (s, 3H), 1.25 (s, 3H), 0.85 (s, 9H), 0.01 (d, *J* = 1.6 Hz, 6H). **^{13}C NMR** (75 MHz, DMSO) δ 164.7, 163.4, 153.6, 150.3, 112.4, 99.3, 93.5, 88.8, 84.6, 82.2, 63.9, 27.2, 25.8, 25.1, 18.0, 11.0, -5.2, -5.4. **IR** (ATR, cm^{-1}) ṽ 3385, 1718, 1679, 1635, 1585, 1395, 1382, 1211, 1065, 837. **HRMS** (ESI): m/z calculated for $C_{20}H_{32}N_2O_8Si$ $[M-H]^-$: 455.1855, found: 455.1860. **m.p.** = 174 – 176 °C.

5-Methylorotidine (8)

5'-*O*-*tert*-Butyldimethylsilyl-2',3'-*O*-isopropylidene-5-methylorotidine (195 mg, 0.43 mmol, 1.0 eq., **7**) was dissolved in methanolic HCl (0.25 M, 4 mL). The solution was stirred at room temperature for 1 h. The solvent was removed *in vacuo,* and the crude product (173 mg) was used in the next step without further purification. **^{1}H NMR** (300 MHz, DMSO) δ 11.59 (s, 1H), 5.17 (d, J = 4.1 Hz, 1H), 4.50 (dd, J = 6.1, 4.1 Hz, 1H), 4.00 (t, J = 6.1 Hz, 1H), 3.73 – 3.63 (m, 1H), 3.55 (m, 1H), 3.44 (dd, J = 11.7, 6.4 Hz, 1H), 1.75 (s, 3H). **HRMS** (ESI): m/z calculated for $C_{11}H_{14}N_2O_8$ $[M-H]^-$: 301.0677, found: 301.0684.

5-Methylorotidine-5'-monophosphate (5-Methyl OMP)

5-Methylorotidine (173 mg, 0.57 mmol, 1.0 eq.) was dissolved in MeCN/PO(OMe)$_3$ (1:1, 2 mL) under an argon atmosphere and cooled to 0 °C. Then, $POCl_3$ (0.10 mL, 1.13 mmol, 2.0 eq.) was added dropwise. The resulting solution was stirred at 0 °C for 5.5 h. Then, the mixture was poured into ice water (50 mL) and stirred for an additional 30 minutes. Aqueous $NaHCO_3$ (14 mL) was added until neutral pH was obtained. The solvent was removed under reduced pressure. The crude product was purified by automated flash chromatography (5% to 40% 2-propanol in NH_4OH-water (3:2)). **5-Methyl OMP** was obtained as diammonium salt (10.7 mg, 25.7 µmol, 6% over two steps). **^{1}H NMR** (300 MHz, D_2O) δ 5.37 (d, J = 3.0 Hz, 1H), 4.79 (1H, confirmed by HSQC), 4.44 (t, J = 6.7 Hz, 1H), 4.19 – 4.07 (m, 1H), 4.03 (m, 2H), 1.85 (s, 3H). [3.6 (d, 5.64 H, $(CH_3O)_3PO$))] **^{13}C NMR** (101 MHz, D_2O) δ 167.4, 166.8, 150.9, 150.7, 103.8, 95.7, 82.1, 82.1, 71.7, 69.2, 64.6, 52.4 $(CH_3O)_3PO$)), 10.3. **^{31}P NMR** (121 MHz, D_2O) δ 0.03. **HRMS** (ESI): m/z calculated for $C_{11}H_{15}N_2O_{11}P$ $[M-H]^-$: 381.0341, found: 381.0345.

d. Synthesis of 2'-Deoxy-oritidine-5'-monophosphate (Deoxy OMP) (adapted from [1])

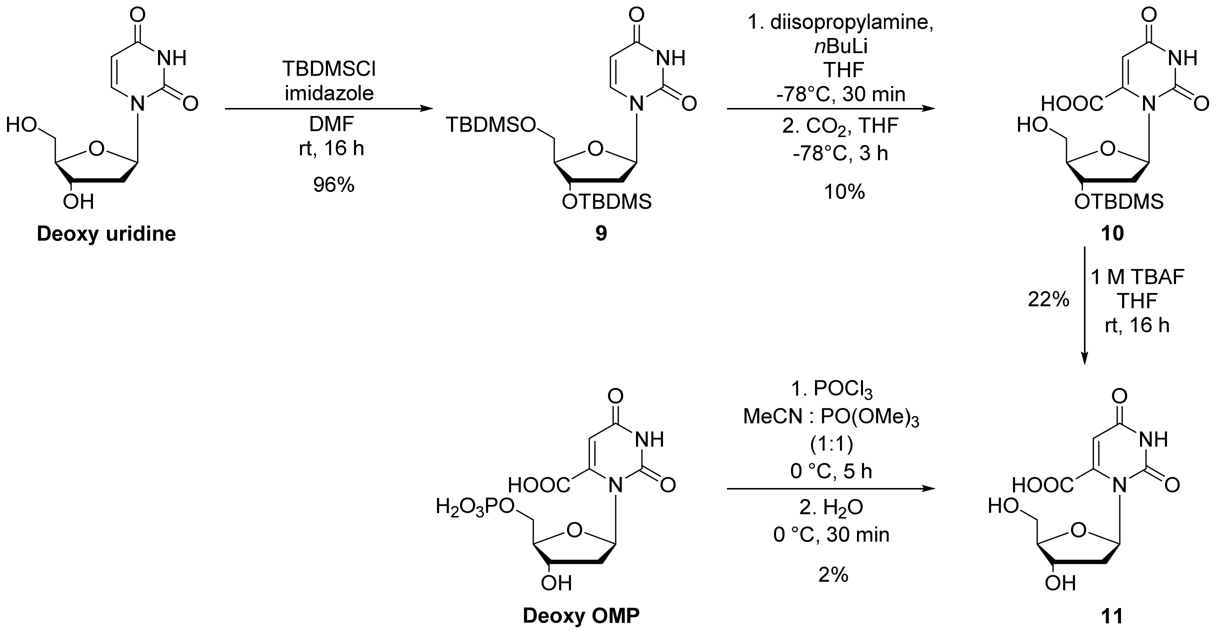

Figure S6. Synthesis of Deoxy OMP.

3',5'-Bi-*O*-di-tert-butyldimethylsilyl-2'-deoxy-uridine (9)

2'-Deoxy-uridine (2.00 g, 8.76 mmol, 1.0 eq.), imidazole (2.39 g, 35.0 mmol, 4.0 eq.), and TBDMSCl (4.13 g, 26.3 mmol, 3.0 eq.) were dissolved in DMF (25 mL) under an argon atmosphere. The solution was stirred at room temperature for 16 h. The solvent was removed *in vacuo,* and the crude product was purified by column chromatography using a pentane/EtOAc mixture (3:1). The desired compound was obtained as a white solid (3.82 g, 8.38 mmol, 96%). $\mathbf{R_f}$= 0.46 (pentane/EtOAc, 3:1). **^{1}H NMR** (300 MHz, DMSO) δ 11.32 (s, 1H), 7.70 (d, J = 8.1 Hz, 1H), 6.12 (t, J = 6.5 Hz, 1H), 5.58 (dd, J = 8.1, 2.0 Hz, 1H), 4.37 (d, J = 3.7 Hz, 1H), 3.80 – 3.62 (m, 3H), 2.28 – 2.07 (m, 2H), 0.87 (d, J = 3.4 Hz, 18H), 0.07 (d, J = 3.0 Hz, 12H). **^{13}C NMR** (101 MHz, DMSO) δ 163.0, 150.3, 140.2, 101.7, 86.7, 84.0, 71.4, 62.3, 40.0 (confirmed by HSQC), 25.7, 25.6, 18.0, 17.7, -4.8, -5.0, -5.5, -5.6. **HRMS** (ESI): m/z calculated for $C_{21}H_{40}N_2O_5Si_2$ $[M+H]^+$: 457.2549, found: 457.2537; m/z calculated for $C_{21}H_{40}N_2O_5Si_2$ $[M+Na]^+$: 479.2368, found: 479.2361. **IR** (ATR, cm^{-1}) ṽ 1693, 1459, 1248, 1119, 1083, 1060, 1028, 831, 806, 774. **m.p.** 117 – 119 °C.

3'-*O*-*tert*-Butyldimethylsilyl-6-carboxy-2'-deoxy-uridine (10)

3',5'-Bi-*O*-di-*tert*-butyldimethylsilyl-2'-deoxy-uridine (2.14 g, 4.68 mmol, 1.0 eq., **9**) was dissolved in THF (15 mL) under an argon atmosphere, and slowly added to LDA, freshly prepared from diisopropylamine (9 mL, 14.2 mmol, 3.03 eq.), *n*BuLi (2.5 M in hexane, 5.2 mL, 12.88 mmol, 2.75 eq.) in THF (10 mL) at −78°C. The resulting mixture was stirred for 30 min, and then CO_2 was bubbled through the solution at −78 °C for 3 h. Acetic acid (1.5 mL) was added, and the solvent was removed under reduced pressure. The crude product was purified by column chromatography (EtOAc/MeOH, 4:1). The final compound was obtained as a white solid with only one hydroxy group protected (177 mg, 0.46 mmol, 10%). $\mathbf{R_f}$= 0.19 (EtOAc/MeOH, 4:1). **^{1}H NMR** (400 MHz, DMSO) δ 5.98 (dd, *J* = 7.8, 5.9 Hz, 1H), 5.19 (d, *J* = 0.7 Hz, 1H), 4.76 (s, 1H), 4.44 (dt, *J* = 7.4, 4.5 Hz, 1H), 3.65 – 3.60 (m, 1H), 3.56 (dd, *J* = 11.7, 3.8 Hz, 1H), 3.45 (dd, *J* = 11.7, 5.8 Hz, 1H), 2.76 (dq, *J* = 11.5, 5.8 Hz, 1H), 1.94 (ddd, *J* = 12.5, 7.8, 4.4 Hz, 1H), 0.86 (d, *J* = 2.8 Hz, 9H), 0.12 – -0.02 (m, 6H). **^{13}C NMR** (101 MHz, DMSO) δ 164.2, 164.1, 158.4, 151.0, 96.7, 88.1, 87.9, 72.7, 62.1, 38.9, 26.2, 18.2, -4.3, -4.4. **HRMS** (ESI): m/z calculated for $C_{16}H_{25}N_2O_7Si$ $[M-H]^-$: 385.1437, found: 385.1434. **IR** (ATR, cm^{-1}) ṽ 2935, 2360, 1675, 1617, 1385, 1253, 1096, 1038, 837, 775. **m.p.** = 81 – 83 °C.

6-Carboxy-2'-deoxy-uridine (11)

3'-*O-tert*-Butyldimethylsilyl-6-carboxy-2'-deoxy-uridine (103 mg, 0.27 mmol, 1.0 eq., **10**) was dissolved in THF (1.35 mL), and tetra-*n*-butylammonium fluoride (TBAF) (1M in THF, 0.35 mmol, 347 µL, 1.3 eq.) was added to the solution. The reaction mixture was left to stir at room temperature for 16 h. The solvent was removed *in vacuo,* and the crude product was purified by column chromatography (DCM/MeOH, 4:1). The title product was obtained as a sticky oil (17 mg, 0.06 mmol, 22%), along with 67% of recovered starting material. $\mathbf{R_f}$ = 0.13 (DCM/MeOH, 7:3). **^{1}H NMR** (400 MHz, D_2O) δ 5.96 (dd, *J* = 8.0, 5.9 Hz, 1H), 5.73 (d, *J* = 0.7 Hz, 1H), 4.48 (dt, *J* = 8.0, 5.2 Hz, 1H), 3.97 – 3.89 (m, 1H), 3.88 – 3.72 (m, 2H), 2.88 (ddd, *J* = 14.0, 8.1, 5.9 Hz, 1H), 2.36 – 2.24 (m, 1H). **^{13}C NMR** (101 MHz, D_2O) δ 167.5, 166.2, 154.9, 151.0, 98.3, 88.7, 86.6, 70.7, 61.7, 37.6. **HRMS** (ESI): m/z calculated for $C_{10}H_{12}N_2O_7$ $[M-H]^-$: 271.0572, found: 271.0575.

2'-Deoxy-orotidine-5'-monophosphate (Deoxy OMP)

6-Carboxy-2'-deoxy-uridine (66 mg, 0.24 mmol, 1.0 eq., **11**) was dissolved in MeCN/PO(OMe)$_3$ (1:1, 2 mL) under an argon atmosphere and cooled to 0 °C. Then, $POCl_3$ (0.10 mL, 0.83 mmol, 3.5 eq.) was added dropwise. The resulting solution was stirred at 0 °C for 5 h. Then, the mixture was poured into ice water (50 mL) and stirred for an additional 30 minutes. Aqueous $NaHCO_3$ (approx. 19 mL) was added until neutral pH was obtained. The solvent was removed under reduced pressure. Anion exchange HPLC purified the crude product. **Deoxy OMP** was obtained as di-triethylammonium salt (2.34 mg, 4 µmol, 2%). $\mathbf{R_t}$ = 14 min. **^{1}H NMR** (300 MHz, D_2O) δ 5.93 (dd, *J* = 8.3, 5.2 Hz, 1H), 5.73 (s, 1H), 4.11 (d, *J* = 6.0 Hz, 1H), 3.97 (t, *J* = 7.2 Hz, 2H), 2.97 (dd, *J* = 14.6, 7.3 Hz, 1H), 2.47 (d, *J* = 5.7 Hz, 1H). The ^{1}H signals at 5.75 (s, 5-H) and 5.95 (t, 1′-H) were previously reported to identify significant characteristics of the same molecule.[5] We completed the analysis; however, the signal of functional group 3'-H could not be resolved. **^{31}P NMR** (121 MHz, D_2O) δ 1.18. **HRMS** (ESI): m/z calculated for $C_{10}H_{13}N_2O_{10}P$ $[M-H]^-$: 351.0235, found: 351.0230.

e. Deoxyuridine-5'-monophosphate (Deoxy UMP) (adapted from [4])

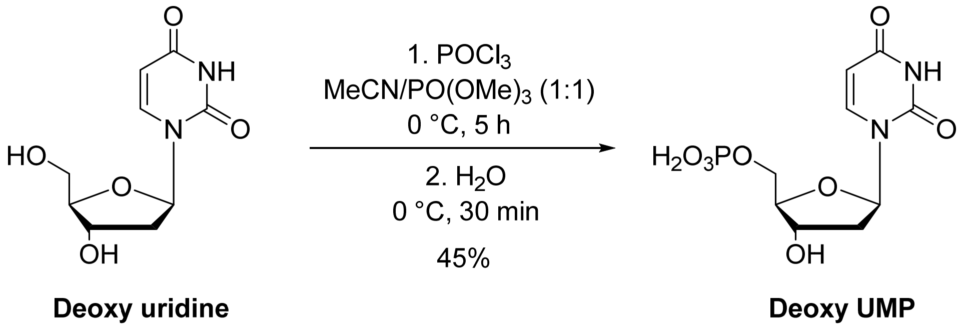

Figure S7. Synthesis of deoxy UMP.

Deoxy uridine (100 mg, 0.44 mmol, 1.0 eq.) was dissolved in MeCN/PO(OMe)$_3$ (1:1, 2 mL) under an argon atmosphere and cooled to 0 °C. Then, $POCl_3$ (72 µL, 0.88 mmol, 2.0 eq.) was added dropwise. The resulting solution was stirred at 0 °C for 5.5 h. Then, the mixture was poured into ice water (50 mL) and stirred for an additional 30 minutes. Aqueous $NaHCO_3$ (15 mL) was added until a neutral pH was obtained. The solvent was removed under reduced pressure. The crude product was purified by automated flash chromatography (20% to 60% 2-propanol in NH_4OH-water (3:2)). **Deoxy UMP** was obtained as a diammonium salt (70.0 mg, 0.20 mmol, 45%). **^{1}H NMR** (300 MHz, D_2O) δ 8.02 (d, *J* = 8.1 Hz, 1H), 6.31 (t, *J* = 6.8 Hz, 1H), 5.93 (d, *J* = 8.1 Hz, 1H), 4.56 (td, *J* = 4.8, 3.1 Hz, 1H), 4.15 (qd, *J* = 3.5, 1.4 Hz, 1H), 3.96 (ddd, *J* = 5.4, 3.6, 1.7 Hz, 2H), 2.37 (dd, *J* = 6.9, 4.9 Hz, 2H). **^{13}C NMR** (101 MHz, D_2O) δ 166.3, 151.7, 142.2, 102.4, 86.2, 86.1, 85.3, 71.1, 63.8, 38.8. **^{31}P NMR** (162 MHz, D_2O) δ 2.83. **HRMS** (ESI): m/z calculated for $C_9H_{13}N_2O_8P_1$ $[M-H]^-$: 307.0337, found: 307.0333.

f. Synthesis of 2'-SH UMP

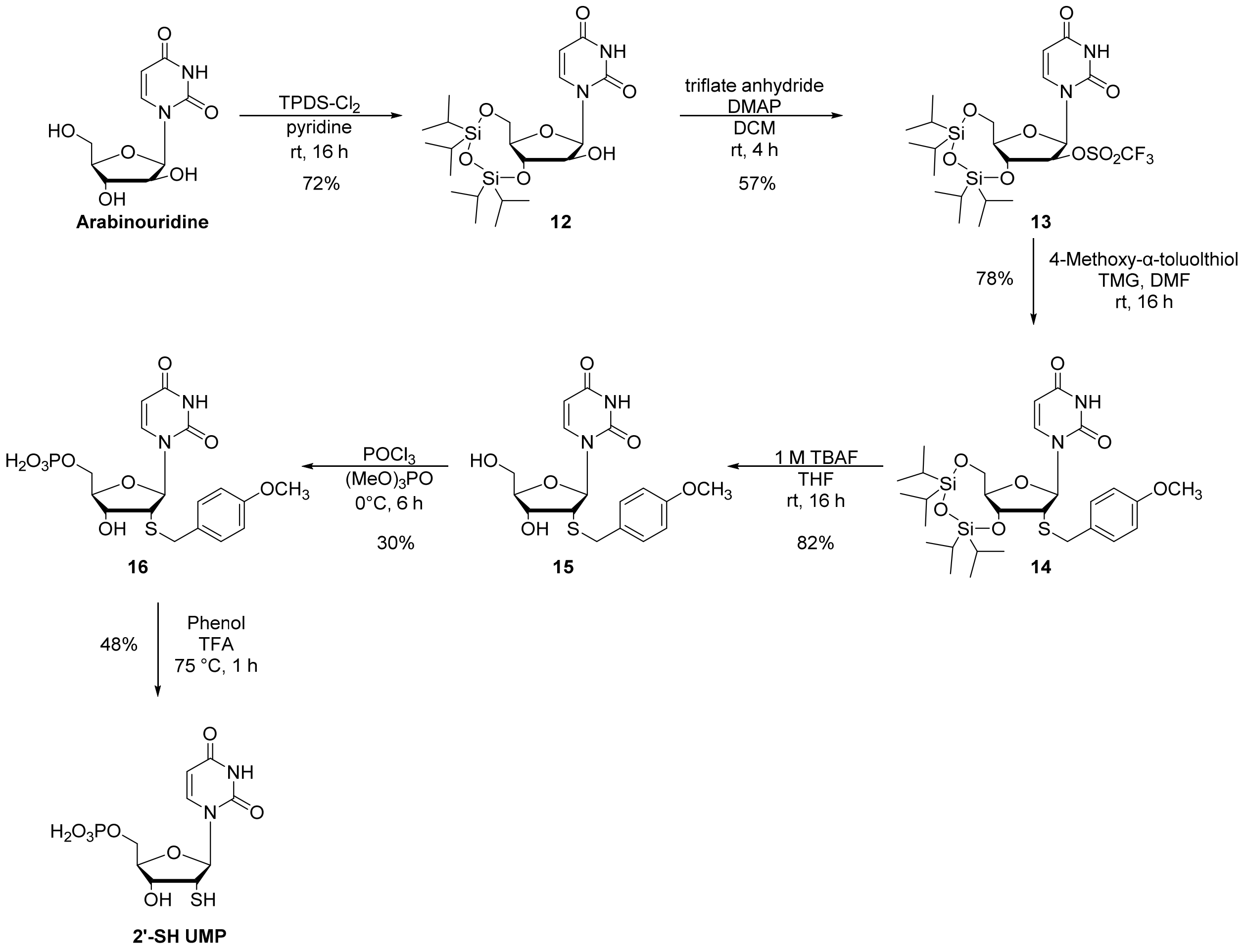

Figure S8. Synthesis of 2'-SH UMP.

3′,5′-*O*-(Tetraisopropyldisiloxane-1,3-diyl)-1-β-D-arabino-furanosyluracil (12)[6]

1-β-D-Arabinofuranosyluracil (2.00 g, 8.10 mmol, 1.0 eq.) was dissolved in dry pyridine (40 mL) under an argon atmosphere. 1,3-Dichloro-1,1,3,3-tetraisopropyldisiloxane ($TIPDSiCl_2$) (3.5 mL, 11.0 mmol, 1.3 eq.) was added slowly at 0 °C over 5 min. The reaction mixture was stirred at room temperature for 19 h. The reaction was cooled to 0°C, and water (5 mL) was added. Then, the solvent was removed under reduced pressure. The crude compound was dissolved in EtOAc (100 mL) and washed with water (80 mL), sat. $NaHCO_3$ (80 mL), brine (80 mL), and dried over Na_2SO_4. The solvent was removed *in vacuo,* and the collected solid was dissolved in MeCN (140 mL). The suspension was filtered off, further washed with MeCN (20 mL), and the solid was collected. The compound was purified by flash chromatography (DCM/MeOH, 95:5), and the title compound was obtained as a white solid (2.88 g, 5.91 mmol, 72%). $\mathbf{R_f}$= 0.44 (DCM/MeOH, 95:5). **^{1}H NMR** (400 MHz, $CDCl_3$) δ 9.95 (s, 1H), 7.83 (d, J = 8.1 Hz, 1H), 6.08 (d, J = 6.1 Hz, 1H), 5.70 (dd, J = 8.2, 1.3 Hz, 1H), 4.56 (ddd, J = 8.5, 6.1, 4.8 Hz, 1H), 4.28 (d, J = 4.9 Hz, 1H), 4.18 – 4.07 (m, 2H), 4.00 (dd, J = 13.4, 2.8 Hz, 1H), 3.76 (ddd, J = 9.0, 2.7, 1.5 Hz, 1H), 1.15 – 0.86 (m, 28H). **^{13}C NMR** (101 MHz, $CDCl_3$) δ 164.5, 151.7, 140.9, 101.9, 84.6, 81.0, 75.6, 71.9, 60.3, 17.7, 17.6, 17.6, 17.5, 17.3, 17.2, 17.1, 17.0, 17.0, 13.7, 13.3, 13.2, 13.1, 12.5. **HRMS** (ESI): m/z calculated for $C_{21}H_{38}N_2O_7Si_2$ $[M+H]^+$ 487.2290, found: 487.2282; m/z calculated for $C_{21}H_{38}N_2O_7Si_2$ $[M+Na]^+$ 509.2110, found: 509.2110.

3′,5′-*O*-(Tetraisopropyldisiloxane-1,3-diyl)-2’-triflyl-1-β-D-arabino-furanosyluracil (13) [6]

3′,5′-*O*-(Tetraisopropyldisiloxane-1,3-diyl)-1-β-D-arabino-furanosyluracil (3.22 g, 6.61 mmol, 1.0 eq., **12**) was dissolved in dry CH_2Cl_2 (33 mL) under an argon atmosphere. The solution was cooled to 0°C and 4-dimethylaminopyridine (DMAP) (2.42 g, 19.8 mmol, 3.0 eq.) was added. Triflate anhydride (1.67 mL, 9.92 mmol, 1.5 eq.) was added slowly over 5 min. The reaction mixture was stirred for 4 h at room temperature. Water (5 mL) was added, and the solvent was removed under reduced pressure. The mixture was dissolved in EtOAc (150 mL), and then washed with saturated NH_4Cl (55 mL), brine (55 mL), and dried over Na_2SO_4. The organic solvent was removed under reduced pressure. The crude product was purified by flash chromatography using EtOAc (100%). The title compound was collected as a white solid (2.31 g, 3.74 mmol, 57%). **R_f**= 0.63 (EtOAc/Pentane, 1:1) **^{1}H NMR** (400 MHz, $CDCl_3$) δ 9.11 (s, 1H), 7.48 (d, *J* = 8.2 Hz, 1H), 6.28 (d, *J* = 5.3 Hz, 1H), 5.77 (dd, *J* = 8.2, 2.1 Hz, 1H), 5.38 (t, *J* = 5.3 Hz, 1H), 4.67 – 4.58 (m, 1H), 4.11 (dd, *J* = 12.7, 3.5 Hz, 1H), 4.03 (dd, *J* = 12.7, 4.9 Hz, 1H), 3.87 (ddd, *J* = 7.0, 4.9, 3.4 Hz, 1H), 1.16 – 0.94 (m, 28H). **^{13}C NMR** (101 MHz, $CDCl_3$) δ 162.7, 150.0, 139.7, 123.2, 120.0, 116.8, 103.1, 88.1, 81.8, 81.5, 74.2, 61.3, 17.5, 17.4, 17.4, 17.3, 17.0, 16.9, 16.8, 16.7, 13.5, 13.2, 13.1, 12.6. **^{19}F NMR** (377 MHz, $CDCl_3$) δ -74.36. **HRMS** (ESI): m/z calculated for $C_{22}H_{37}F_3N_2O_9S_1Si_2$ $[M+H]^+$ 619.1783, found: 619.1785; m/z calculated for $C_{22}H_{37}F_3N_2O_9S_1Si_2$ $[M+Na]^+$ 641.1603, found: 641.1604.

3′,5′-*O*-(tetraisopropyldisiloxane-1,3-diyl)-2’-(4-methoxy)toluolthiol-1-β-D-arabino-furanosyluracil (14) (adapted from [7])

3′,5′-*O*-(Tetraisopropyldisiloxane-1,3-diyl)-2’-triflyl-1-β-D-arabino-furanosyluracil (2.28 g, 3.68 mmol, 1.0 eq., **13**) was dissolved in dry DMF (16 mL) under an argon atmosphere. The solution was cooled to 0°C, and tetramethylguanidine (600 µL, 4.78 mmol, 1.3 equiv.) and 4-methoxy-alpha-toluolthiol (1.41 g, 9.16 mmol, 2.5 equiv.) were added. The reaction mixture was stirred at room temperature for 24 h. The solvent was removed under reduced pressure, and the crude compound was purified by flash column chromatography (20% to 40% EtOAc in pentane). The title compound was obtained as a colourless oil (1.79 g, 2.87 mmol, 78%). **R_f**= 0.97 (EtOAc/MeOH, 4:1) **^{1}H NMR** (400 MHz, CDCl3) δ 9.05 (d, J = 2.2 Hz, 1H), 7.80 (d, J = 8.1 Hz, 1H), 7.33 – 7.23 (m, 2H), 6.85 – 6.76 (m, 2H), 6.04 (d, J = 1.3 Hz, 1H), 5.67 (dd, J = 8.1, 2.2 Hz, 1H), 4.47 (dd, J = 8.8, 6.7 Hz, 1H), 4.17 (dd, J = 13.3, 1.8 Hz, 1H), 4.08 (ddd, J = 8.9, 2.6, 1.6 Hz, 1H), 4.03 (d, J = 13.4 Hz, 1H), 3.99 – 3.90 (m, 2H), 3.77 (s, 3H), 3.31 (dd, J = 6.6, 1.3 Hz, 1H), 1.13 – 0.97 (m, 28H). **^{13}C NMR** (101 MHz, $CDCl_3$) δ 163.5, 158.8, 150.0, 139.7, 130.3, 129.7, 114.0, 101.9, 90.7, 83.6, 68.1, 60.0, 55.4, 53.2, 34.5, 17.6, 17.5, 17.4, 17.4, 17.1, 17.1, 17.0, 16.9, 13.5, 13.2, 13.0, 12.6. **HRMS** (ESI): m/z calculated for $C_{29}H_{46}N_2O_7S_1Si_2$ $[M+Na]^+$ 645.2456, found: 645.2451. **IR** (ATR, cm^{-1}) ṽ 2942, 2929, 2868, 2009, 1685, 1507, 1461, 1247, 1115, 1033.

1-((2R,4R,5R)-4-hydroxy-5-(hydroxymethyl)-3-((4-methoxybenzyl)thio)tetrahydrofuran-2-yl)pyrimidine-2,4(1H,3H)-dione (15)

3′,5′-*O*-(Tetraisopropyldisiloxane-1,3-diyl)-2’-(4-methoxy)toluolthiol-1-β-D-arabino-furano-syluracil (311 mg, 0.50 mmol, 1.0 eq., **14**) was dissolved in THF (2.5 mL), and TBAF (1 M in THF, 1.0 mmol, 1.00 mL, 2.0 eq.) was added to the solution. The reaction mixture was left to stir at room temperature for 16 h. The solvent was removed *in vacuo,* and the crude product was purified by column chromatography (EtOAc/MeOH, 95:5). The title product was obtained as a white solid (156 mg, 0.41 mmol, 82%). **R_f**= 0.4 (EtOAC, 100%) **^{1}H NMR** (400 MHz, DMSO) δ 11.33 (d, *J* = 2.3 Hz, 1H), 7.69 (d, *J* = 8.1 Hz, 1H), 7.22 – 7.09 (m, 2H), 6.84 – 6.75 (m, 2H), 6.03 (d, *J* = 8.6 Hz, 1H), 5.64 (d, *J* = 5.3 Hz, 1H), 5.56 (dd, *J* = 8.1, 2.2 Hz, 1H), 5.10 (t, *J* = 5.1 Hz, 1H), 4.16 (td, *J* = 5.1, 2.0 Hz, 1H), 3.96 – 3.83 (m, 1H), 3.72 (s, 3H), 3.64 (d, *J* = 3.7 Hz, 2H), 3.54 (t, *J* = 3.9 Hz, 2H), 3.36 – 3.28 (m, 1H). **^{13}C NMR** (101 MHz, DMSO) δ 162.9, 158.3, 150.7, 140.1, 129.9, 129.8, 113.8, 102.3, 87.5, 86.7, 71.9, 61.4, 55.0, 51.7, 34.0. **HRMS** (ESI): m/z calculated for $C_{17}H_{20}N_2O_6S_1$ $[M-H]^-$ 379.0969, found: 379.0971; m/z calculated for $C_{17}H_{20}N_2O_6S_1$ $[M+Na]^+$ 403.0934, found: 403.0946.

((2*R*,3*R*,5*R*)-5-(2,4-Dioxo-3,4-dihydropyrimidin-1(2H)-yl)-3-hydroxy-4-((4-methoxybenzyl)thio)tetrahydrofuran-2-yl)methyl dihydrogen phosphate (16)

1-((2*R*,4*R*,5*R*)-4-hydroxy-5-(hydroxymethyl)-3-((4-methoxybenzyl)thio)tetrahydrofuran-2-yl)pyrimidine-2,4(1H,3H)-dione (147 mg, 0.39 mmol, 1.0 eq., **15**) was dissolved in dry $PO(OMe)_3$ (1 mL) under argon atmosphere and cooled to 0 °C. Then, $POCl_3$ (72 µL, 0.77 mmol, 2.0 eq.) was added dropwise. The resulting solution was stirred at 0 °C for 6 h. Then, the mixture was poured into ice water (50 mL) and stirred for an additional 30 minutes. Aqueous $NaHCO_3$ (8.5 mL) was added until a neutral pH was obtained. The solvent was removed under reduced pressure. The crude product was purified by automated flash chromatography (10 % to 40 % 2-propanol in NH_4OH-water (3:2)). The title compound was obtained as a white solid (58.3 mg, 11.9 µmol, 30%). **^{1}H NMR** (400 MHz, D_2O) δ 7.53 (dd, *J* = 8.2, 0.9 Hz, 1H), 7.25 – 7.17 (m, 2H), 6.87 – 6.79 (m, 2H), 5.98 (dd, *J* = 8.8, 4.0 Hz, 1H), 5.60 (d, *J* = 8.0 Hz, 1H), 4.52 (d, *J* = 5.3 Hz, 1H), 4.26 (tt, *J* = 3.1, 1.9 Hz, 1H), 4.07 – 3.98 (m, 2H), 3.79 (d, *J* = 0.9 Hz, 3H), 3.70 (d, *J* = 3.7 Hz, 2H), 3.41 – 3.33 (m, 1H). **^{13}C NMR** (101 MHz, DMSO) δ 165.5, 157.9, 151.5, 140.7, 131.3, 129.9, 114.1, 103.1, 87.9, 85.6, 85.5, 72.8, 64.8, 64.8, 55.1, 52.4, 35.1. **^{31}P NMR** (162 MHz, D_2O) δ 1.16. **HRMS** (ESI): m/z calculated for $C_{17}H_{21}N_2O_9P_1S_1$ $[M\text{-}H]^-$ 459.0633, found: 459.0641.

2'-Thiouridine-5'-monophosphate (**2'-SH UMP**) (adapted from [7])

To a solution of ((2*R*,3*R*,5*R*)-5-(2,4-dioxo-3,4-dihydropyrimidin-1(2H)-yl)-3-hydroxy-4-((4-methoxybenzyl)-thio)tetrahydrofuran-2-yl)methyl dihydrogen phosphate (17.1 mg, 34.6 µmol, 1.0 eq.) in TFA (180 µL), phenol (6.0 mg, 60.0 µmol, 1.6 eq.) was added. The reaction mixture was stirred at 75 °C for 1 h. The solvent was removed *in vacuo,* and the residue was co-evaporated with MeCN (three times, 5 mL each). The crude product was purified by automated flash chromatography (20 % to 50 % 2-propanol in NH_4OH-water (3:2)). The title compound was obtained as a diammonium salt (6.17 mg, 16.5 µmol, 48%). **^{1}H NMR** (400 MHz, D_2O) δ 7.97 (dd, *J* = 8.2, 1.6 Hz, 1H), 6.34 (dd, *J* = 8.8, 1.6 Hz, 1H), 6.01 (dd, *J* = 8.3, 1.6 Hz, 1H), 4.54 (dd, *J* = 5.6, 1.8 Hz, 1H), 4.27 (q, *J* = 2.2 Hz, 1H), 4.06 – 4.00 (m, 2H), 3.82 – 3.77 (m, 1H). **^{13}C NMR** (101 MHz, D_2O) δ 165.9, 151.9, 141.6, 103.4, 88.2, 85.9, 72.39, 64.4, 56.0. **^{31}P NMR** (162 MHz, D_2O) δ 1.35. **HRMS** (ESI): m/z calculated for $C_9H_{13}N_2O_8P_1S_1$ $[M\text{-}H]^-$ 339.0057, found: 339.0061.

4. Isothermal Calorimetry

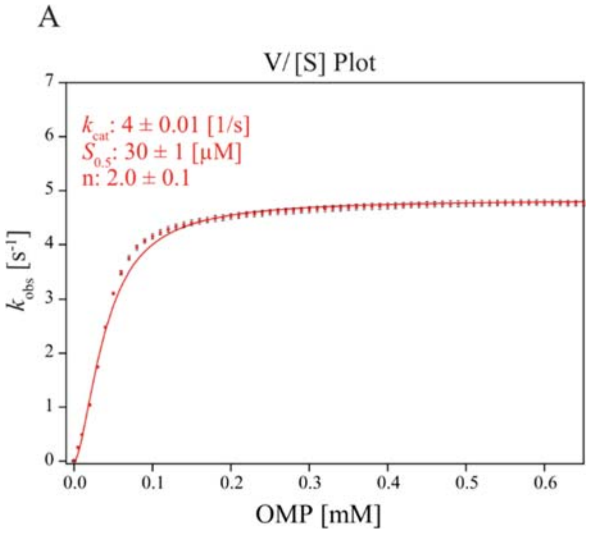

Figure S9. Human OMPDC kinetics for conversion of the natural substrate OMP. The v_[S] plot and the fit according to the Hill equation are shown.

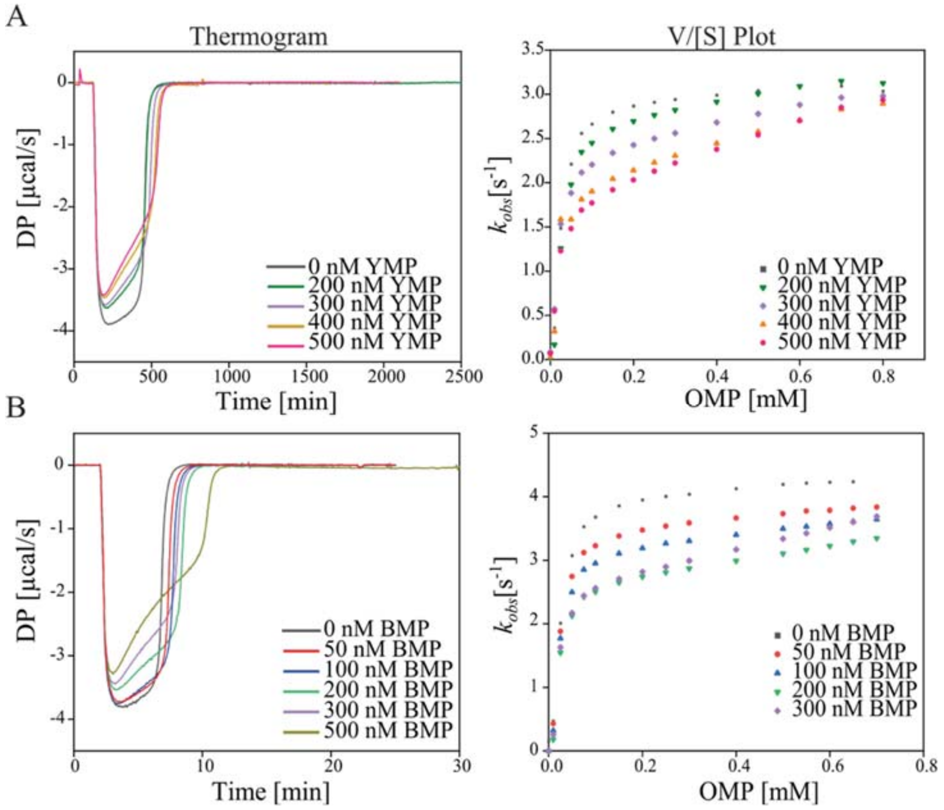

Figure S10. Turnover of substrate OMP in the presence of inhibitors YMP (A) and BMP (B), showing both the corresponding thermograms and the calculated v_S plots. The marked cooperativity of OMPDCase towards the substrate precluded a reliable determination of the inhibitory constant K_i of both inhibitors under turnover conditions.

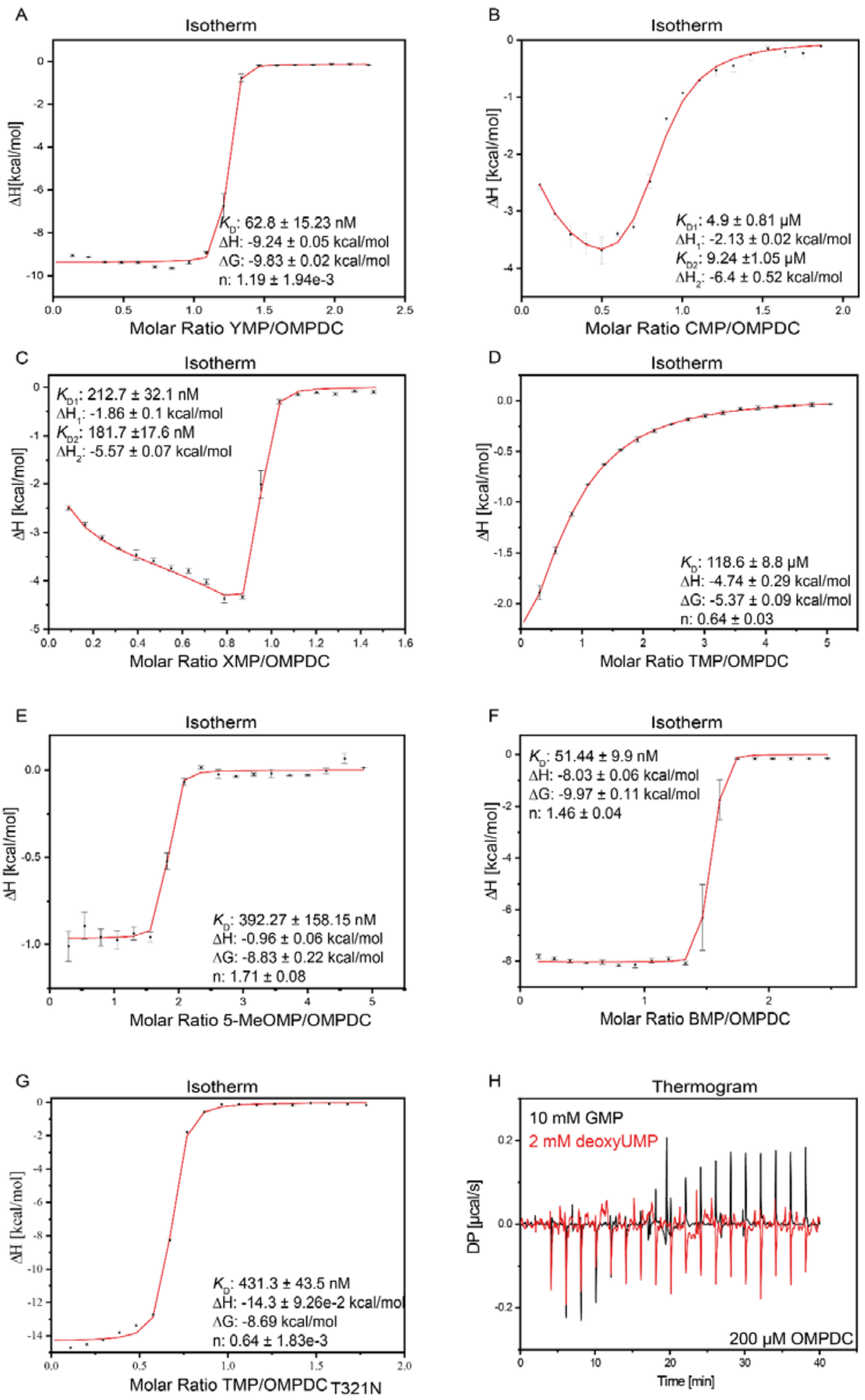

Figure S11. Binding isotherms of human OMPDC towards YMP (A), CMP (B), XMP (C), dTMP (D), 5-Methyl OMP (E), and BMP (F). The binding affinity for dTMP was also assessed for OMPDC variant T321N (G). No binding could be detected for GMP and deoxy UMP towards OMPDC wild-type (H).

Table S1. A concentration error of 5 % or 10 % was added to the ligands and/or protein concentration to probe the influence on the thermodynamic values obtained by ITC. Measurements were conducted at 25°C.

**Ligand**	**Ligand [mM]**	**OMPDC [µM]**	***K*** K_D **[M]**	**ΔH [kcal/mol]**	**ΔG [kcal/mol]**	**-TΔS [kcal/mol]**	**n**
BMP	2.28 mM	192 uM	3.36E-08 2.25E-9	-8.74 ± 0.04	-10.2 ± 0.06	-1.46 ± 0.07	1.33 ± 0.001
YMP	2.03 mM	187 uM	5.72E-08 ± 1.02E-8	-10.17 ± 0.06	-9.89 ± 0.02	0.26 ± 0.02	1.08 ± 0.002
**- 5 % error on ligand**							
BMP	2.17 mM	192 uM	3.16E-08 ± 2.26E-9	-9.18 ± 0.04	-10.26 ± 0.03	-1.06 ± 0.07	1.27 ± 0.04
YMP	1.93 mM	187 uM	5.44E-08 ± 1.79E-9	-10.67 ± 0.03	-9.92 ± 0.02	0.76 ± 0.03	1.03 ± 0.01
**+ 5 % error on ligand**							
BMP	2.39 mM	192 uM	3.54E-08 ± 2.3E-9	-8.34 ± 0.03	-10.17 ± 0.03	-1.84 ± 0.07	1.40 ± 0.04
**YMP**	2.13 mM	187 uM	6.03E-08 ± 1.98E-9	-9.67 ± 0.04	-9.86 ± 0.02	-0.19 ± 0.02	1.13 ± 0.01
**- 5 % error on protein**							
BMP	2.28 mM	182 uM	3.38E-08 ± 2.21E-9	-8.74 ± 0.03	-10.2 ± 0.06	-1.46 ± 0.07	1.41 ± 0.04
YMP	2.03 mM	178 uM	5.72E-08 ± 1.89E-9	-10.17 ± 0.03	-9.89 ± 0.02	0.26 ± 0.02	1.13 ± 0.01
**+ 5 % error on protein**							
BMP	2.28 mM	202 uM	3.36E-08 ± 2.26E-9	-8.74 ± 0.03	-10.2 ± 0.06	-1.46 ± 0.07	1.27 ± 0.03
YMP	2.03 mM	196 uM	5.72E-08 ± 1.89E-9	-10.17 ± 0.03	-9.89 ± 0.02	0.26 ± 0.02	1.03 ± 0.01
**+ 5 % error on protein and - 5 % error ligand**							
BMP	2.17 mM	202 uM	3.18E-08 ± 2.19E-9	-9.18 ± 0.04	-10.27 ± 0.03	-1.05 ± 0.07	1.20 ± 0.03
YMP	1.93 mM	196 uM	5.44E-08 ± 1.79E-9	-10.67 ± 0.03	-9.92 ± 0.02	0.76 ± 0.025	0.98 ± 0.008
**- 10 % error on ligand**							
BMP	2.05 mM	192 uM	3.02E-08 ± 2.04E-9	-9.72 ± 0.04	-10.27 ± 0.03	-0.55 ± 0.07	1.2 ± 0.03
YMP	1.83 mM	187 uM	5.16E-08 ± 1.72E-9	-11.27 ± 0.03	-9.95 ± 0.02	1.31 ± 0.03	0.97 ± 0.008

5. NMR Spectra

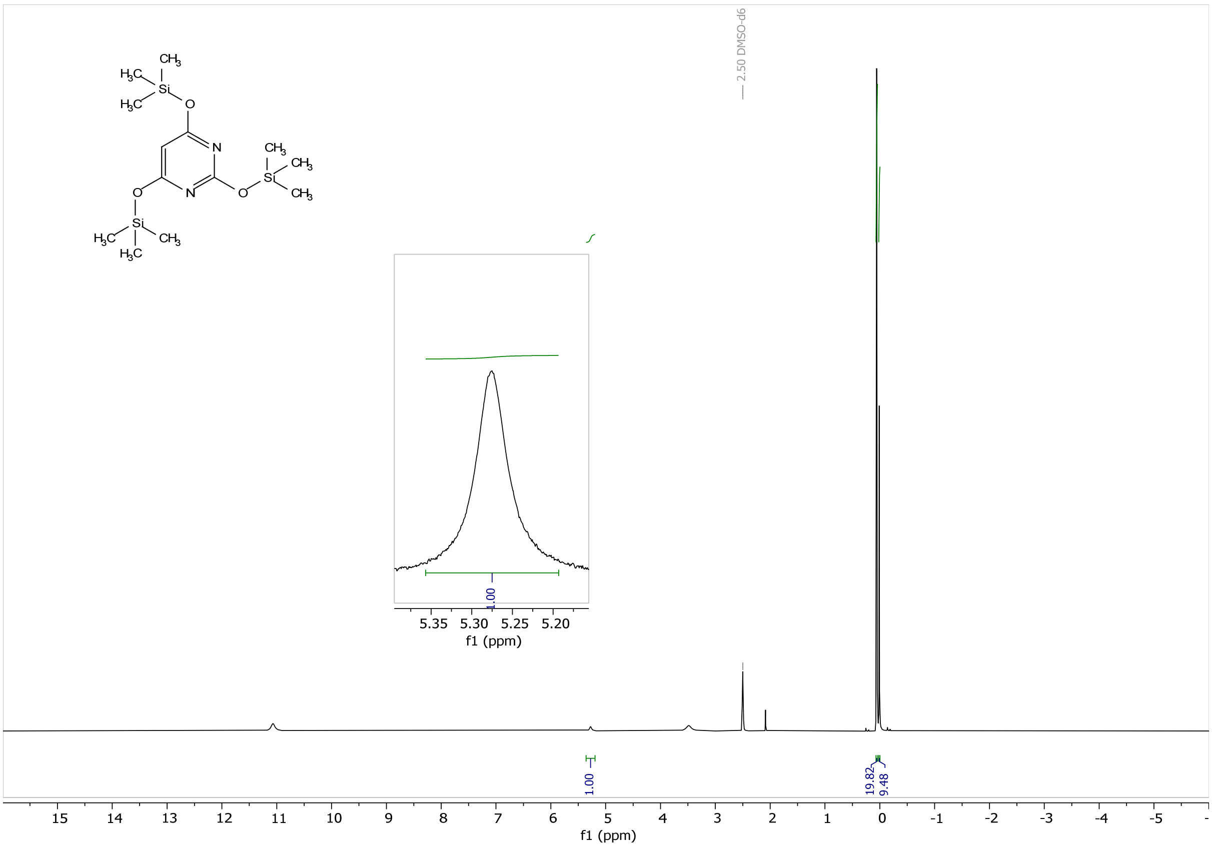

Figure S12. ^{1}H NMR of 2,4,6-Tris(trimethylsilyloxy)pyrimidine (1a) in DMSO-*d*$_6$. Barbituric acid signals at 3.48 and 11.07 ppm.

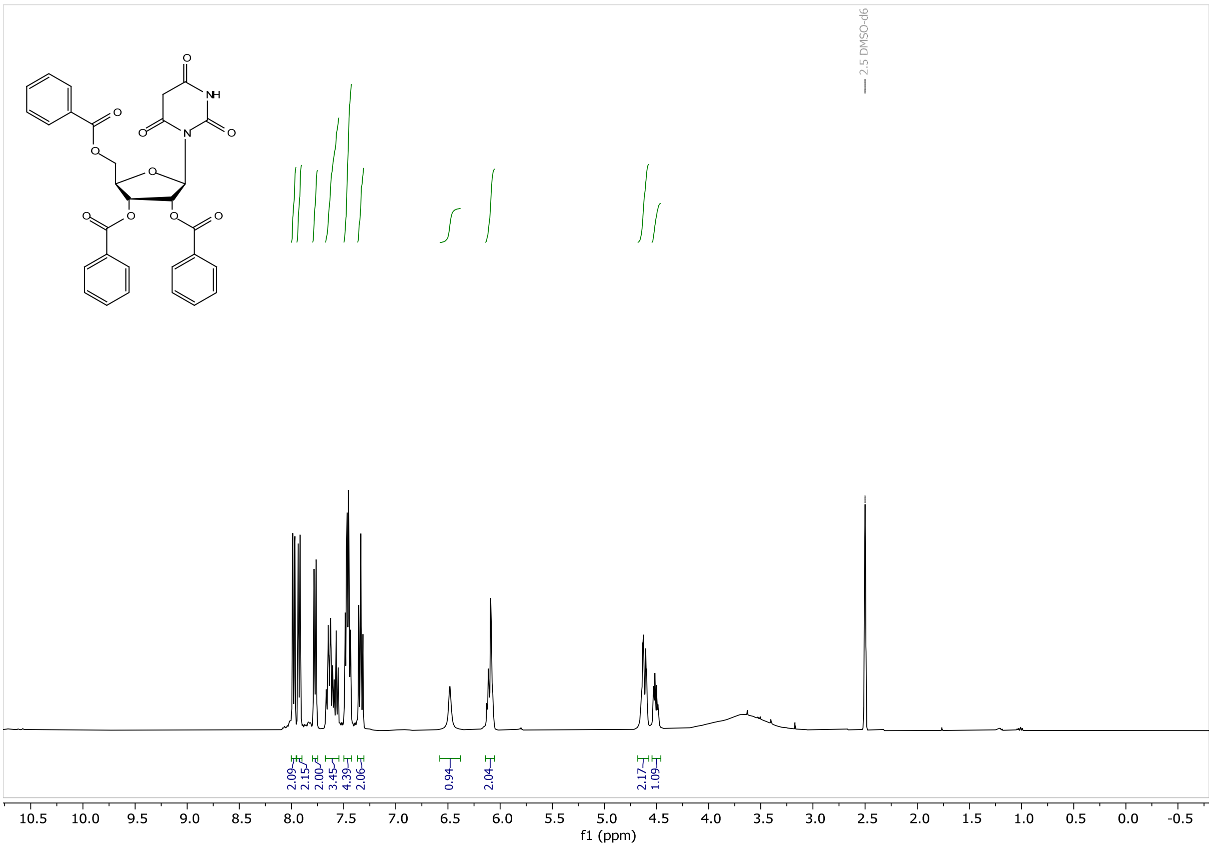

Figure S13. **^{1}H NMR of 2',3',5'-Tri-O-benzoyl-6-hydroxyuridine (3a) in DMSO-*d*[6].**

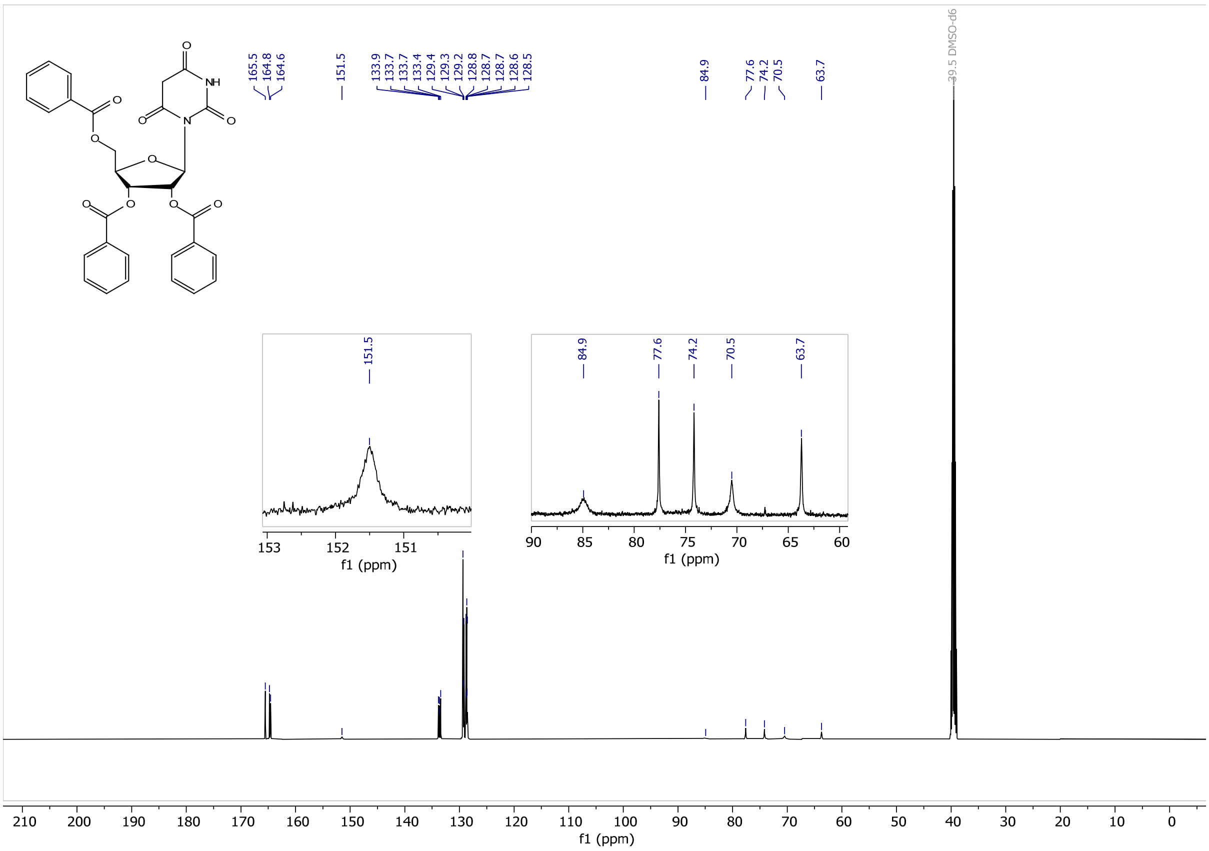

Figure S14. **^{13}C NMR of 2',3',5'-Tri-O-benzoyl-6-hydroxyuridine (3a) in DMSO-*d*[6].**

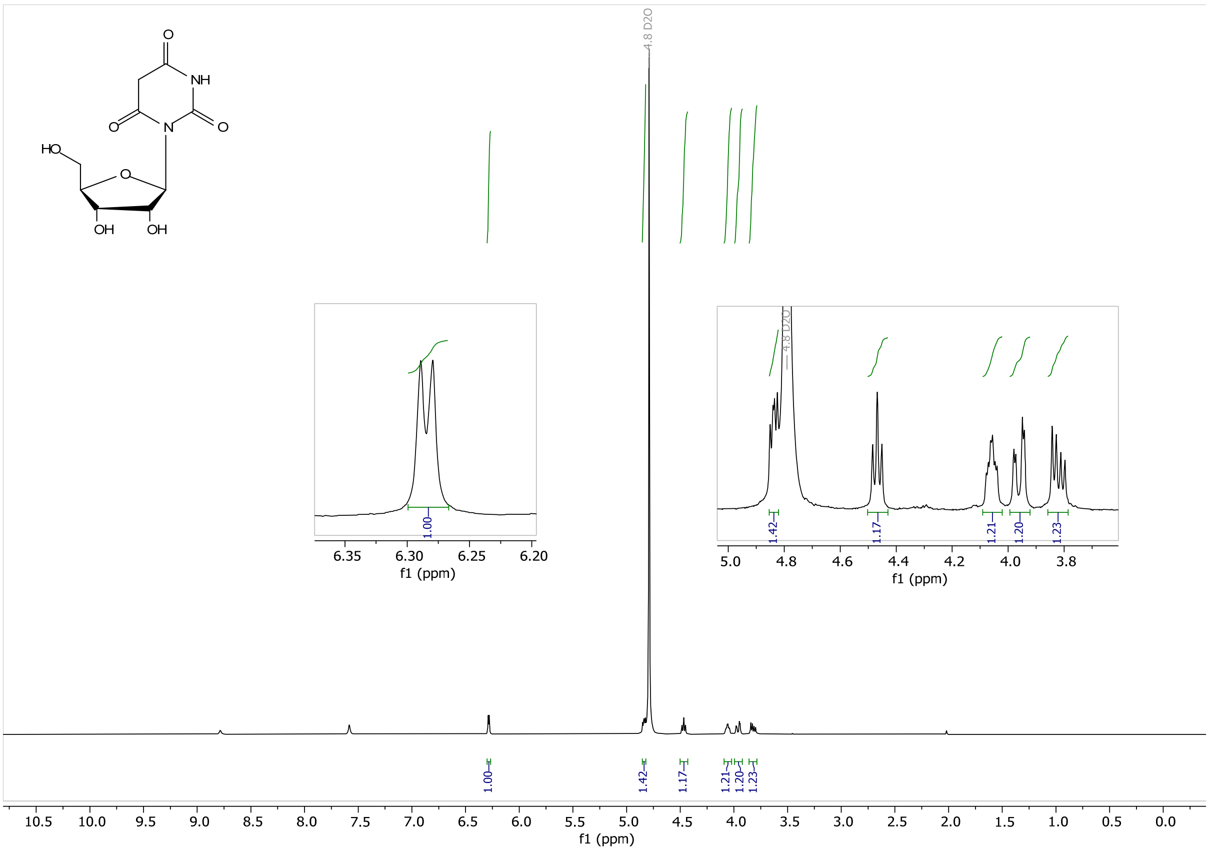

Figure S15. ¹H NMR of 6-Hydroxyuridine (4a) in D₂O. Impurities at 7.6 and 8.8 ppm.

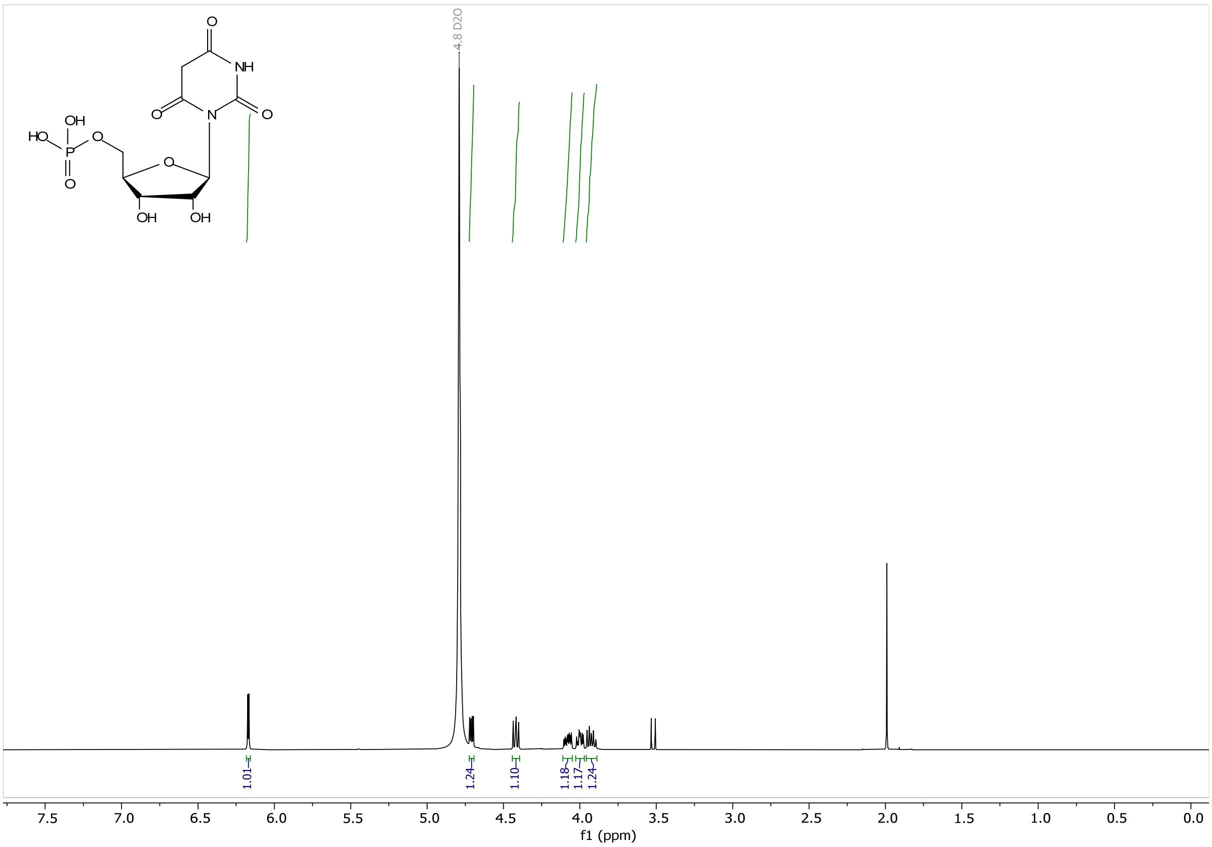

Figure S16. ¹H NMR of BMP in D₂O. Unidentified impurity at 2.0 ppm and trimethyl phosphate signals at 3.5 ppm.

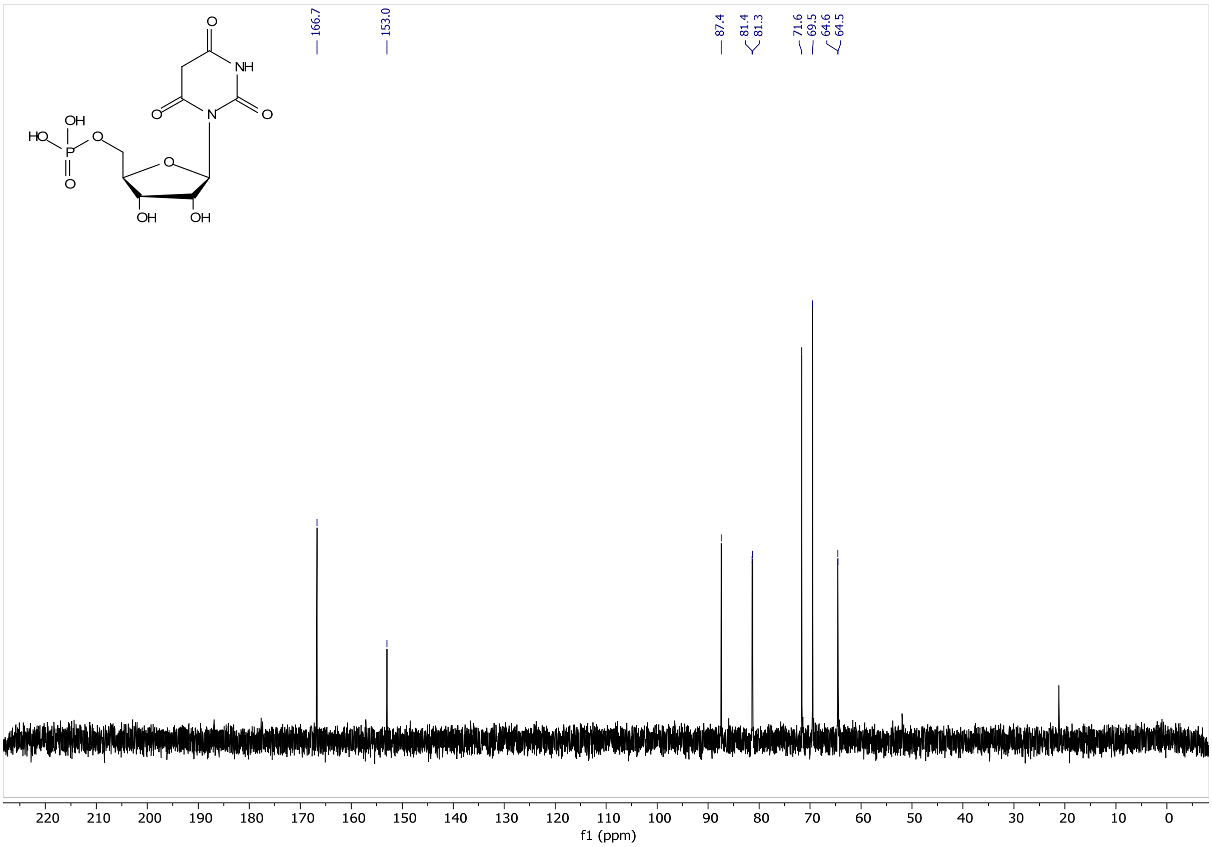

Figure S17. ^{13}C NMR of BMP in D_2O. Unidentified impurity at 21.2 ppm.

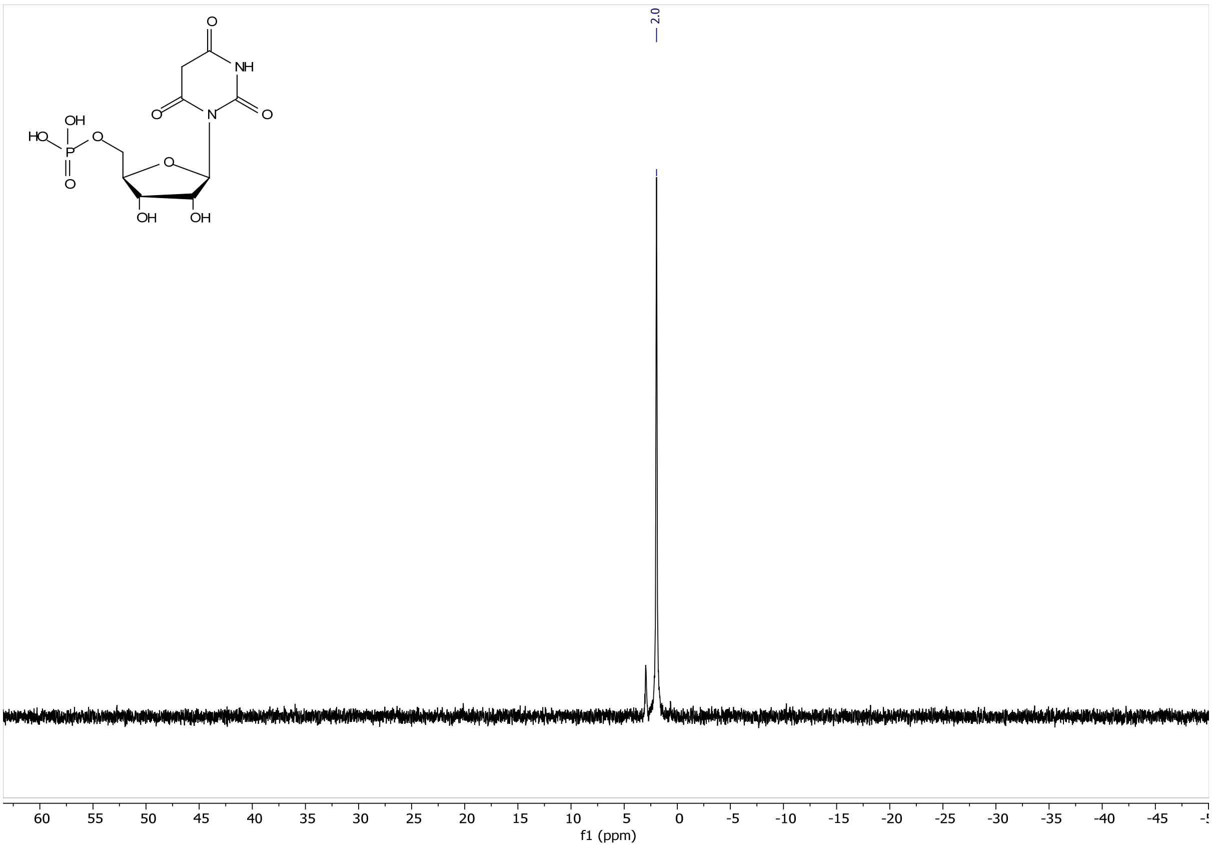

Figure S18. ^{31}P NMR of BMP in D_2O.

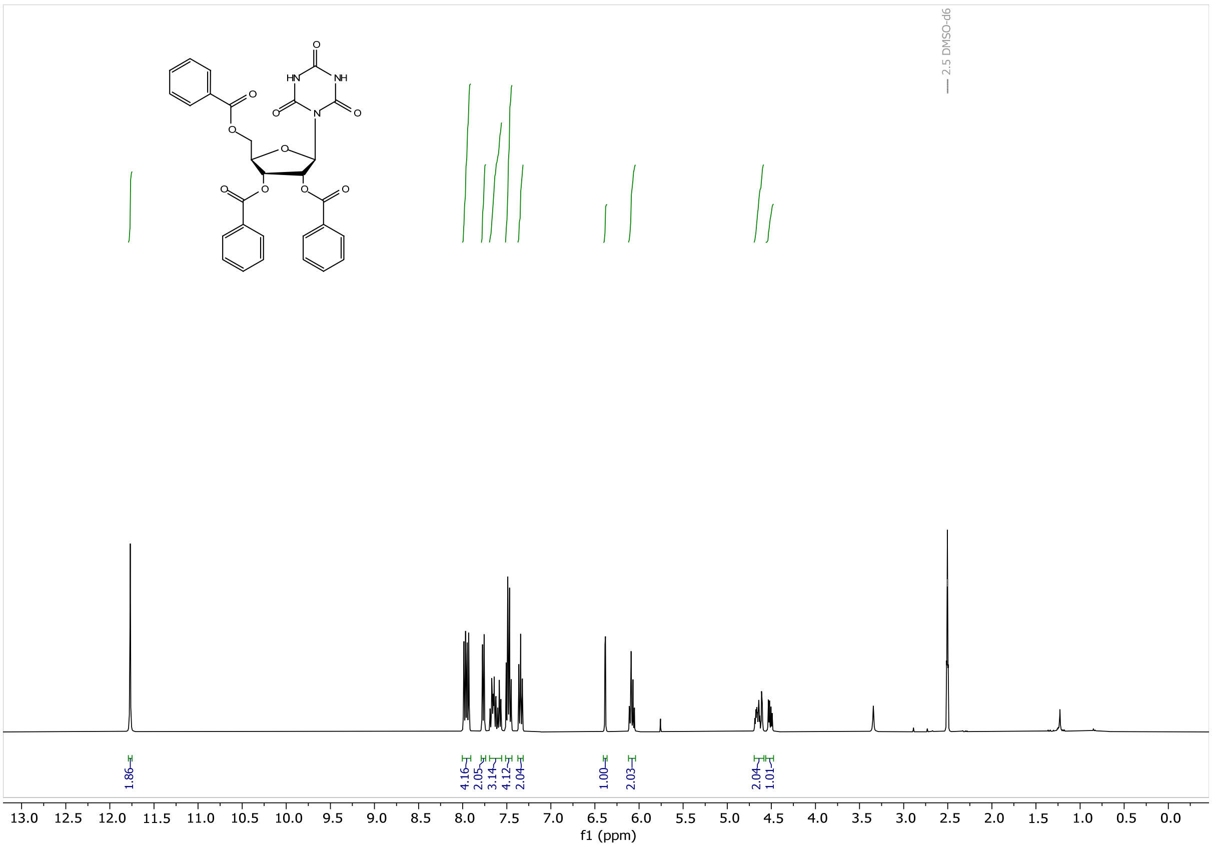

Figure S19. ^{1}**H NMR of β-D-ribofuranose-1-cyanuryl-2,3,5-tribenzoate (2b) in DMSO-*d*$_6$.**

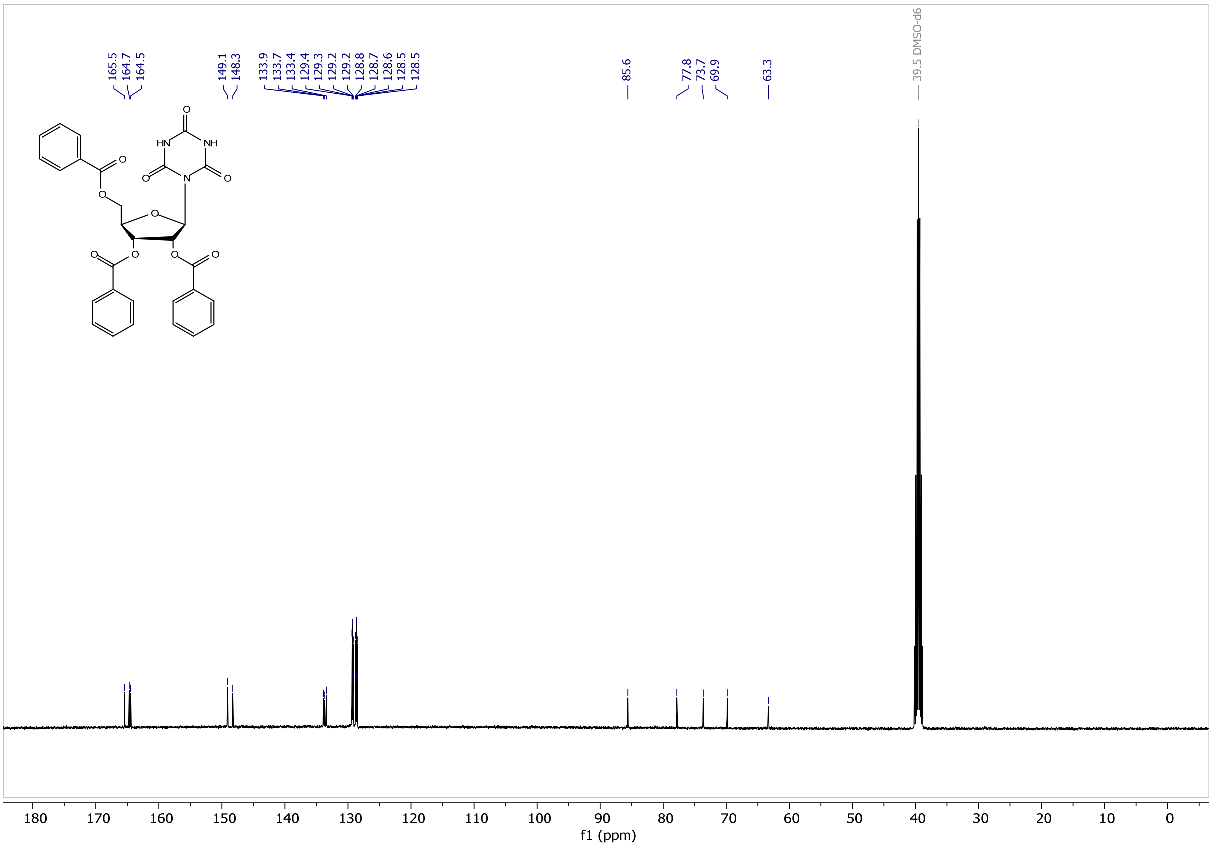

Figure S20. ^{13}**C NMR of β-D-ribofuranose-1-cyanuryl-2,3,5-tribenzoate (2b) in DMSO-*d*$_6$.**

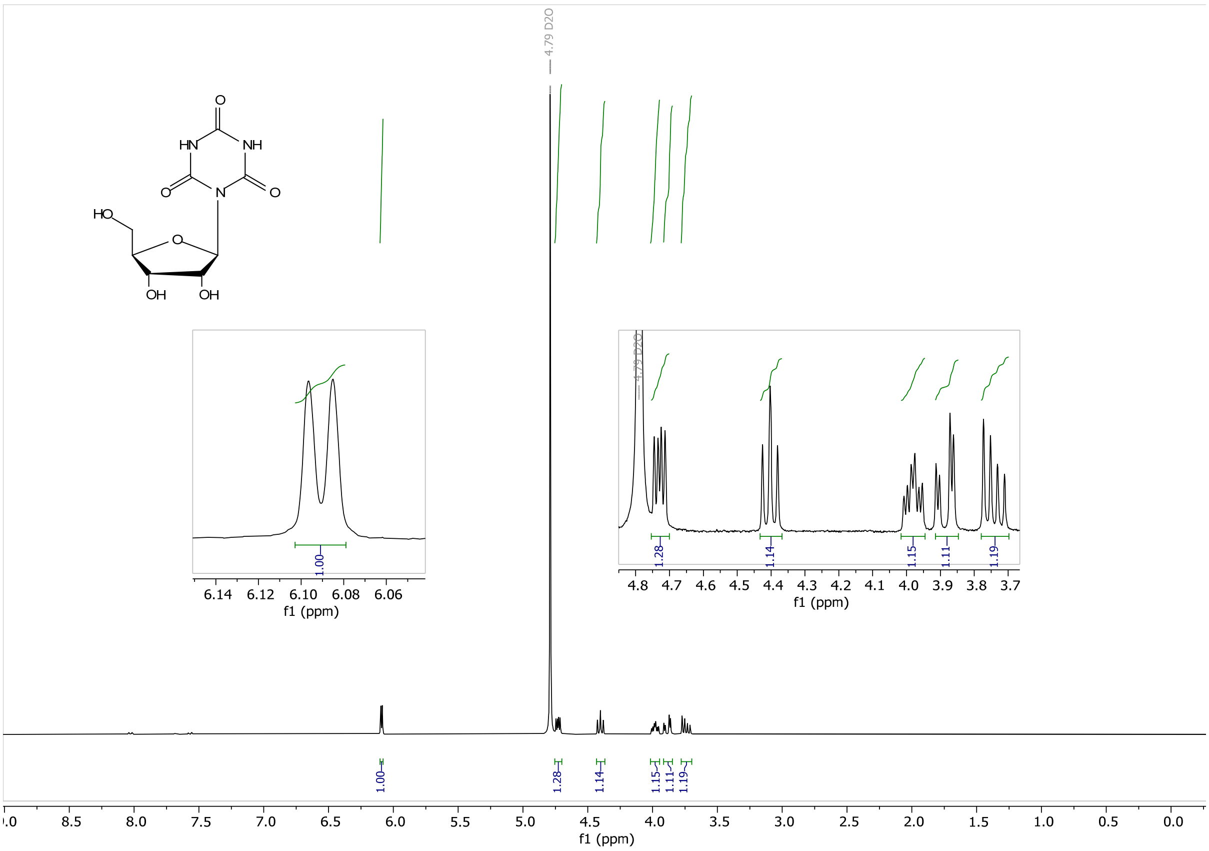

Figure S21. [1]**H NMR of β-cyanuryl-ribose (4b) in** D_2O**.**

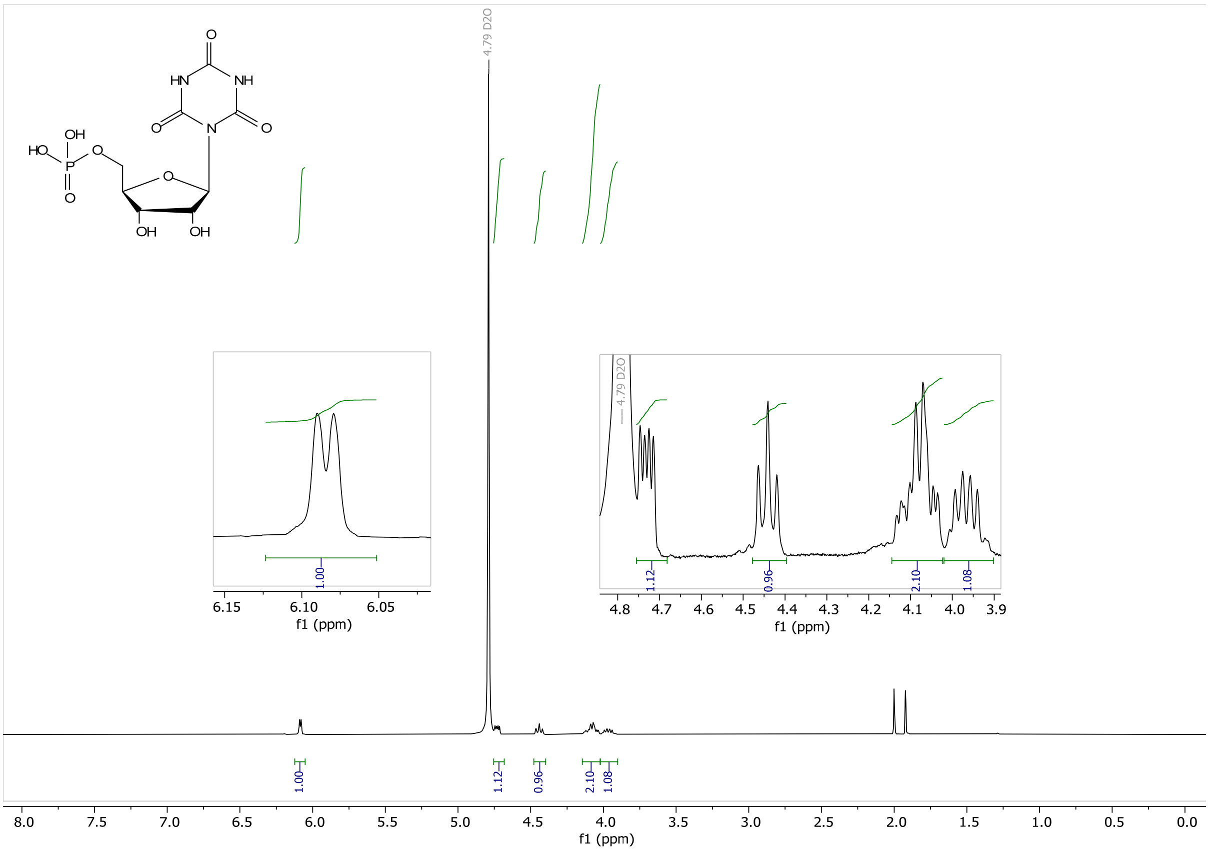

Figure S22. [1]**H NMR of compound YMP in** D_2O**. Unidentified impurity signals at 1.92 and 2.0 ppm.**

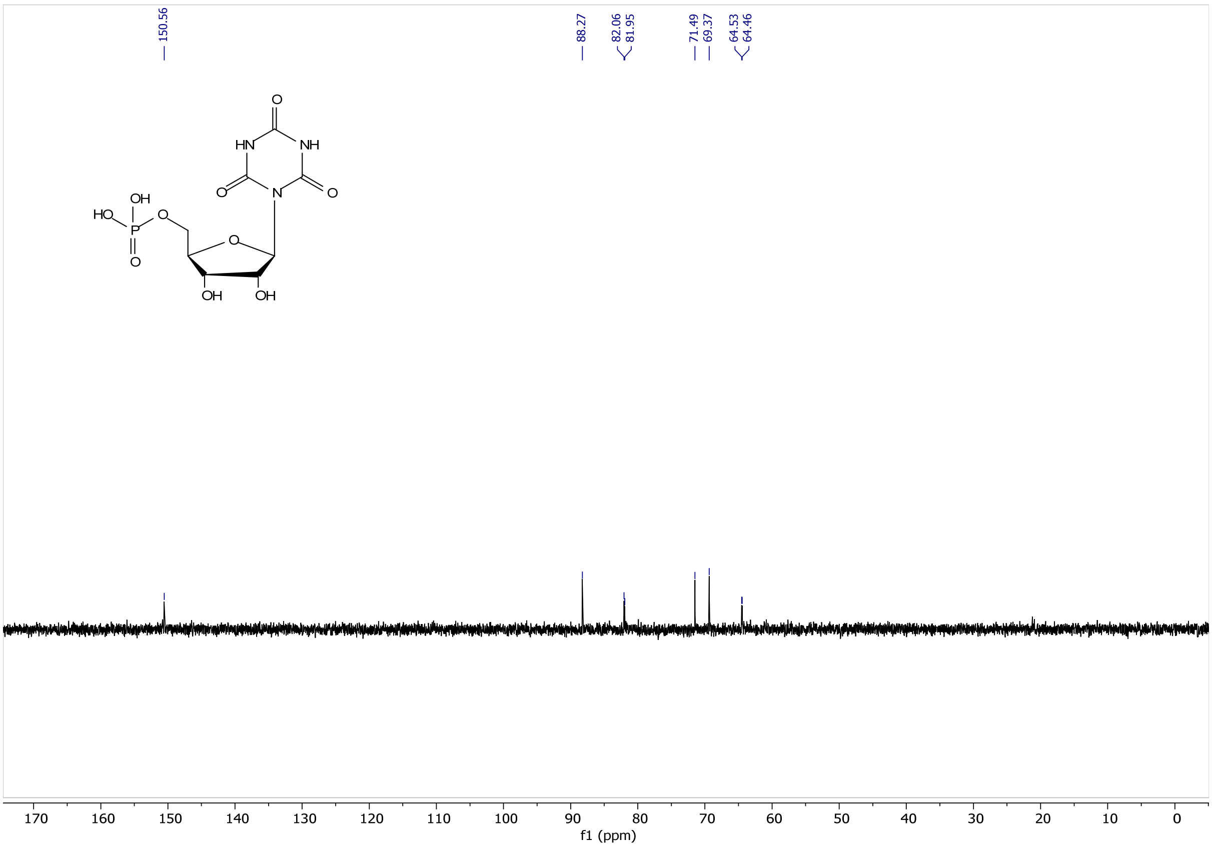

Figure S23. ^{13}**C NMR of compound YMP in** D_2O**.**

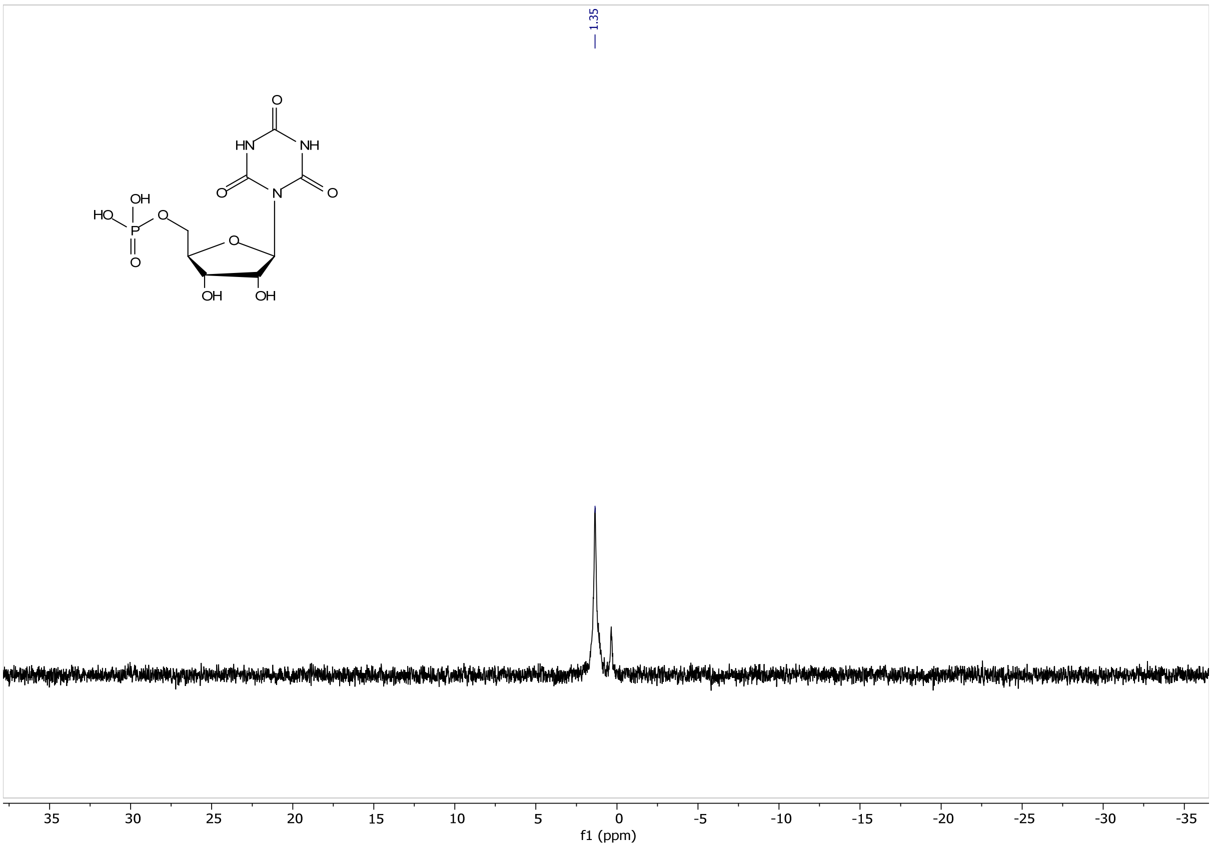

Figure S24. ^{31}**P NMR of compound YMP in** D_2O**.**

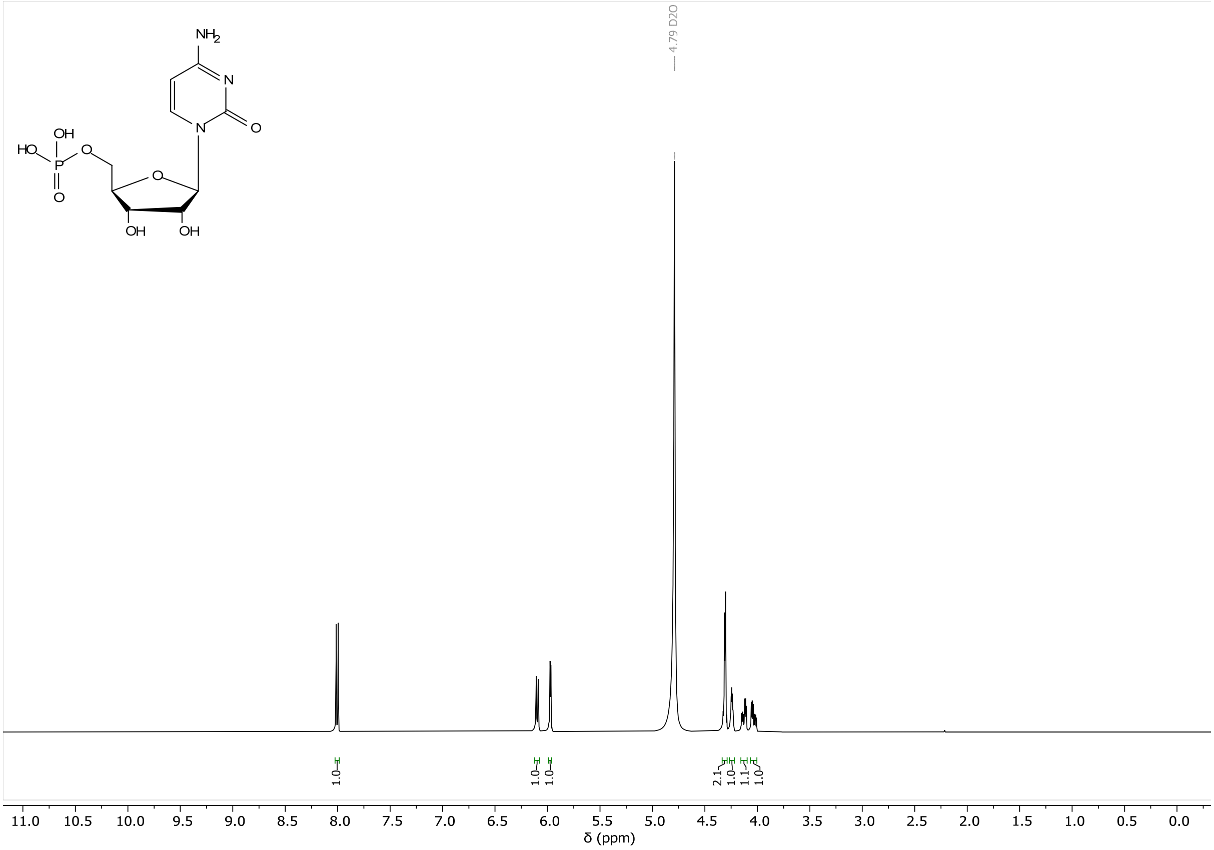

Figure S25. ^{1}**H NMR of CMP in D**$_2$**O.**

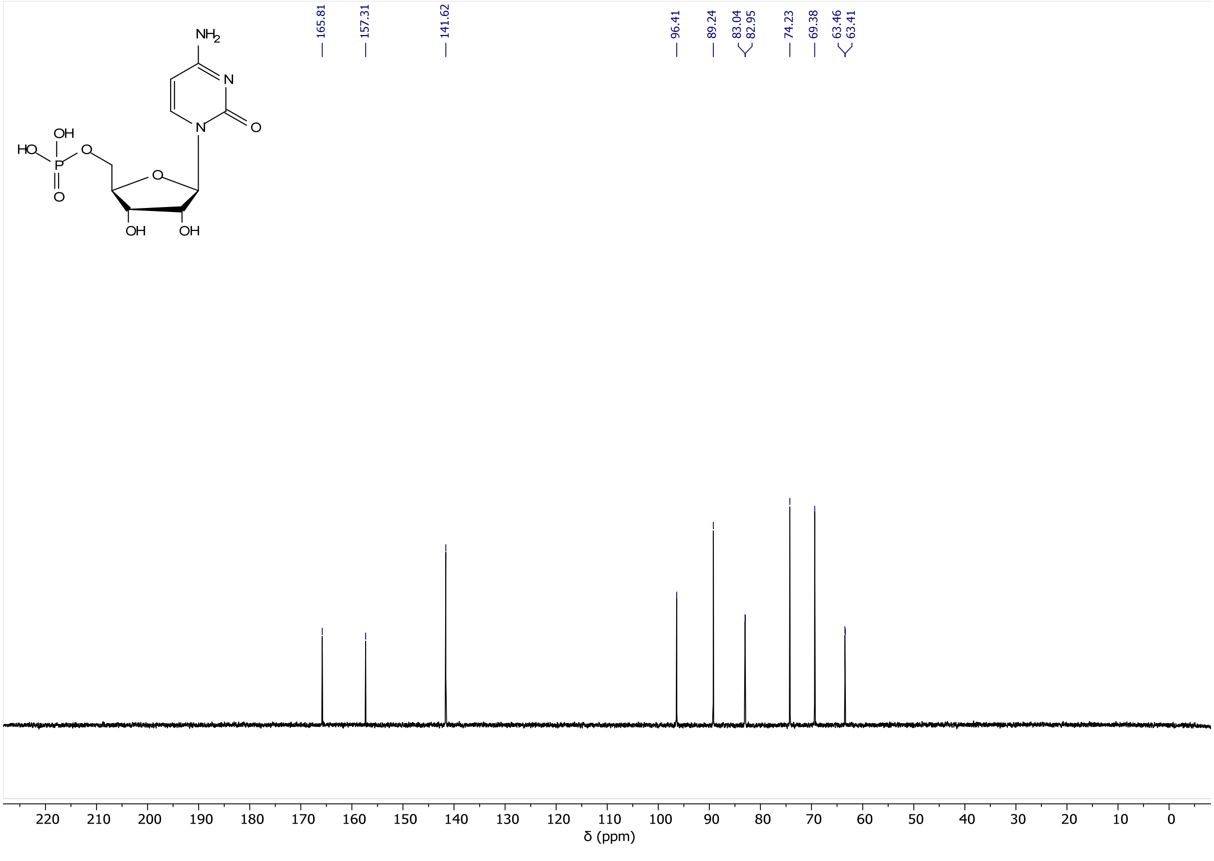

Figure S26. ^{13}**C NMR of CMP in D**$_2$**O.**

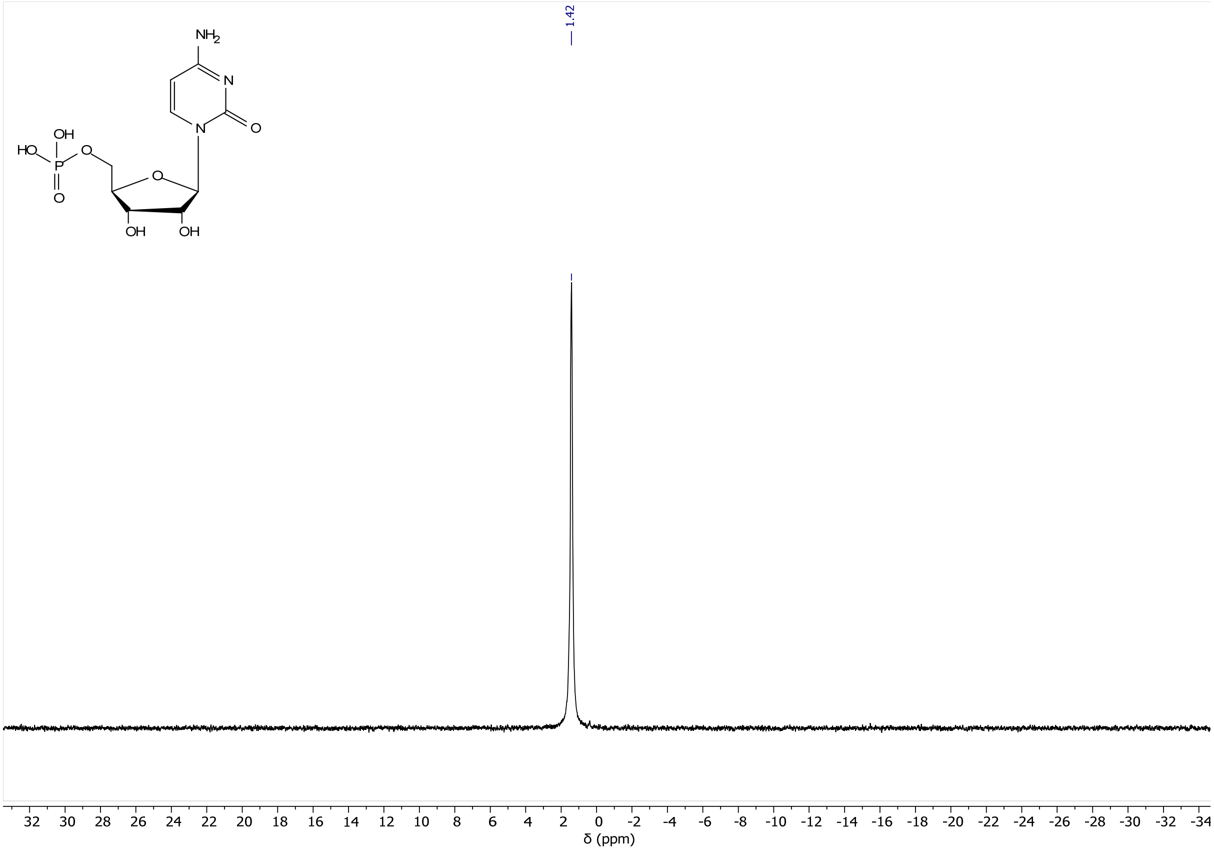

Figure S27. ^{31}P NMR of CMP in D_2O.

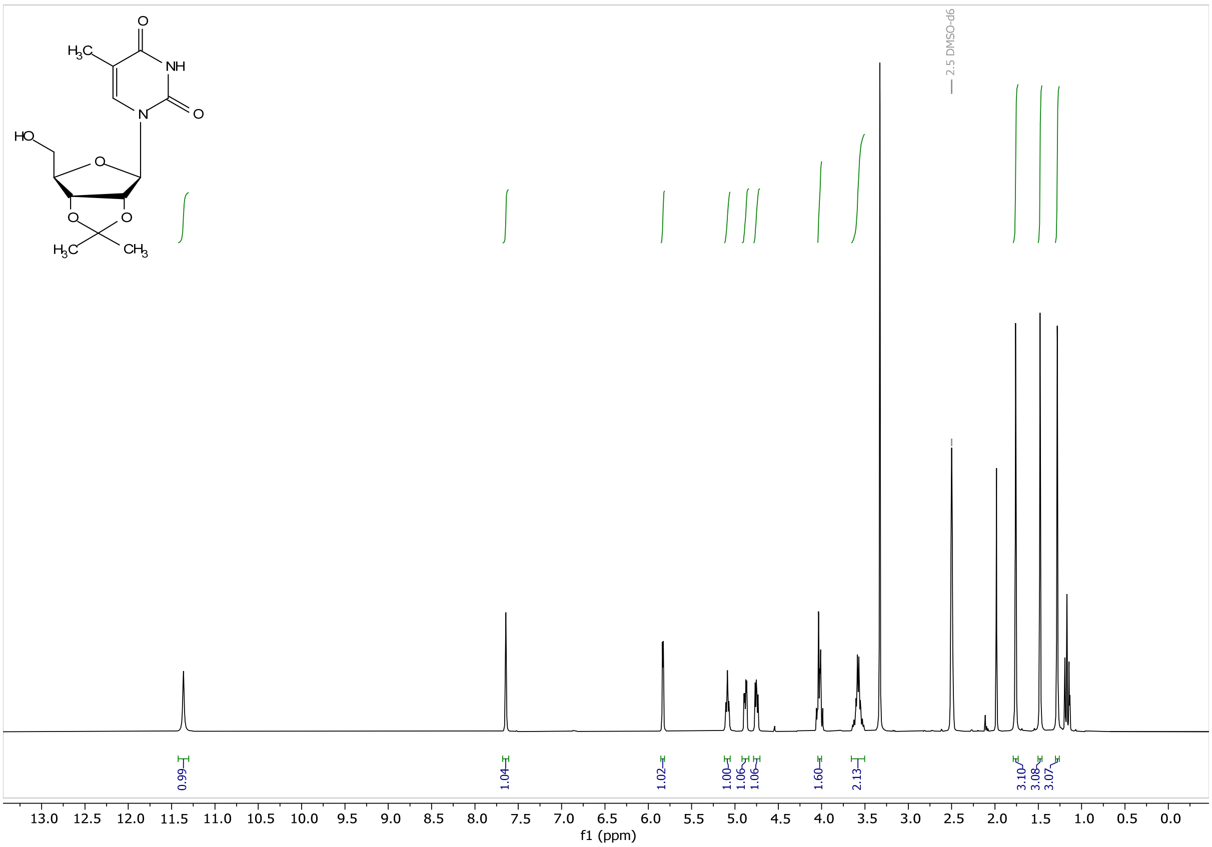

Figure S28. ^{1}H NMR of 5 in DMSO-d_6. Ethyl acetate signals at 1.20, 2.00, and 4.00 ppm.

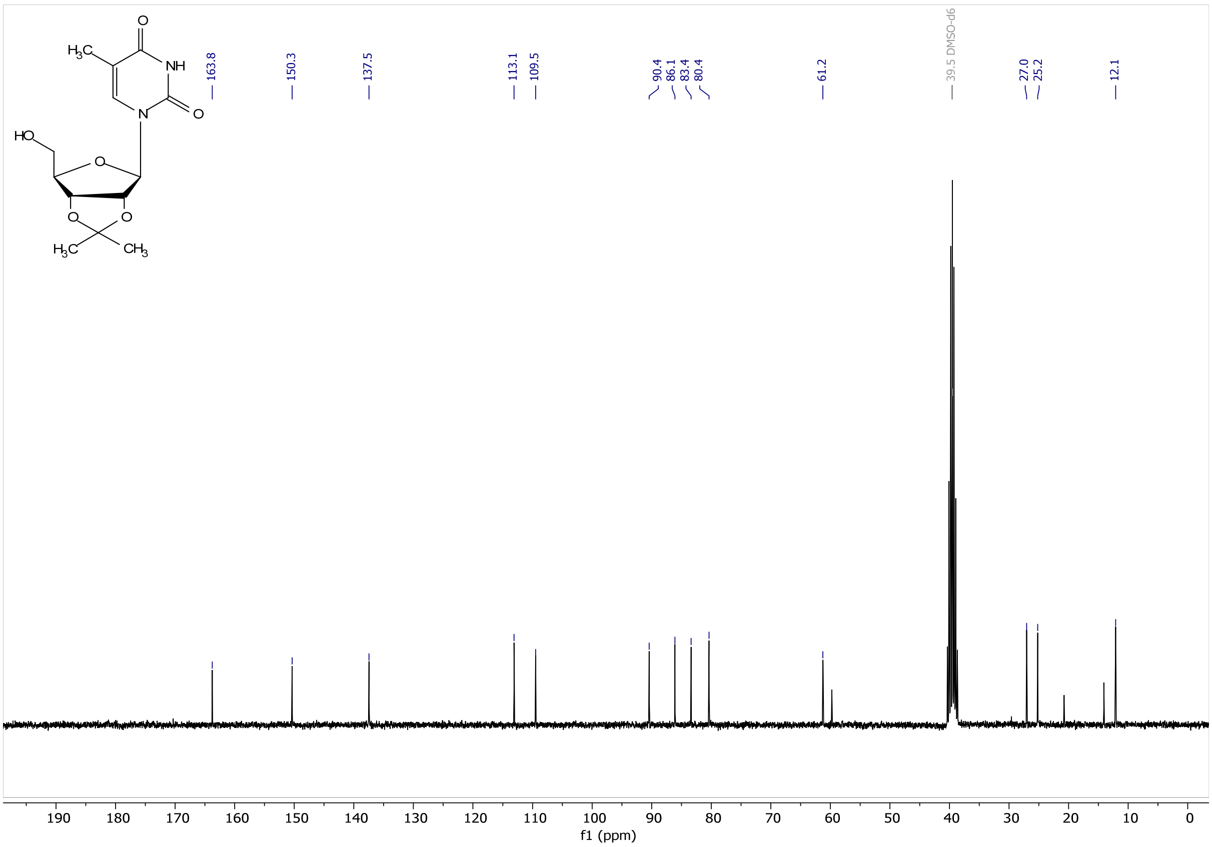

Figure S29. ^{13}C NMR of 5 in DMSO-d_6. Ethyl acetate signals at 14.1, 20.7 and 59.7 ppm.

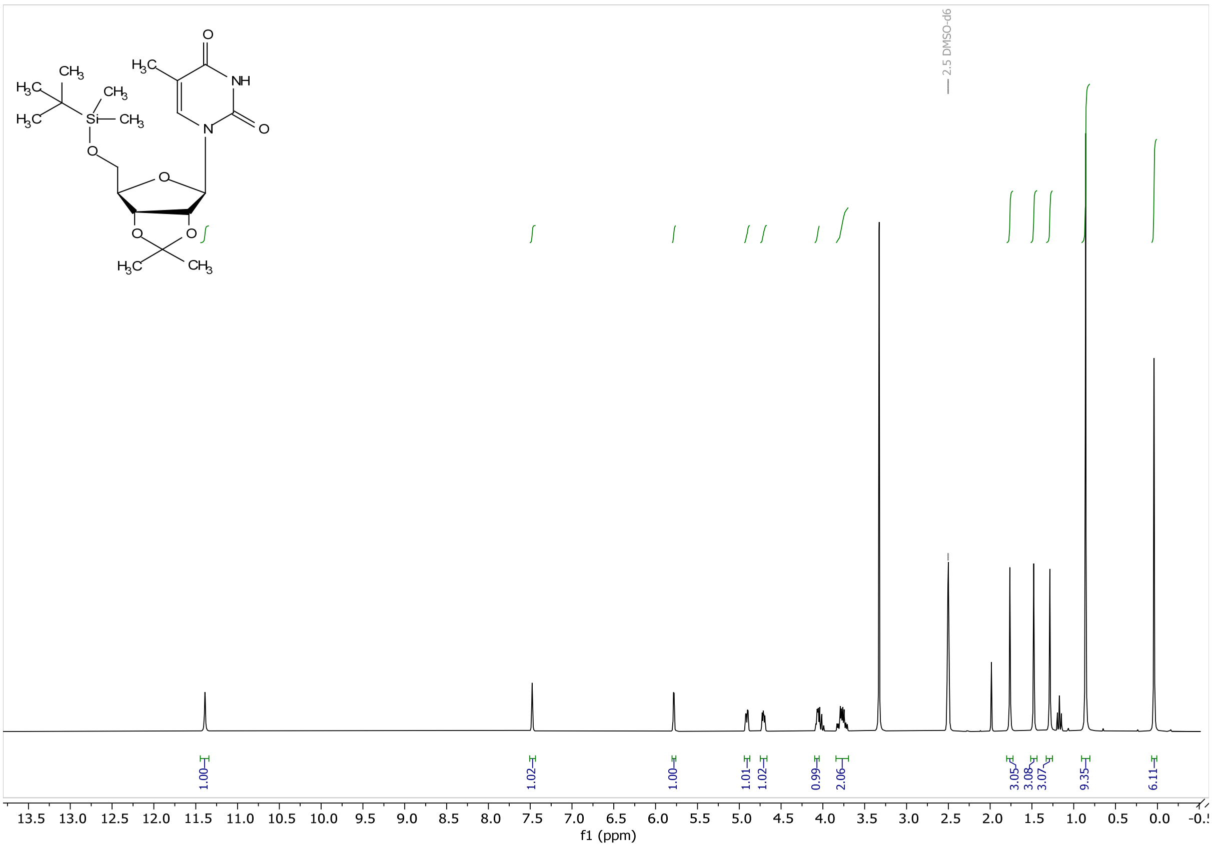

Figure S30. ^{1}H NMR of 6 in DMSO-d_6. Ethyl acetate signals at 1.20, 2.00, and 4.00 ppm.

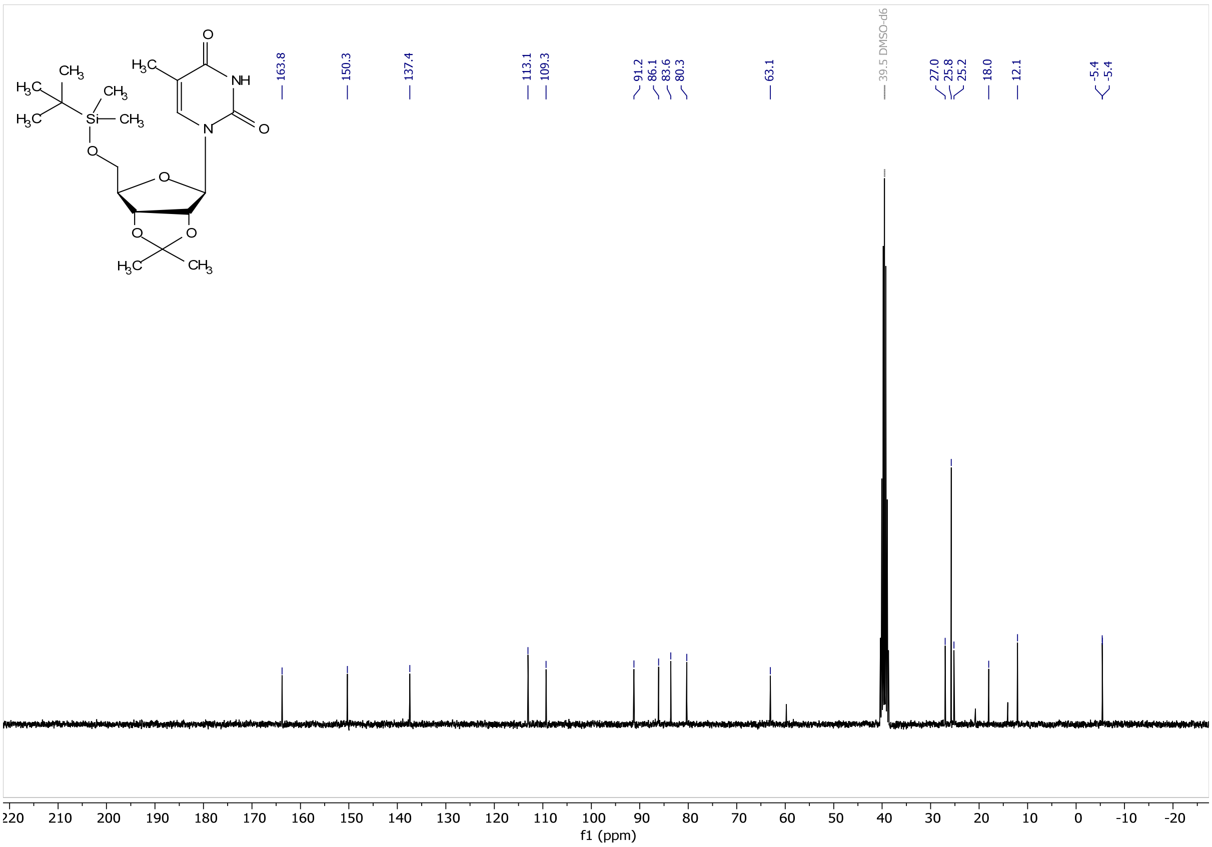

Figure S31. **^{13}C NMR of 6 in DMSO-*d*6. Ethyl acetate signals at 14.1, 20.8 and 59.8 ppm.**

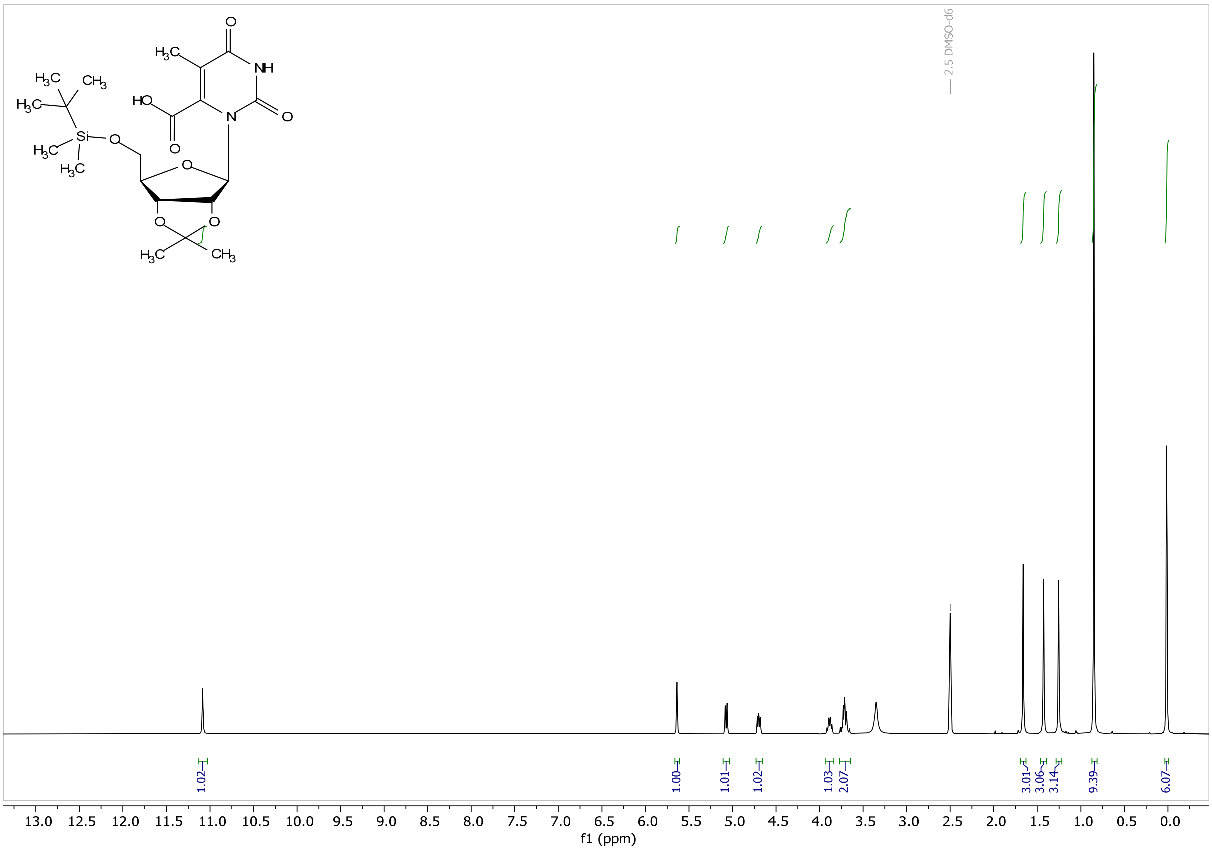

Figure S32. **^{1}H NMR of 7 in DMSO-*d*6.**

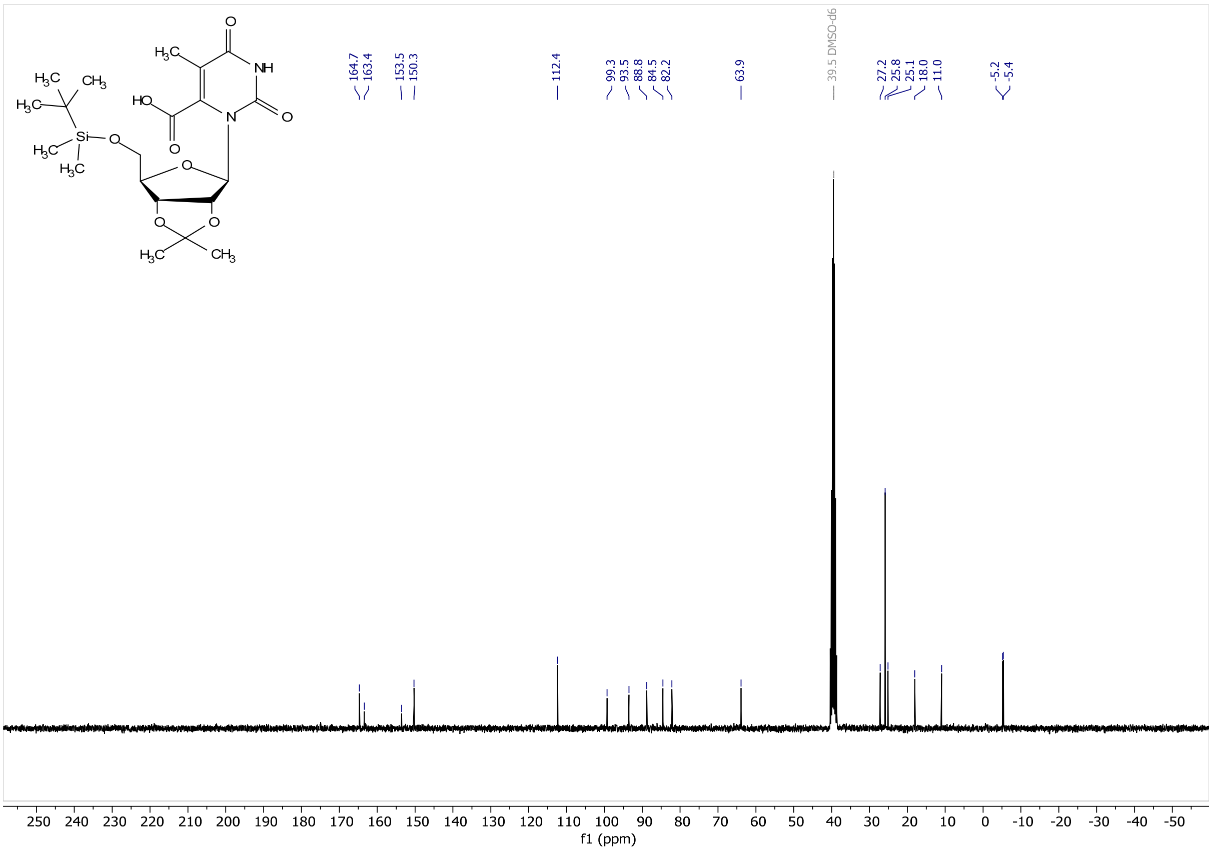

Figure S33. ^{13}**C NMR of 7 in DMSO-**d_6**.**

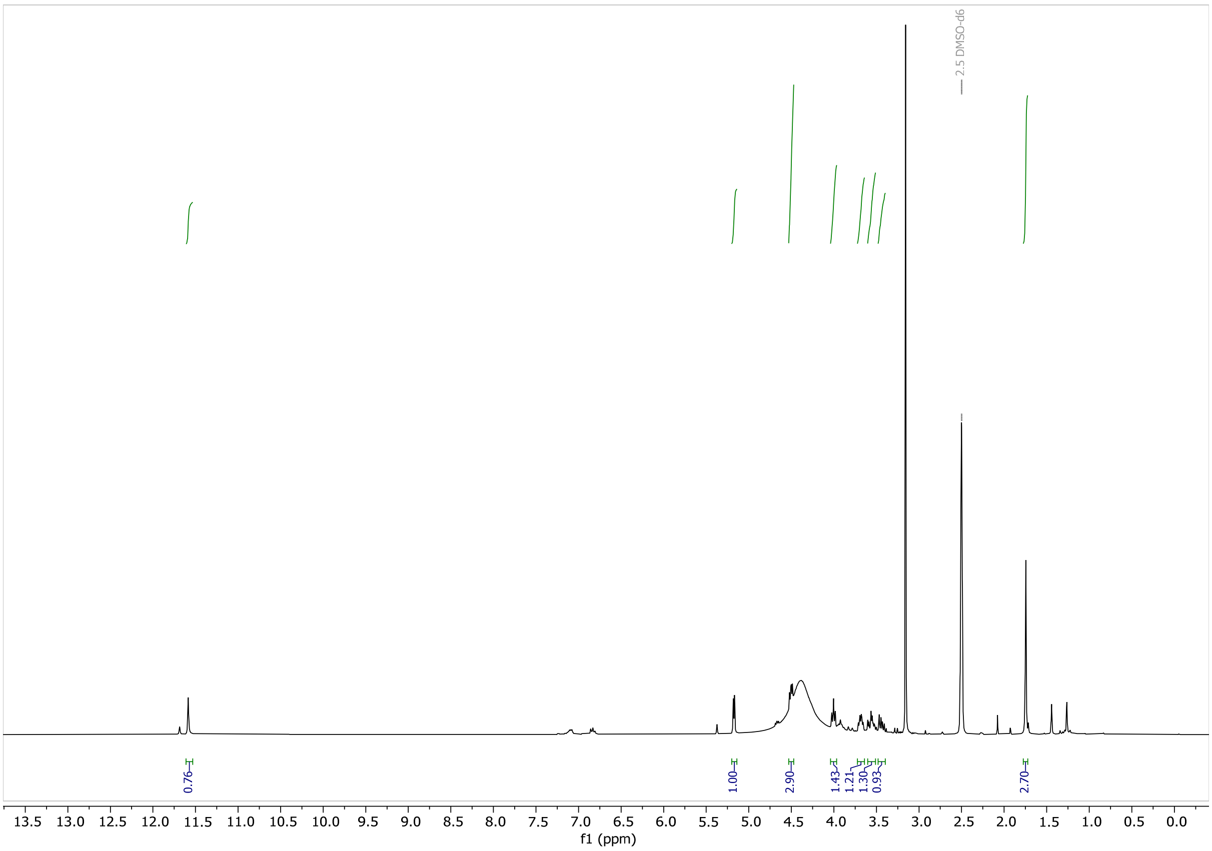

Figure S34. ^{1}**H NMR of 8 in DMSO-**d_6**.**

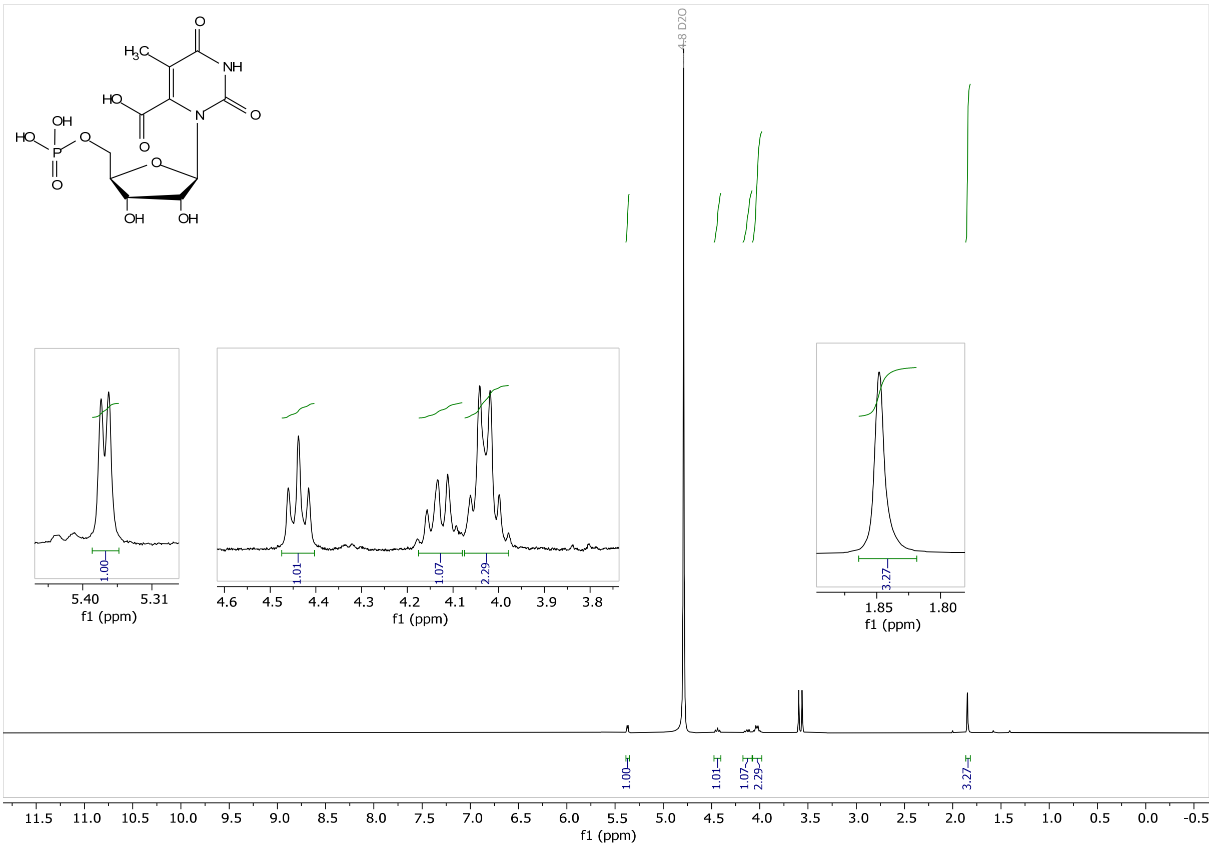

Figure S35. ^{1}H NMR of 5-Methyl OMP in D_2O. Trimethyl phosphate signals at 3.6 ppm.

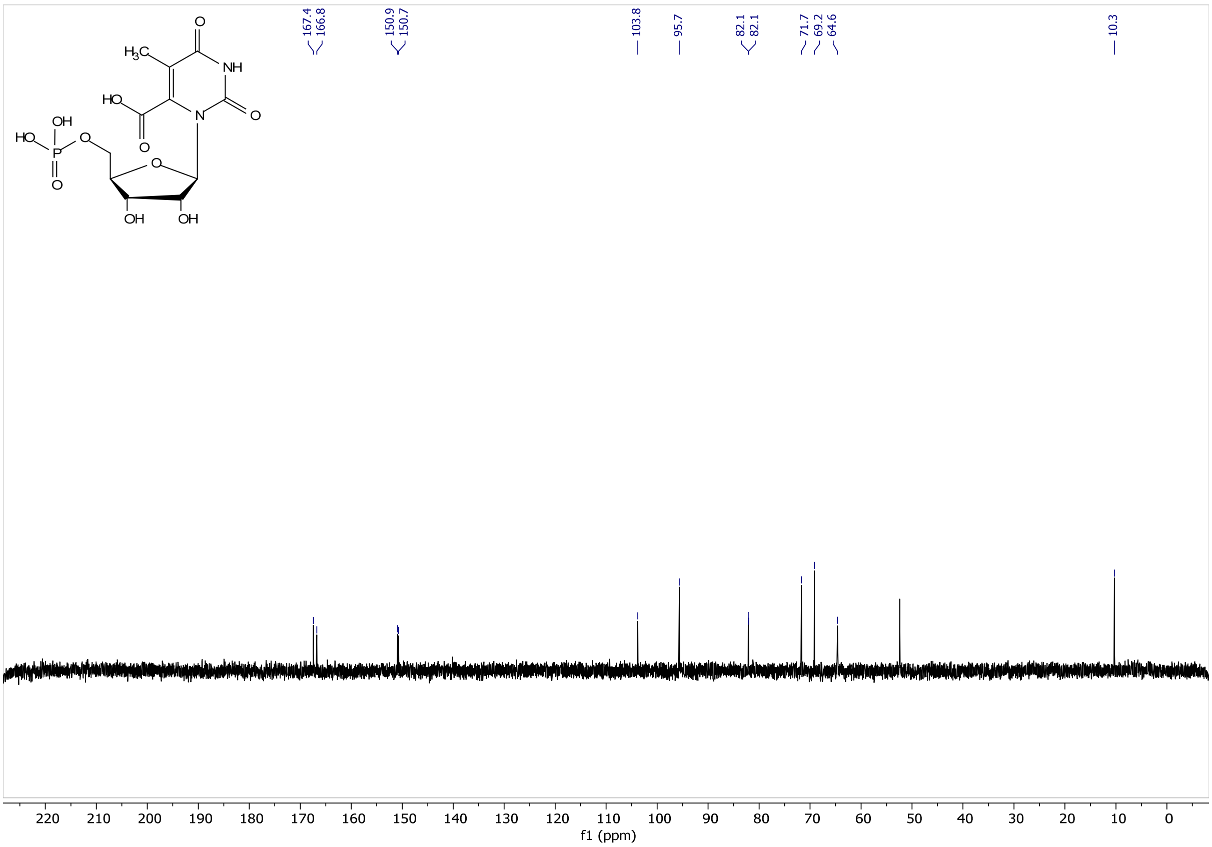

Figure S36. ^{13}C NMR of 5-Methyl OMP in D_2O. Trimethyl phosphate signal at 52.4 ppm.

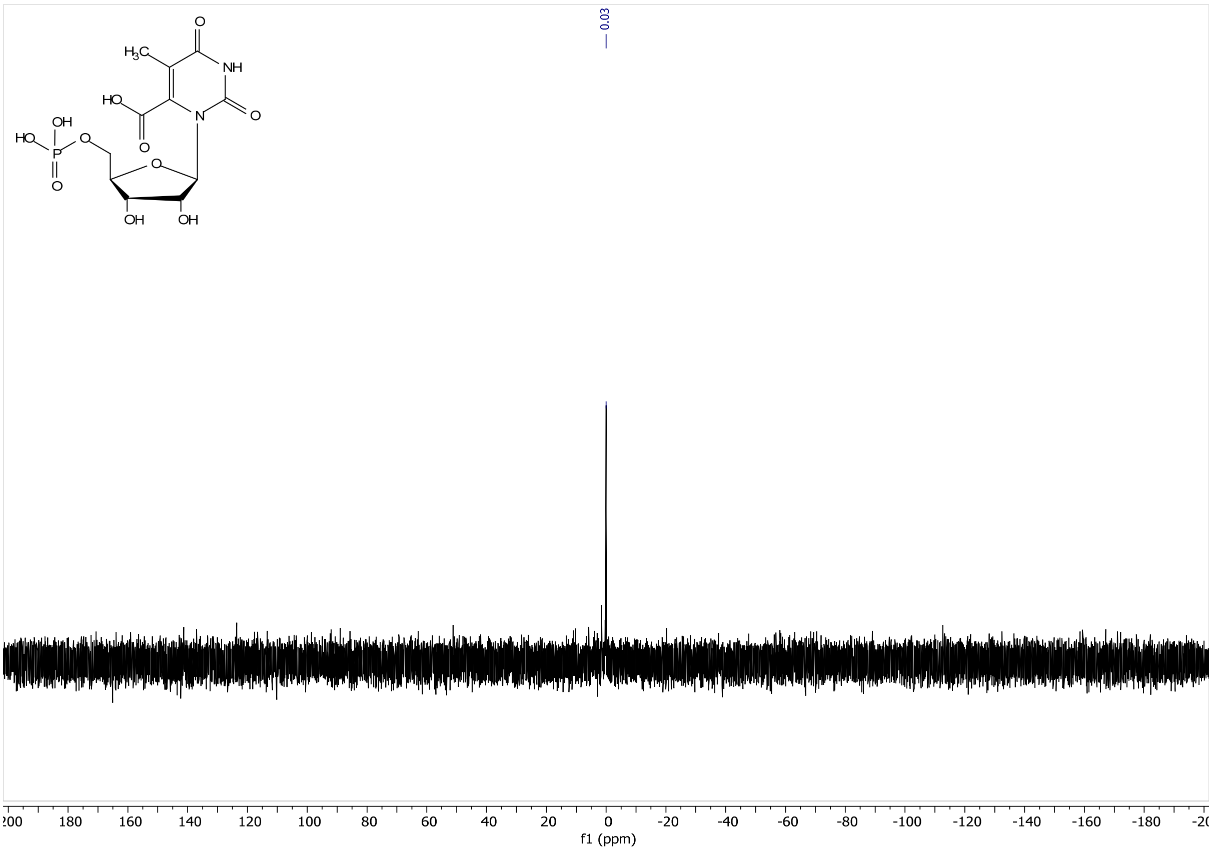

Figure S37. ^{31}P NMR of 5-Methyl OMP in D_2O.

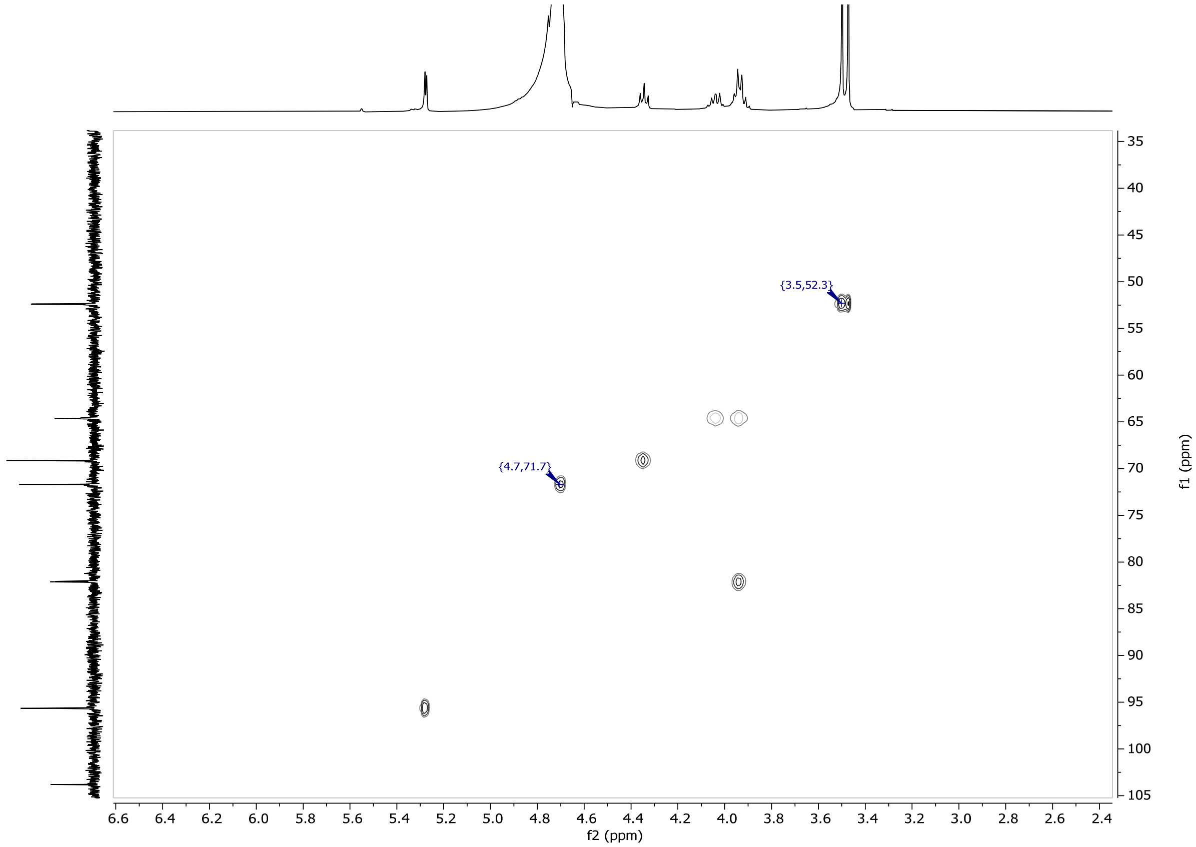

Figure S38. HSQC of 5-Methyl OMP in D_2O. Correlation between 4.7 and 71.7 ppm for the peak under the D_2O water and correlation between 3.6 and 52.3 ppm for trimethyl phosphate.

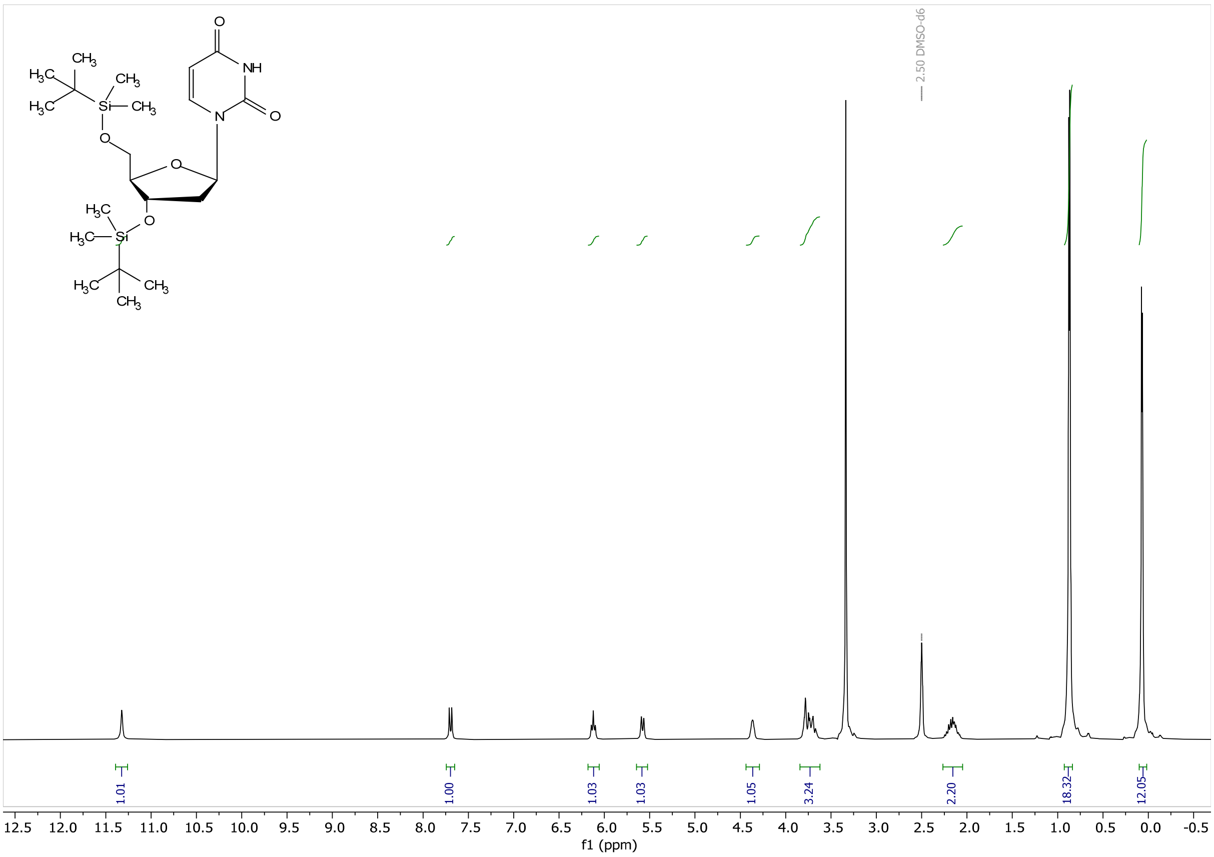

Figure S39. **^{1}H NMR of 9 in DMSO-*d*6.**

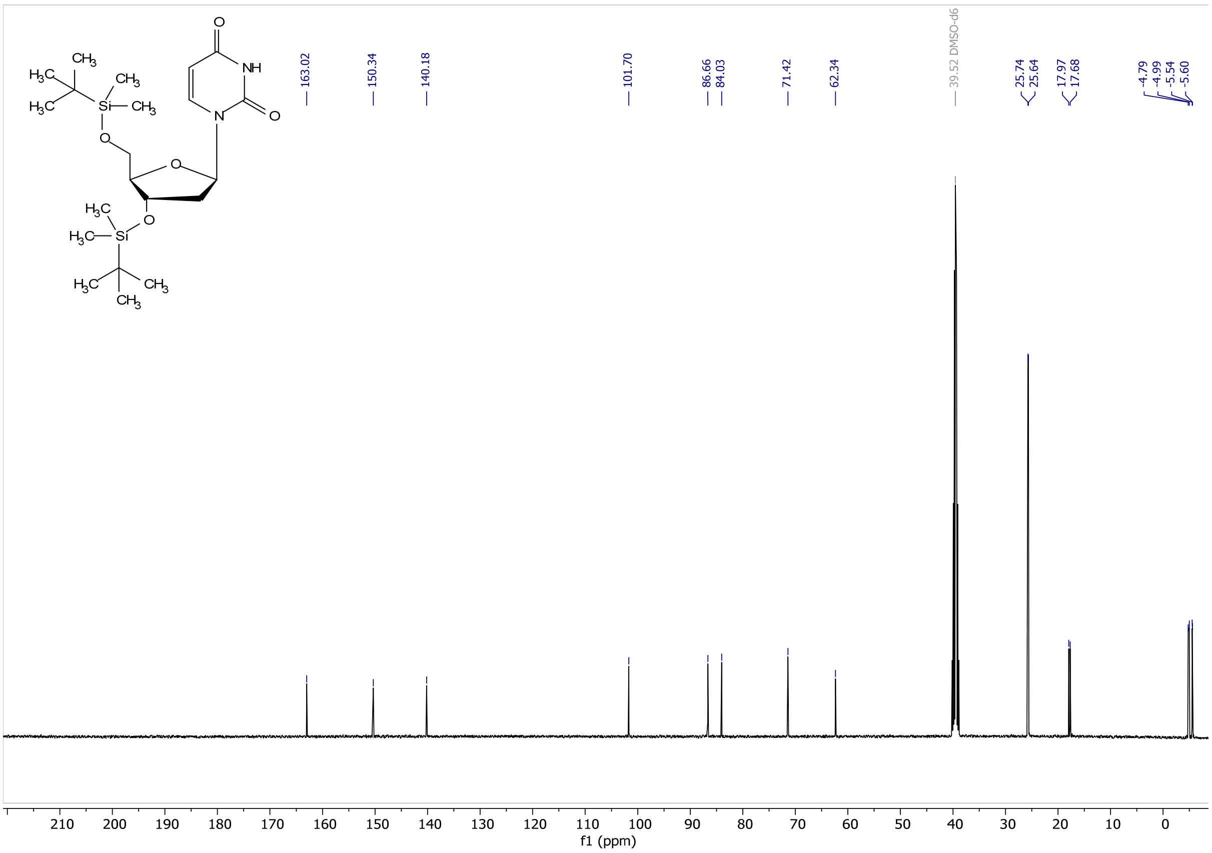

Figure S40. **^{13}C NMR of 9 in DMSO-*d*6.**

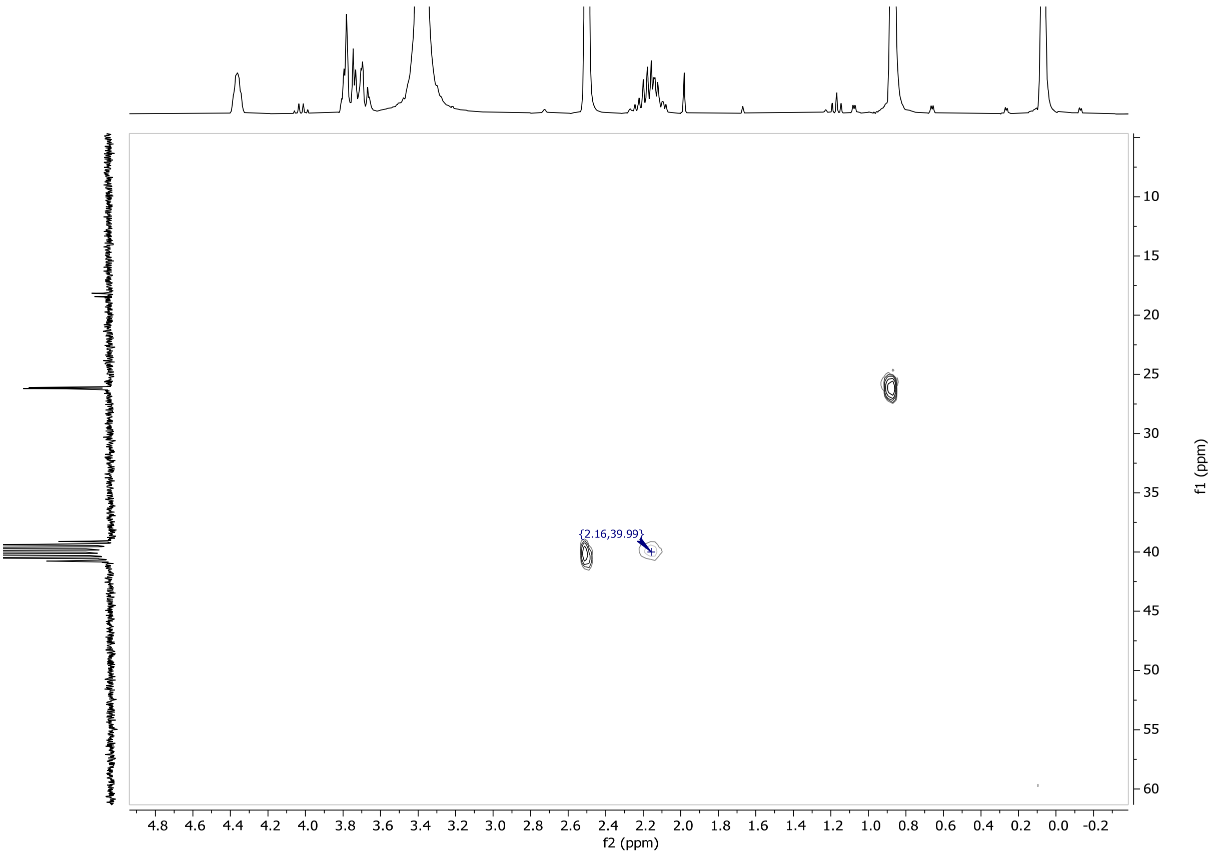

Figure S41. HSQC of 9 in DMSO-d_6. Correlation between 2.16 and 40.0 ppm for 2'-CH_2.

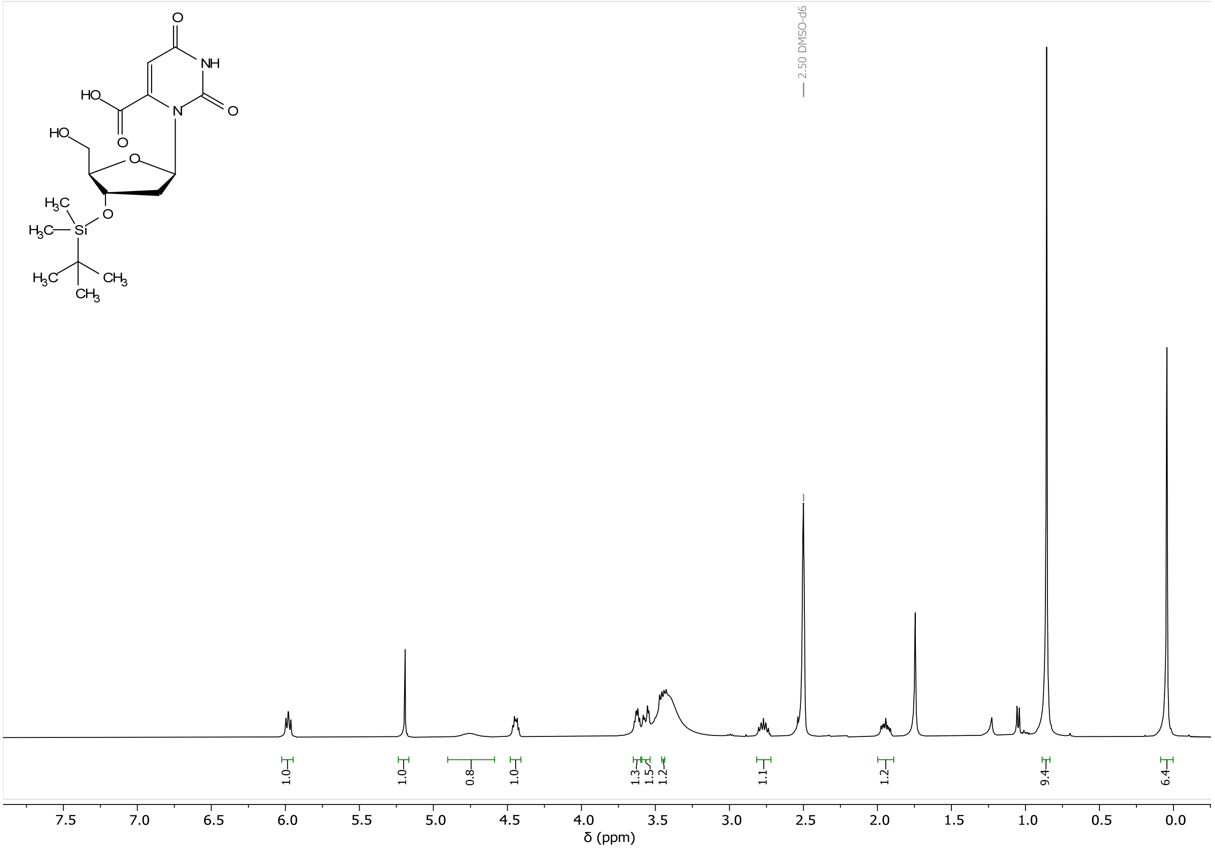

Figure S42. ^{1}H NMR of 10 in DMSO-d_6. Unidentified signal at 1.75 ppm.

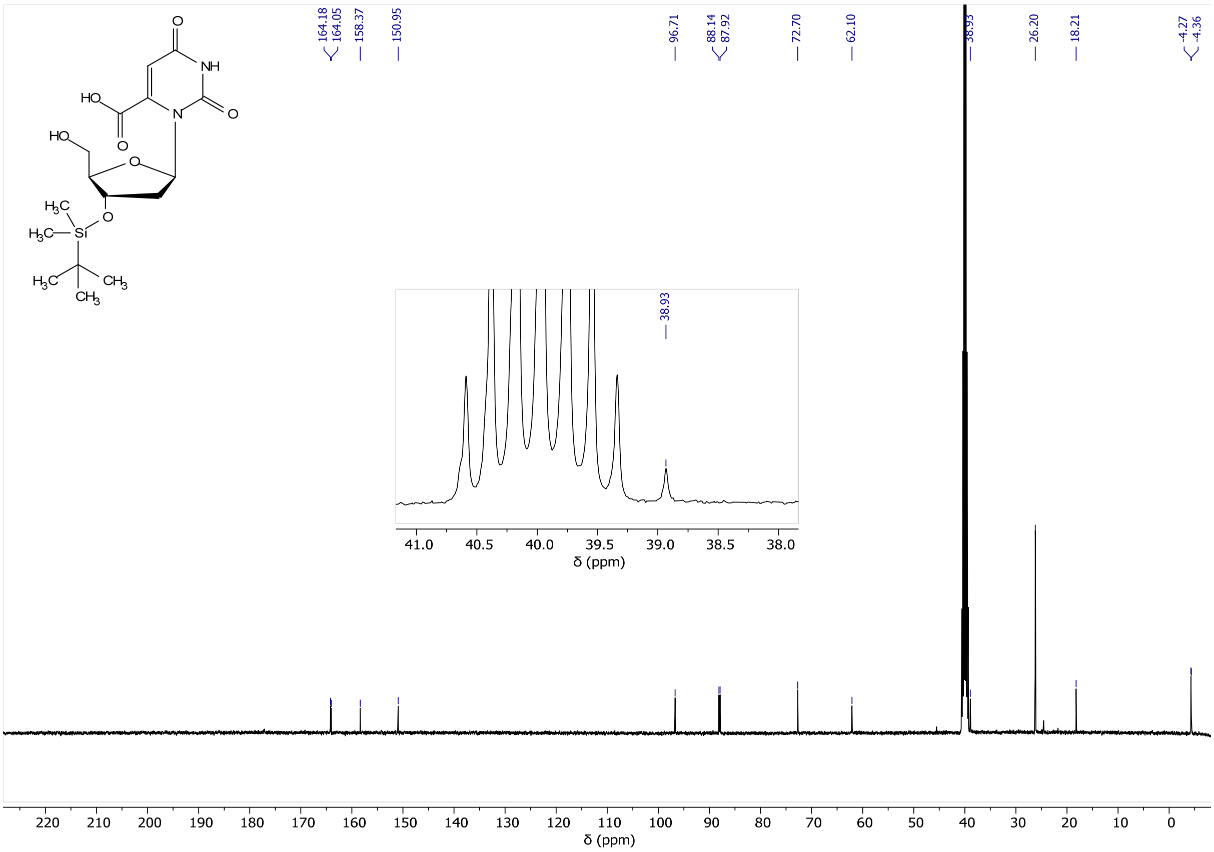

Figure S43. ^{13}C NMR of 10 in DMSO-d_6.

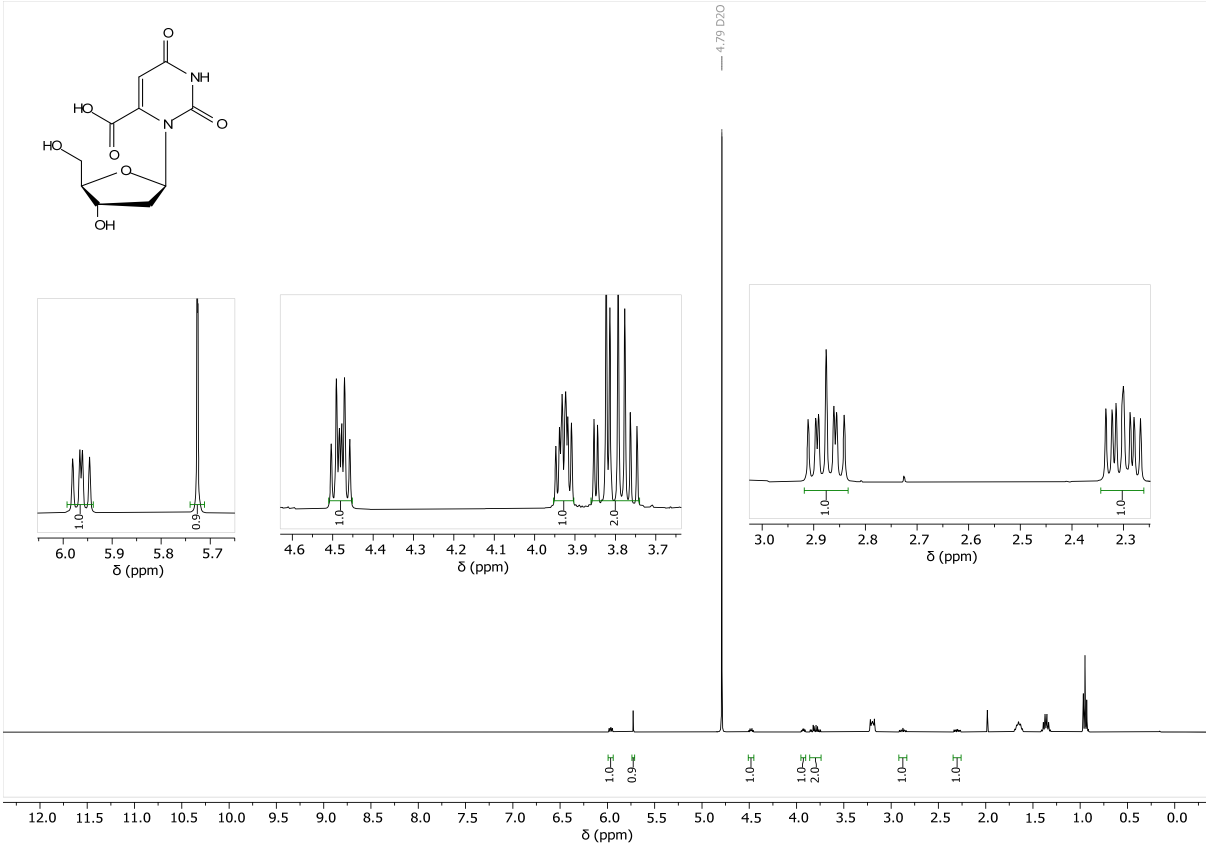

Figure S44. ^{1}H NMR of 11 in D_2O. TBAF peaks at 0.95, 1.36, 1.65, and 3.20 ppm.

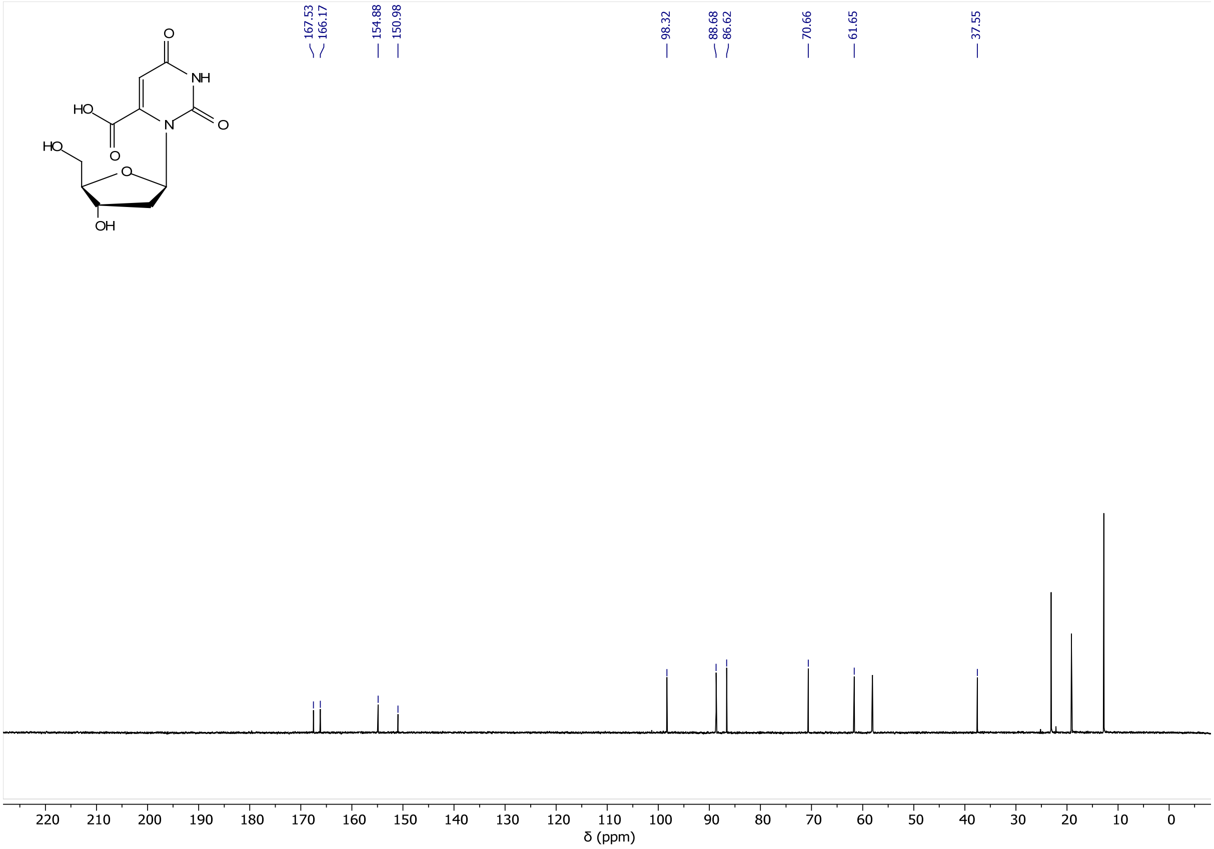

Figure S45. ^{13}**C NMR of 11 in** D_2O**. TBAF peaks at 12.8, 19.1, 23.1, and 58.1 ppm.**

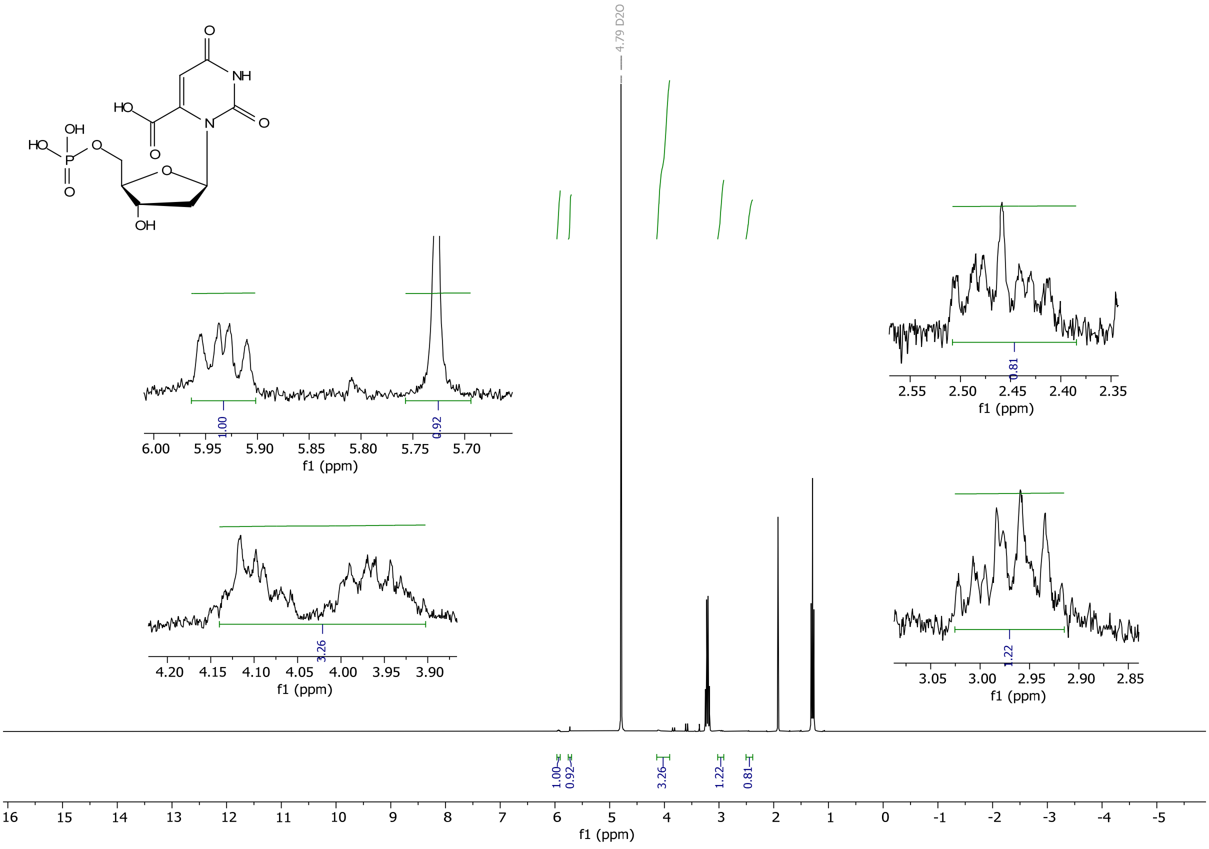

Figure S46. ^{1}**H NMR of Deoxy OMP in** D_2O**. TEAA buffer at 1.29 and 3.21 ppm. Trimethyl phosphate traces at 3.50 ppm and unidentified impurity at 1.92 ppm.**

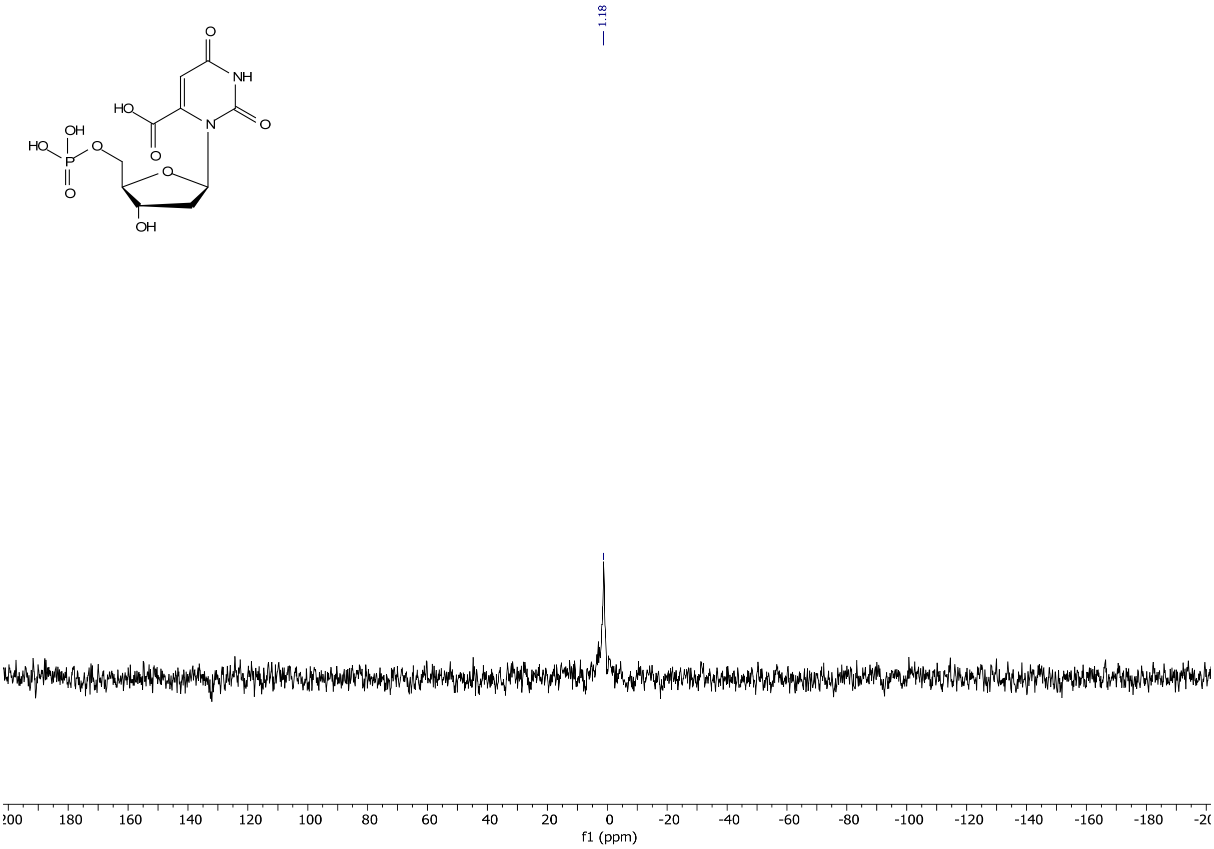

Figure S47. ^{31}P NMR of Deoxy OMP in D_2O.

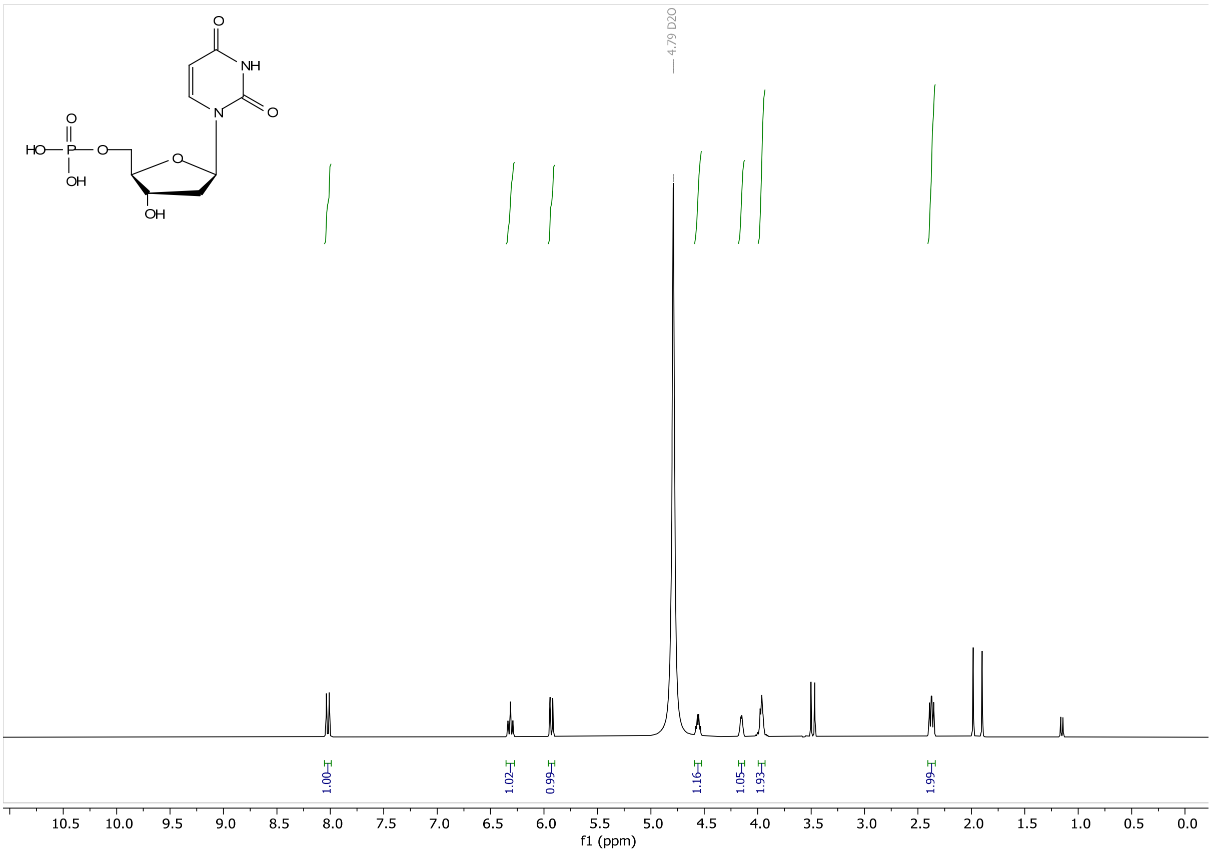

Figure S48. ^{1}H NMR of deoxy UMP in D_2O. Unidentified impurities at 1.95 and 1.15 ppm. Trimethyl phosphate traces at 3.50 ppm.

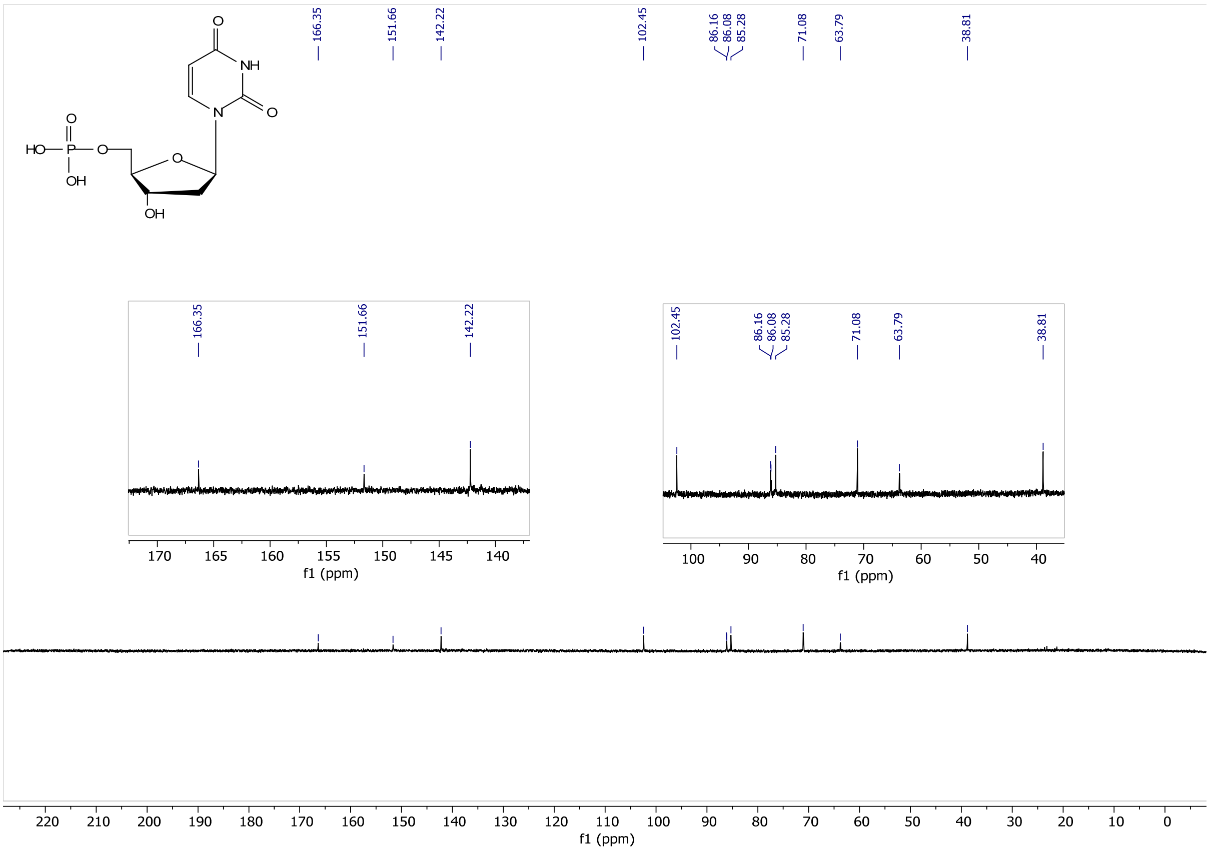

Figure S49. ^{13}C **NMR of deoxy UMP in** D_2O**. Unidentified impurities at 23.2 and 21.2 ppm.**

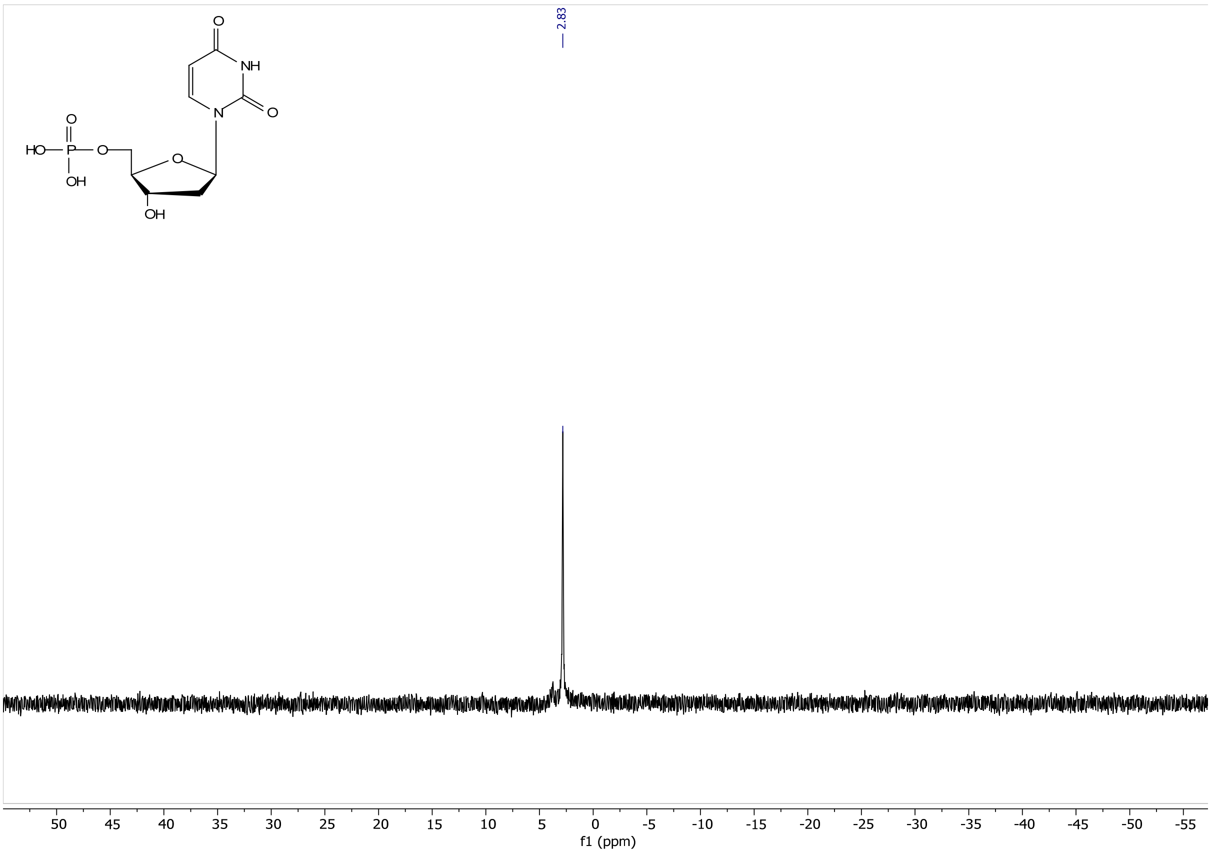

Figure S50. ^{31}P **NMR of deoxy UMP in** D_2O**.**

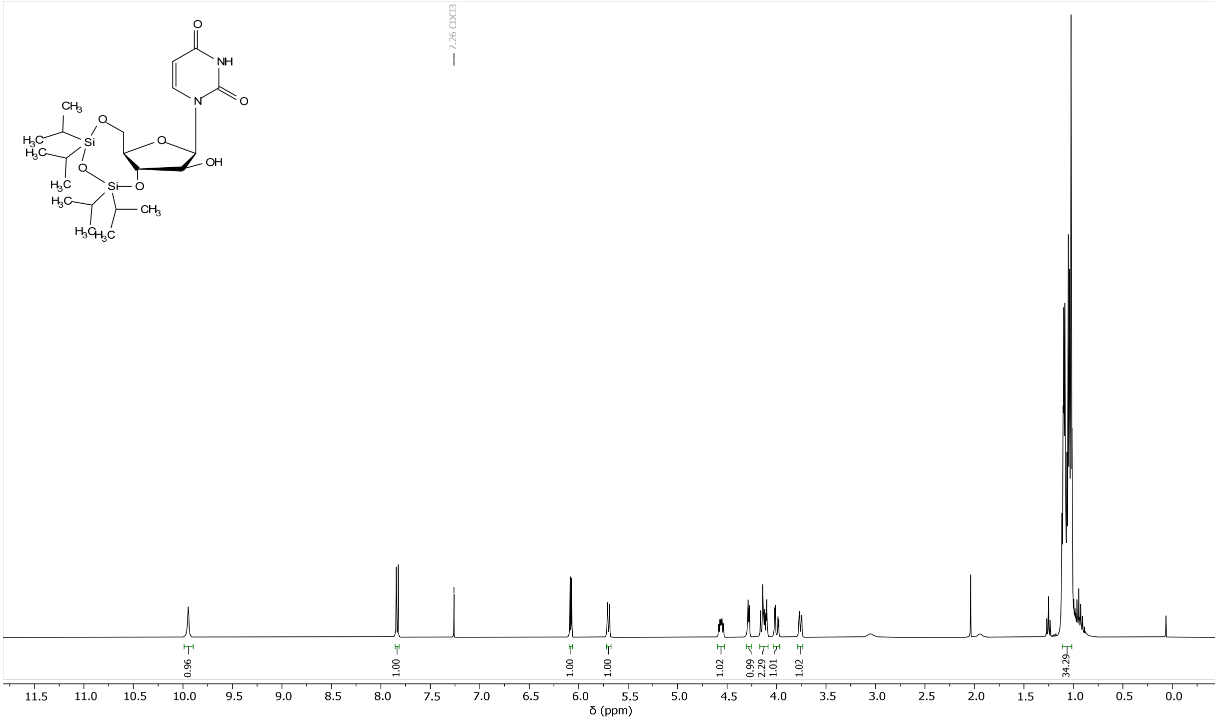

Figure S51. ^{1}**H NMR of 12 in CDCl**$_3$**.**

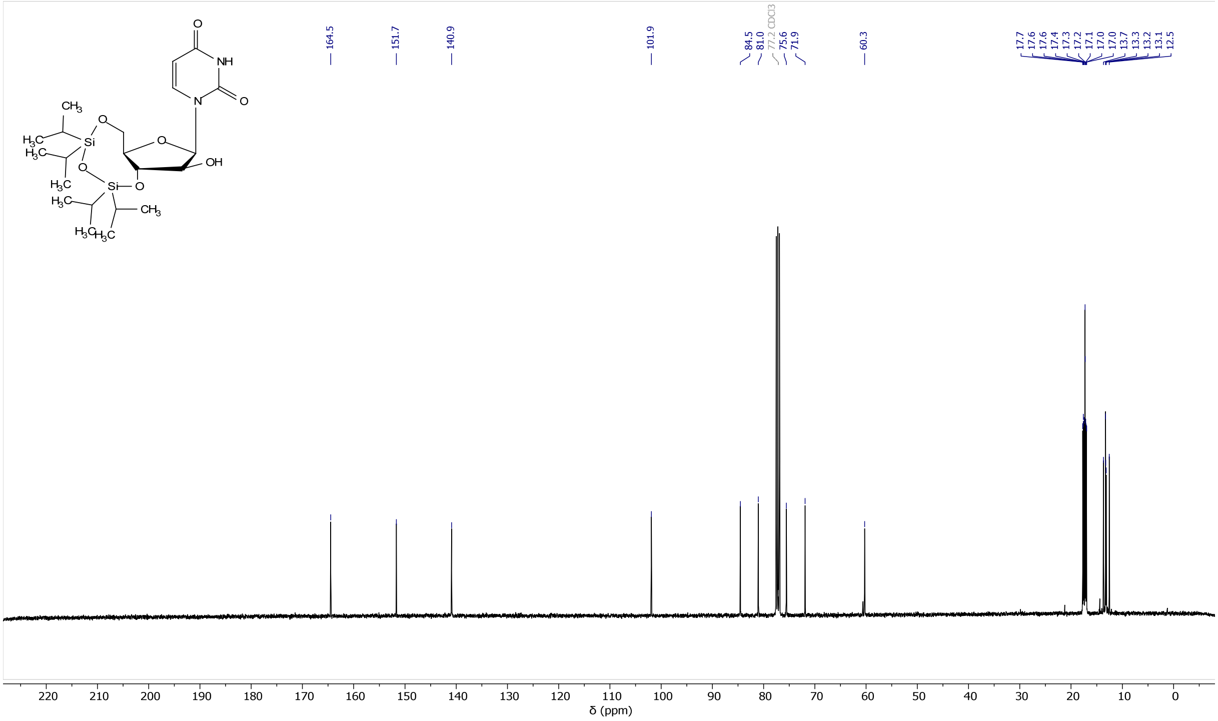

Figure S52. ^{13}**C NMR of 12 in CDCl**$_3$**.**

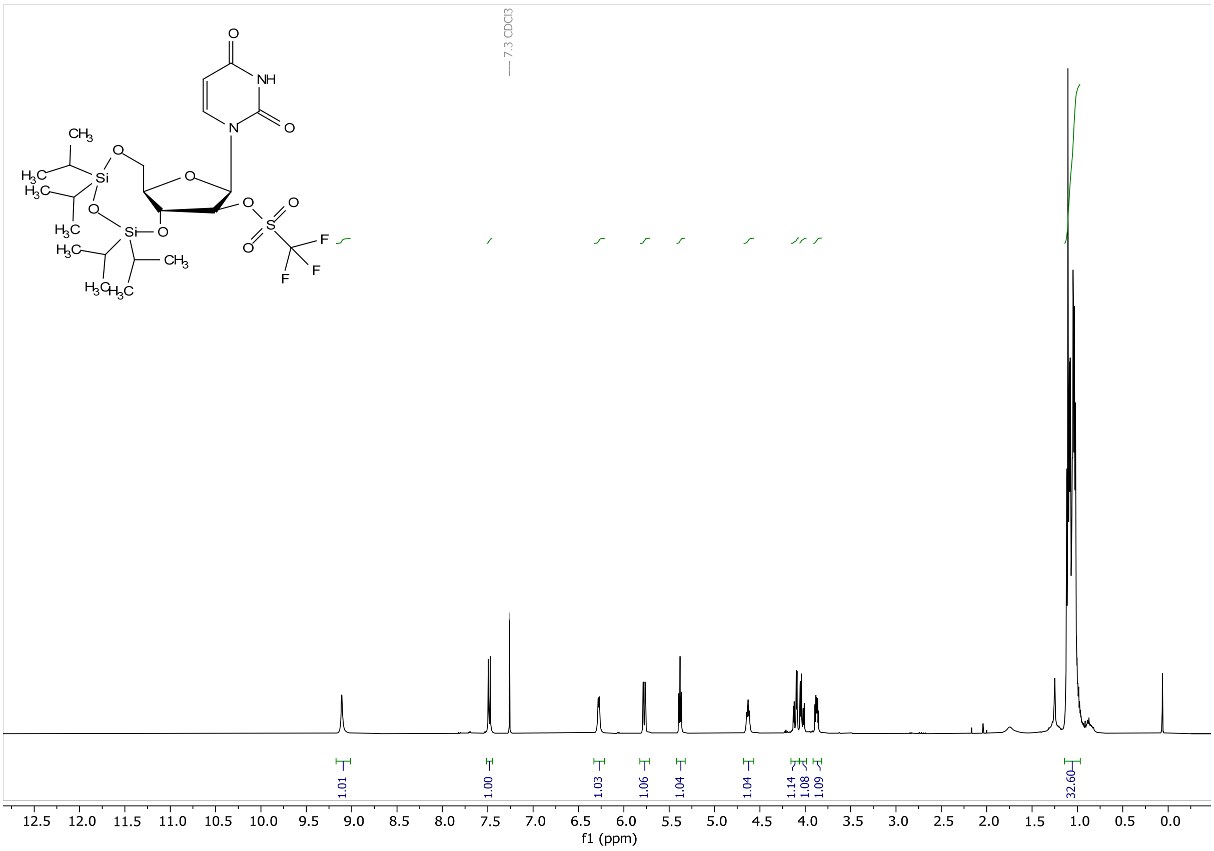

Figure S53. [1]**H NMR of 13 in CDCl3.**

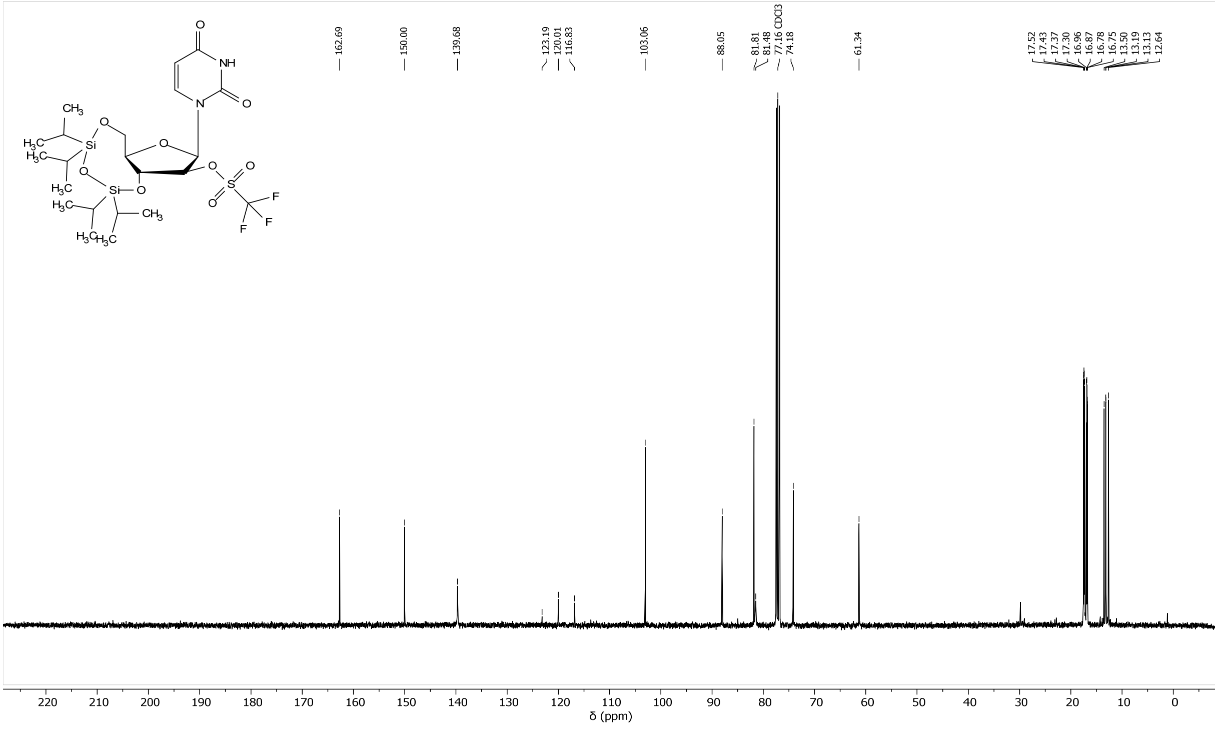

Figure S54. [13]**C NMR of 13 in CDCl3.**

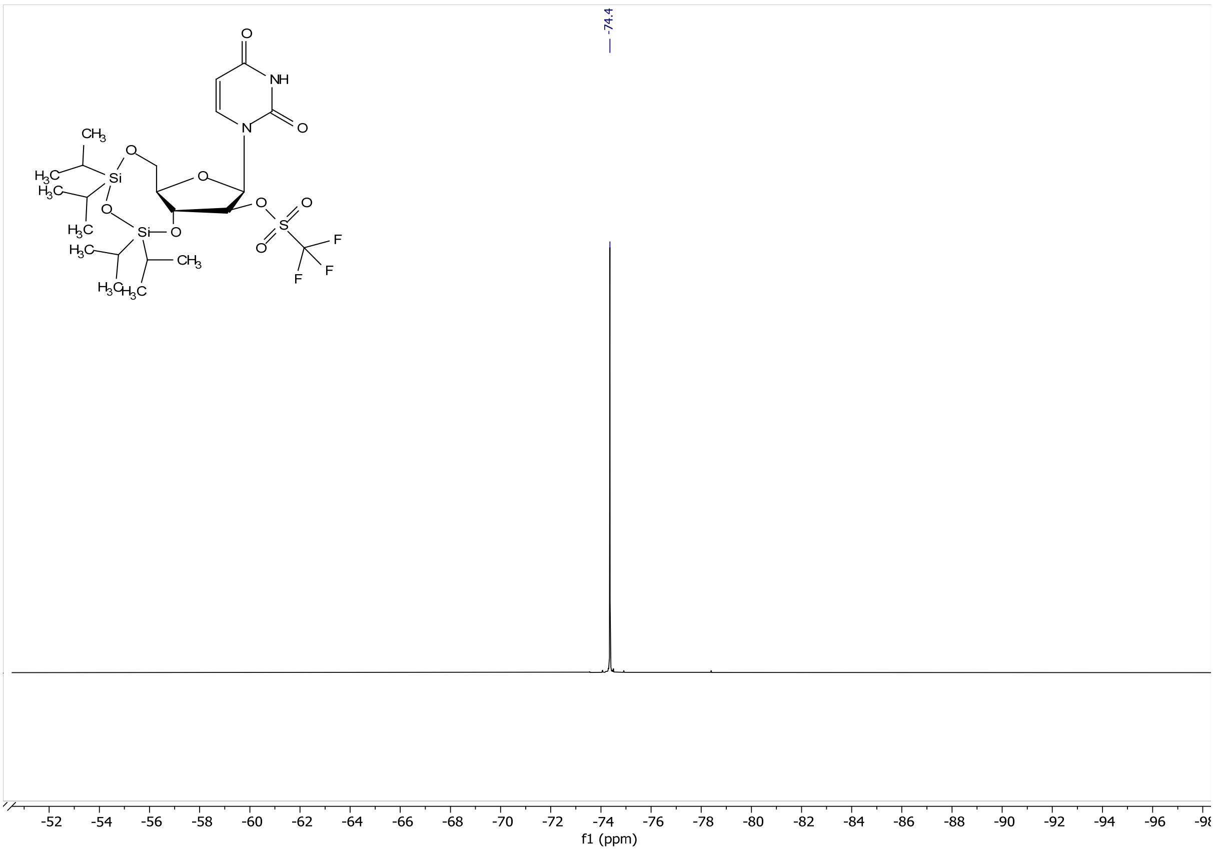

Figure S55. [19]**F NMR of 13 in CDCl₃.**

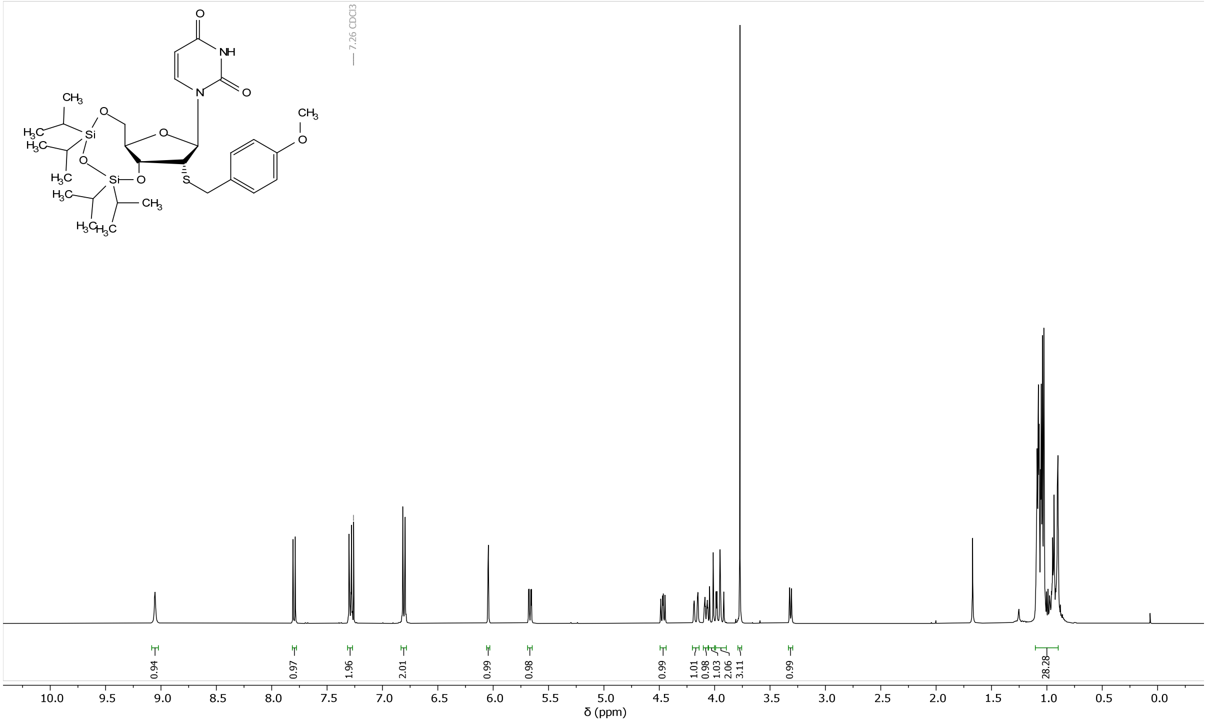

Figure S56. [1]**H NMR of 14 in CDCl₃.**

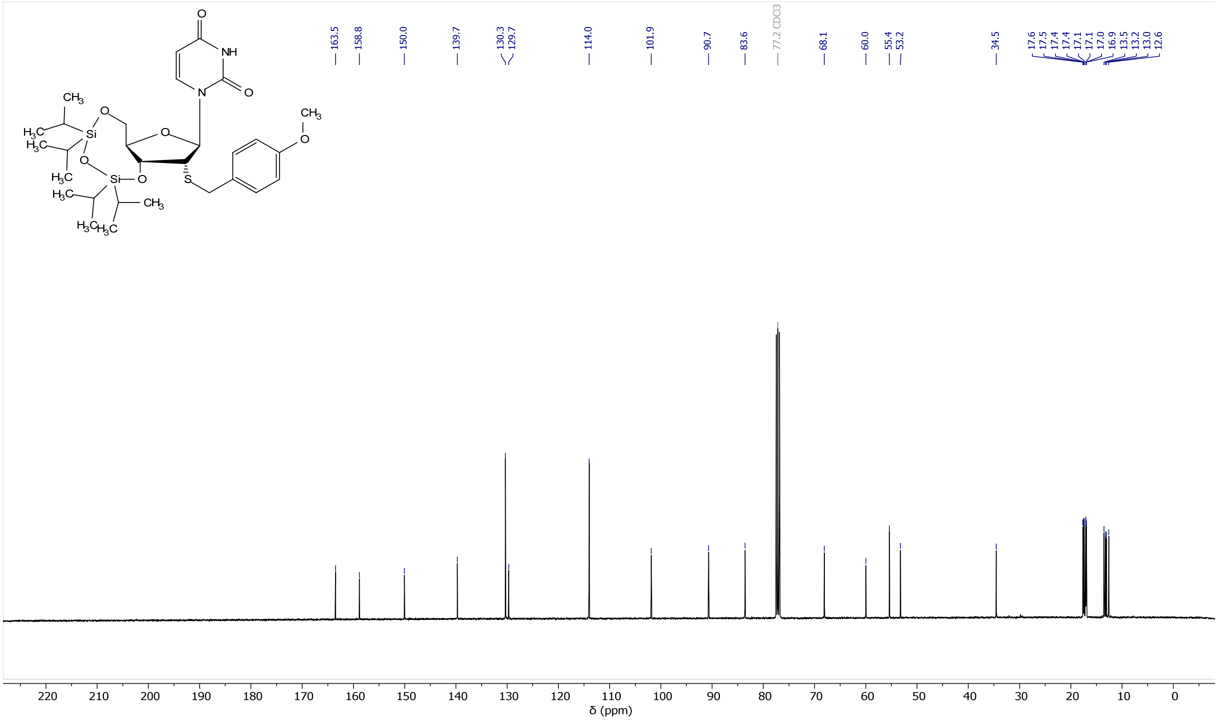

Figure S57. ^{13}C NMR of 14 in $CDCl_3$.

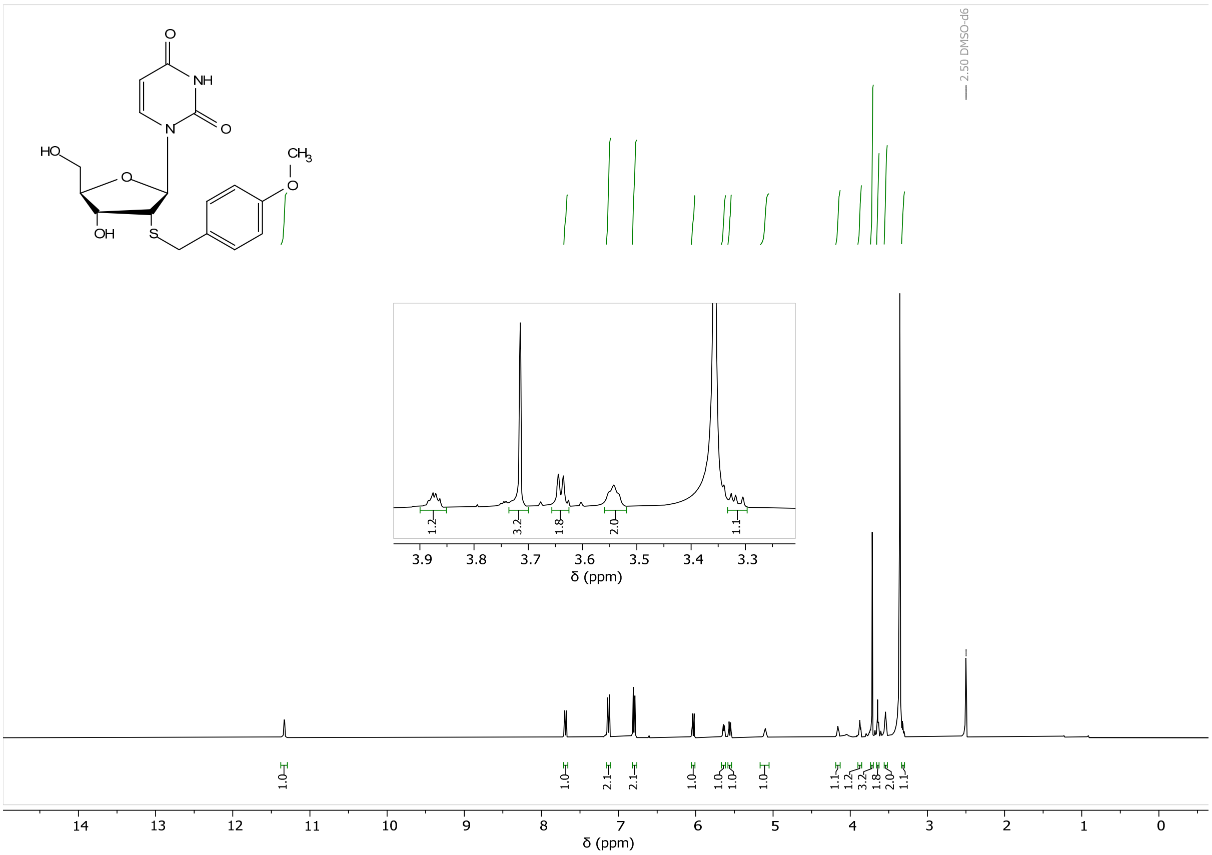

Figure S58. ^{1}H NMR of 15 in DMSO-d_6.

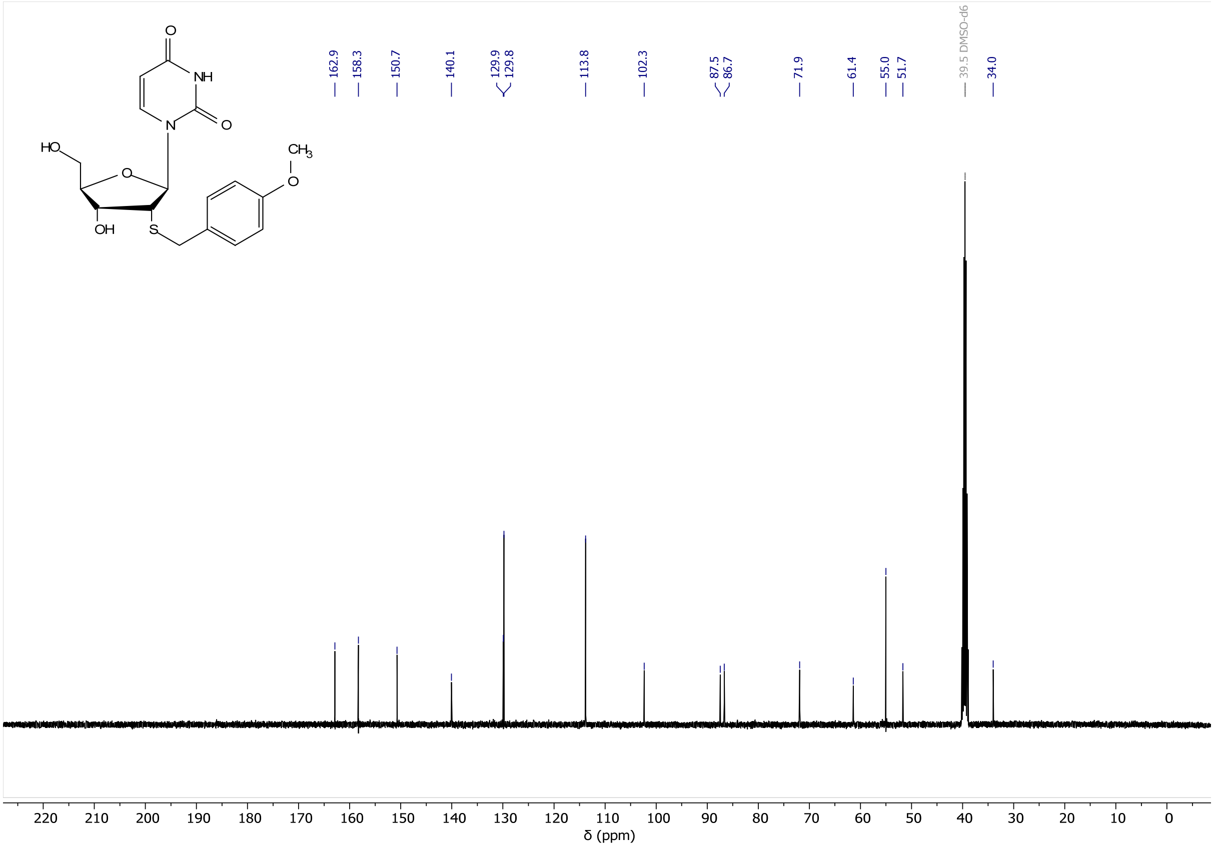

Figure S59. ^{13}**C NMR of 15 in DMSO-*d*$_6$.**

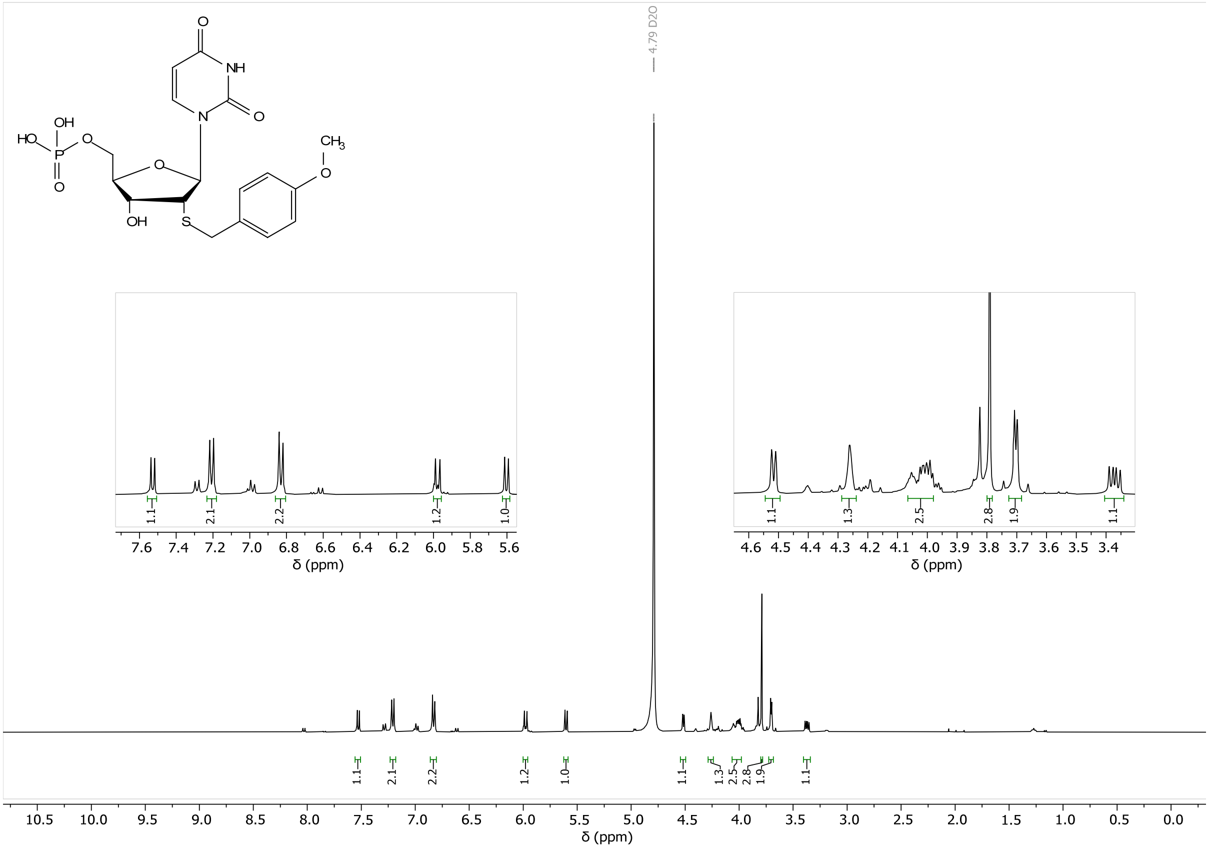

Figure S60. ^{1}**H NMR of 16 in** D_2O**.**

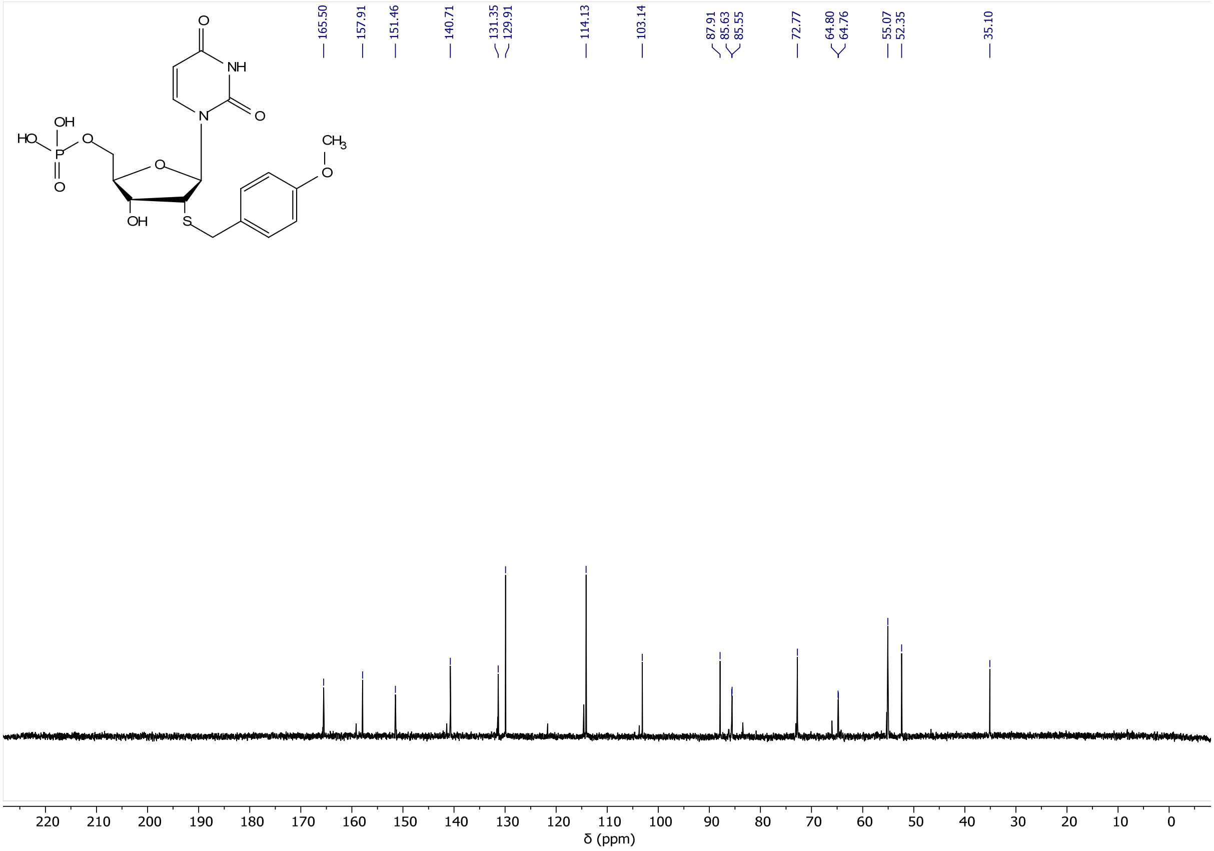

Figure S61. ^{13}**C NMR of 16 in** D_2O**.**

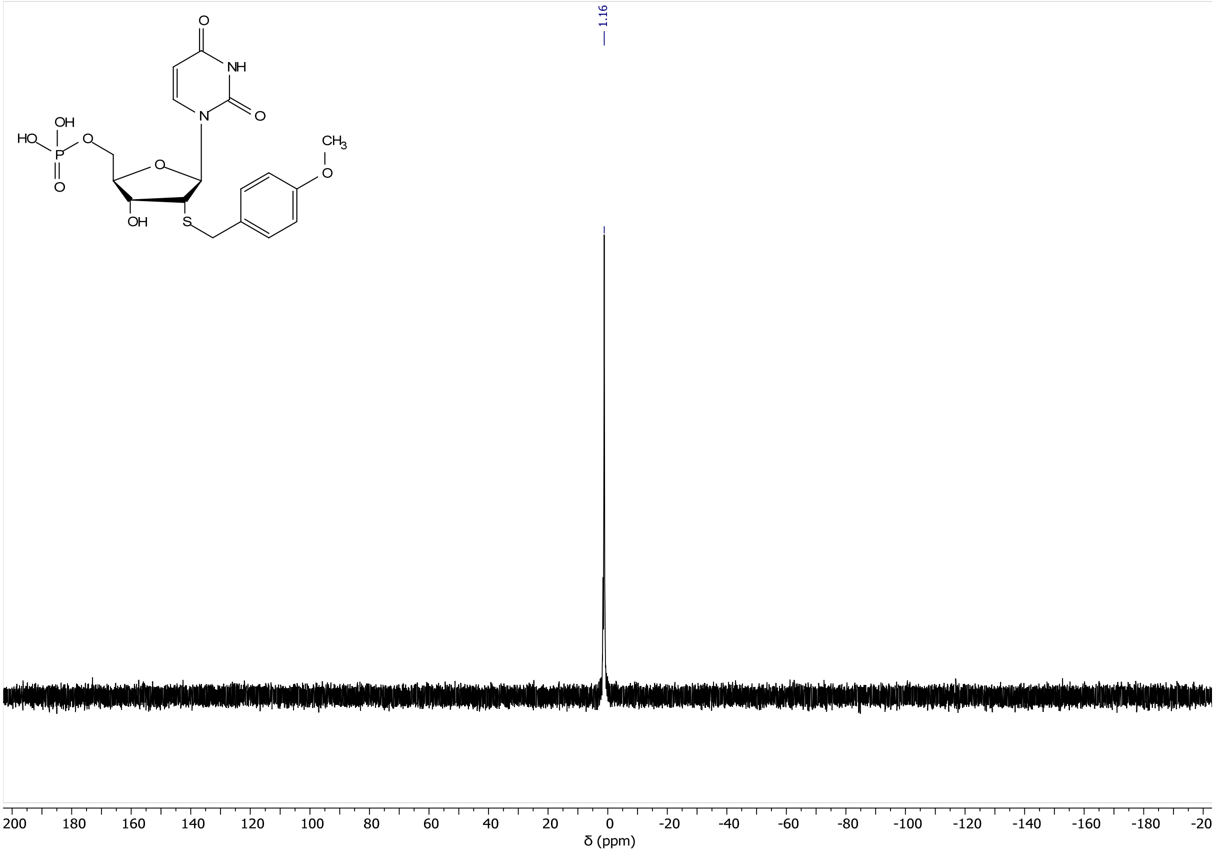

Figure S62. ^{31}**P NMR of 16 in** D_2O**.**

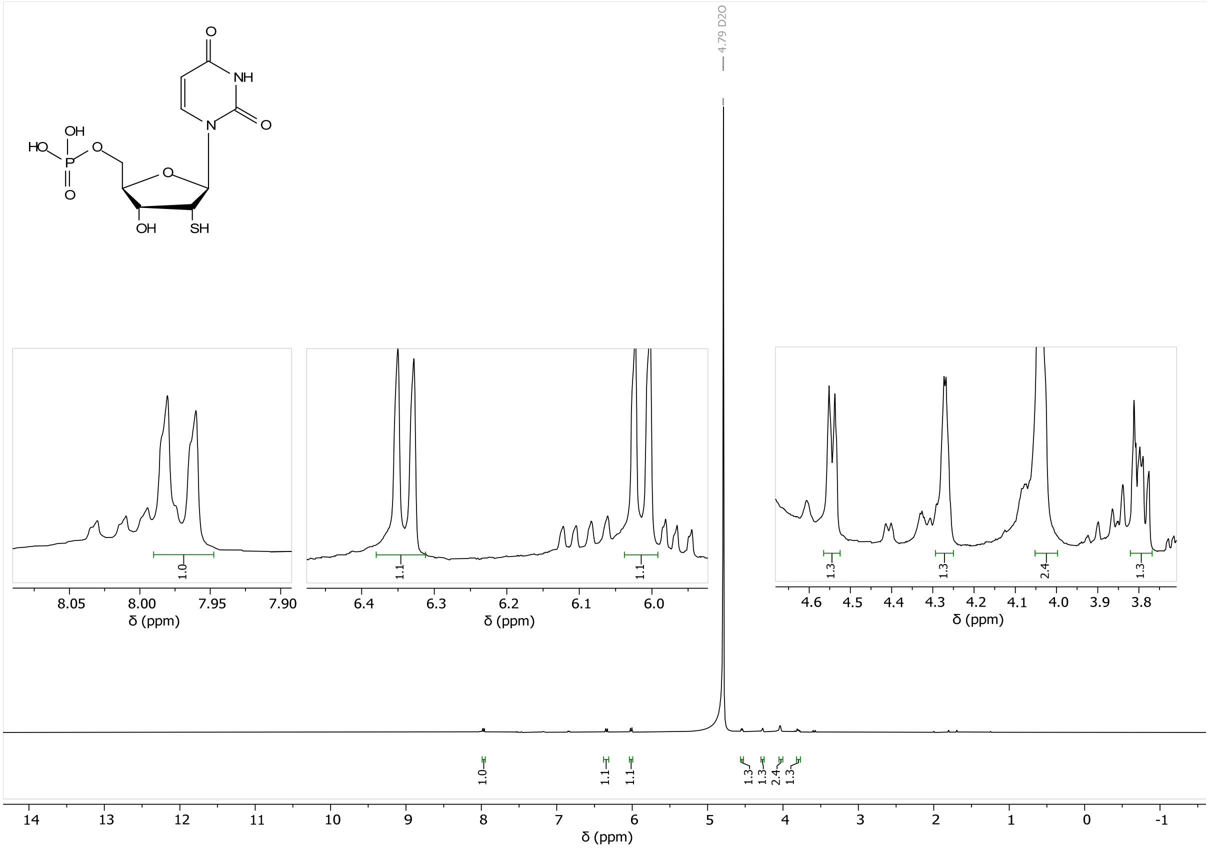

Figure S63. ^{1}**H NMR of 2'-SH UMP in D$_2$O.**

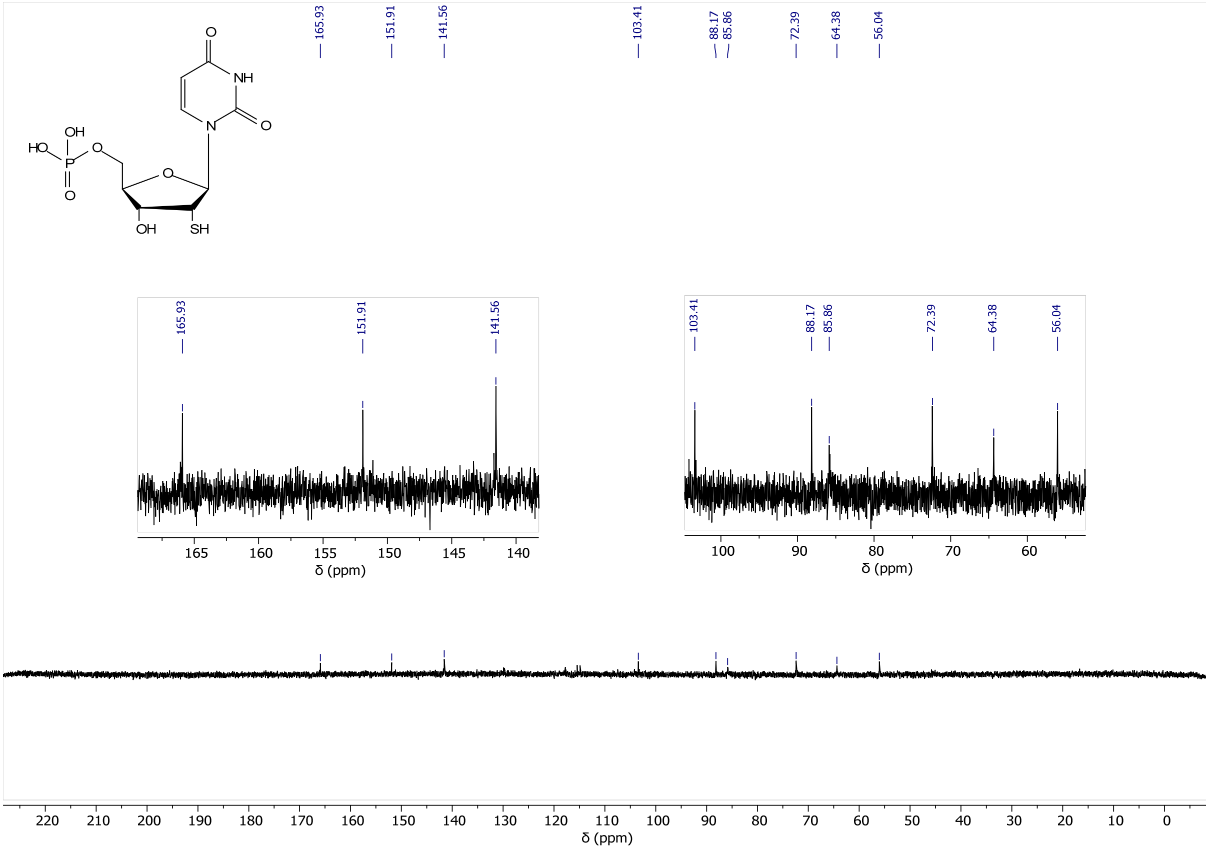

Figure S64. ^{13}**C NMR of 2'-SH UMP in D$_2$O.**

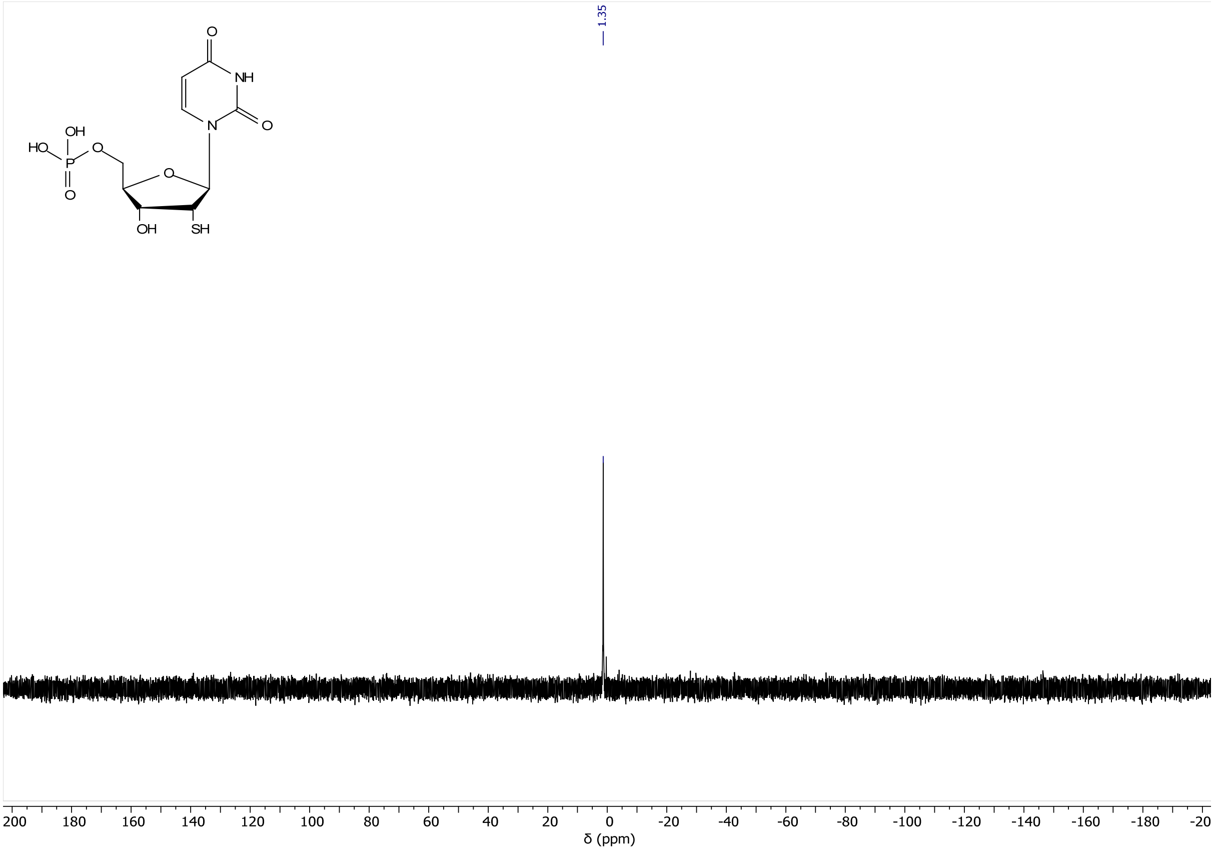

Figure S65. ^{31}P NMR of 2’-SH UMP in D_2O.

6. **Table S2.** Crystallographic statistics.

	OMPD T321N deoxy UMP	OMPD T321N UMP/dTMP	OMPD CMP	OMPD 5-Methyl UMP
PDB code	9HDT	9HDS	9HIL	9HDZ
Data collection				
Wavelength	0.6888	0.7293	0.6888	0.976
Space group	C 2 2 21	C 2 2 21	P 21 21 21	P 1 21 1
Cell dimensions				
a, b, c (Å)<br>α, β, γ (°)	78.09 116.3 62.18<br>90 90 90	85.64 117.3 60.87<br>90 90 90	62.18 75.5 119.2<br>90 90 90	69.42 61.51 71.25<br>90 112.11 90
Resolution range	32.42 - 1.3<br>(1.347 - 1.3) *	30.07 - 0.86<br>(0.8907 - 0.86) *	44.54 - 1.6<br>(1.657 - 1.6) *	42.33 - 1.795<br>(1.859 - 1.795) *
Multiplicity	6.8 (6.9) *	11.9 (8.7) *	13.1 (13.9) *	3.5 (3.6) *
Completeness (%)	99.84 (99.61) *	97.29 (82.93) *	98.52 (99.74) *	98.01 (98.73) *
I/σ (I)	12.28 (1.80) *	19.01 (1.09) *	14.31 (2.04) *	15.80 (3.66) *
R_{meas}	0.09726 (1.328) *	0.05936 (2.228) *	0.116 (2.115) *	0.07511 (0.5628) *
$CC_{1/2}$	0.999 (0.717) *	0.998 (0.448) *	0.999 (0.756) *	0.999 (0.861) *
Refinement				
Reflections used in refinement	69633 (6883) *	250716 (21201) *	73730 (7383) *	51180 (5121) *
R-work (%)	12.6	12.7	20.4	15.5
R-free (%)	15.2	13.5	22.8	18.5
Number of non-hydrogen atoms	2563	2625	4282	4743
macromolecules	2246	2256	3991	4253
ligands	20	66	66	70
solvent	297	327	249	446
Average B-factor [Å2]	18.3	15.9	37.09	25.02
macromolecules	16.64	13.7	36.7	24.23
ligands	11.79	13.18	43.21	16.82
solvent	31.3	31.46	42.32	33.37
Ramachandran statistics				
Favored [%]	98.02	96.83	98.35	98.02
Allowed [%]	1.98	3.17	1.65	1.98
Outliers [%]	0	0	0	0
R.m.s. deviations				
RMS (bonds) [Å]	0.009	0.009	0.008	0.015
RMS (angles) (°)	1.12	1.14	0.93	1.12

* Values in parentheses correspond to the highest resolution shell.

	OMPD 2'-SH UMP	OMPD dTMP	OMPD XMP	OMPD YMP
PDB code	9HDY	9HDX	9HDU	9HDV
Data collection				
Wavelength	0.6888	0.6888	0.6888	0.6888
Space group	C 2 2 21	P 1 21 1	C 2 2 21	C 2 2 21
Cell dimensions				
a, b, c (Å)<br>α, β, γ (°)	77.83 117.01 62.23<br>90 90 90	69.35 61.46 70.72<br>90 113.02 90	77.36 116.6 61.87<br>90 90 90	77.51 116.33 61.97<br>90 90 90
Resolution range	42.63 - 1.6<br>(1.657 - 1.6) *	32.54 - 1.05<br>(1.088 - 1.05) *	42.45 - 1.0<br>(1.036 - 1.0) *	32.86 - 1.5<br>(1.554 - 1.5) *
Multiplicity	7.2 (7.4) *	6.9 (7.0) *	10.2 (10.2) *	12.8 (13.0) *
Completeness (%)	96.31 (99.92) *	98.50 (98.04) *	98.83 (97.67) *	97.65 (99.89) *
I/σ (I)	10.44 (1.28) *	16.22 (1.57) *	15.69 (1.38) *	15.03 (2.06) *
R_{meas}	0.1186 (1.877) *	0.05478 (1.445) *	0.06976 (2.221) *	0.1119 (1.77) *
$CC_{1/2}$	0.998 (0.561) *	0.999 (0.682) *	0.999 (0.506) *	0.999 (0.782) *
Refinement				
Reflections used in refinement	36454 (3714) *	250370 (24840) *	148584 (14546) *	44121 (4473) *
R-work (%)	22.1	12.8	11.8	19.3
R-free (%)	24.1	14.6	13.2	21.8
Number of non-hydrogen atoms	2347	5298	2625	2444
macromolecules	2157	4687	2312	2197
ligands	32	68	36	32
solvent	169	569	289	225
Average B-factor [Å2]	26.73	17.21	15.56	25.03
macromolecules	26.3	15.51	14.04	24.24
ligands	23.78	10.31	11.72	19.3
solvent	32.53	31.73	28.03	33.33
Ramachandran statistics				
Favored [%]	98.02	98.21	98.81	98.02
Allowed [%]	1.98	1.79	1.19	1.98
Outliers [%]	0	0	0	0
R.m.s. deviations				
RMS (bonds) [Å]	0.011	0.009	0.009	0.082
RMS (angles) (°)	0.92	1.17	1.13	2.48

* Values in parentheses correspond to the highest resolution shell.

7. References

(1) Rindfleisch, S.; Krull, M.; Uranga, J.; Schmidt, T.; Rabe Von Pappenheim, F.; Kirck, L. L.; Balouri, A.; Schneider, T.; Chari, A.; Kluger, R.; Bourenkov, G.; Diederichsen, U.; Mata, R. A.; Tittmann, K. Ground-State Destabilization by Electrostatic Repulsion Is Not a Driving Force in Orotidine-5′-Monophosphate Decarboxylase Catalysis. *Nature Catalysis* **2022**, *5* (4), 332–341. https://doi.org/10.1038/s41929-022-00771-w.

(2) Salameh, H.; Afri, M.; Gottlieb, H. E.; Fischer, B. β-Cyanuryl Ribose, β-Barbituryl Ribose, and 6-Azauridine as Uridine Mimetics. *ACS Omega* **2020**, *5* (48), 31314–31322. https://doi.org/10.1021/acsomega.0c04788.

(3) Khaled, A.; Ivannikova, T.; Augé, C. Synthesis of Unnatural Sugar Nucleotides and Their Evaluation as Donor Substrates in Glycosyltransferase-Catalyzed Reactions. *Carbohydrate Research* **2004**, *339* (16), 2641–2649. https://doi.org/10.1016/j.carres.2004.09.002.

(4) Yoshikawa, M.; Kato, T.; Takenishi, T. A Novel Method for Phosphorylation of Nucleosides to 5′-Nucleotides. *Tetrahedron Letters* **1967**, *8* (50), 5065–5068. https://doi.org/10.1016/S0040-4039(01)89915-9.

(5) Miller, B. G.; Butterfoss, G. L.; Short, S. A.; Wolfenden, R. Role of Enzyme−Ribofuranosyl Contacts in the Ground State and Transition State for Orotidine 5‘-Phosphate Decarboxylase: A Role for Substrate Destabilization? *Biochemistry* **2001**, *40* (21), 6227–6232. https://doi.org/10.1021/bi0028993.

(6) Takahashi, M.; Grajkowski, A.; Cawrse, B. M.; Beaucage, S. L. Innovative 2′- *O* -Imino-2-Propanoate-Protecting Group for Effective Solid-Phase Synthesis and 2′- *O* -Deprotection of RNA Sequences. *J. Org. Chem.* **2021**, *86* (7), 4944–4956. https://doi.org/10.1021/acs.joc.0c02773.

(7) Ozaki, H.; Momiyama, S.; Yokotsuka, K.; Sawai, H. Synthesis of Oligodeoxyribonucleotides Bearing a Functional Group at 2’-Position by Post-Synthetic Modification. *Nucleic Acids Symposium Series* **1999**, *42* (1), 47–48. https://doi.org/10.1093/nass/42.1.47.
